# Endothelial transmigration hotspots limit vascular leakage through heterogeneous expression of ICAM‐1

**DOI:** 10.15252/embr.202255483

**Published:** 2022-11-16

**Authors:** Max L B Grönloh, Janine J G Arts, Sebastián Palacios Martínez, Amerens A van der Veen, Lanette Kempers, Abraham C I van Steen, Joris J T H Roelofs, Martijn A Nolte, Joachim Goedhart, Jaap D van Buul

**Affiliations:** ^1^ Molecular Cell Biology Lab, Department of Molecular Hematology Sanquin Research and Landsteiner Laboratory Amsterdam The Netherlands; ^2^ Section Molecular Cytology at Swammerdam Institute for Life Sciences, Leeuwenhoek Centre for Advanced Microscopy University of Amsterdam Amsterdam The Netherlands; ^3^ Department of Pathology, Amsterdam Cardiovascular Sciences Amsterdam UMC, University of Amsterdam, Location AMC Amsterdam The Netherlands

**Keywords:** ICAM‐1, inflammation, leakage, transendothelial migration hotspots, Cell Adhesion, Polarity & Cytoskeleton, Immunology, Vascular Biology & Angiogenesis

## Abstract

Upon inflammation, leukocytes leave the circulation by crossing the endothelial monolayer at specific transmigration “hotspot” regions. Although these regions support leukocyte transmigration, their functionality is not clear. We found that endothelial hotspots function to limit vascular leakage during transmigration events. Using the photoconvertible probe mEos4b, we traced back and identified original endothelial transmigration hotspots. Using this method, we show that the heterogeneous distribution of ICAM‐1 determines the location of the transmigration hotspot. Interestingly, the loss of ICAM‐1 heterogeneity either by CRISPR/Cas9‐induced knockout of ICAM‐1 or equalizing the distribution of ICAM‐1 in all endothelial cells results in the loss of TEM hotspots but not necessarily in reduced TEM events. Functionally, the loss of endothelial hotspots results in increased vascular leakage during TEM. Mechanistically, we demonstrate that the 3 extracellular Ig‐like domains of ICAM‐1 are crucial for hotspot recognition. However, the intracellular tail of ICAM‐1 and the 4^th^ Ig‐like dimerization domain are not involved, indicating that intracellular signaling or ICAM‐1 dimerization is not required for hotspot recognition. Together, we discovered that hotspots function to limit vascular leakage during inflammation‐induced extravasation.

## Graphical abstract

By regulating hotspots for neutrophil adhesion, heterogenous ICAM‐1 expression prevents vascular leakage during transendothelial migration of neutrophils.

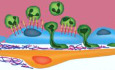

## Introduction

The migration of leukocytes towards sites of infection or tissue damage is key to the inflammatory response of the innate immunity. To reach the underlying tissue, leukocytes exit the circulation through a process called transendothelial migration (TEM). TEM consists of several subsequent steps, known as the multistep process, introduced by Butcher and Springer, and although the basis of this concept is still very solid, new details still are discovered and characterized, adding to the full picture of the multistep paradigm (Butcher, 1991; Springer, 1994; Nourshargh & Alon, 2014; Vestweber, 2015; Muller, 2016; Alon & van Buul, 2017).

It is recognized that during inflammation, when leukocytes extravasate, vessels do not leak (Vestweber *et al*, 2014; Alon & van Buul, 2017). Work by our group and others showed that the gaps induced by the penetrating leukocytes are quickly repaired by a variety of intracellular processes within the endothelium itself (Martinelli *et al*, 2013; Heemskerk *et al*, 2016; Braun *et al*, 2020). Intravital microscopy revealed that neutrophil migration through the endothelial monolayer and the basement membrane and pericyte sheath does not occur randomly, but in fact occurs at predefined exit sites, called “hotspots” (Hyun *et al*, 2019). Although it is without a doubt that leukocytes use hotspots to cross the endothelium and many factors have been proposed to determine hotspot composition and localization (Grönloh *et al*, 2021), the physiological relevance of why leukocytes would prefer to cross the endothelium at hotspots is unclear. Examples of hotspot regulators are heterogenous chemokine gradients (Gschwandtner *et al*, 2017; Martínez‐Burgo *et al*, 2019), differences in substrate stiffness (Stroka & Aranda‐Espinoza, 2009; Schimmel *et al*, 2018), junction phenotype (Gorina *et al*, 2014) and recently reported varying junctional membrane protrusion activities between individual endothelial cells (Arts *et al*, 2021) and autophagy at junction regions (Reglero‐Real *et al*, 2021). Additionally, the composition and density of the pericyte sheath and the basement membrane layer may also influence the location of both endothelial and basement membrane hotspots (Proebstl *et al*, 2012; Song *et al*, 2017).

As endothelial adhesion molecules are important regulators of efficient neutrophil TEM, this protein family may also play a role in the localization of TEM hotspots. Intercellular adhesion molecule (ICAM)‐1 and ICAM‐2, both heavily involved in neutrophil adhesion, are transmembrane glycoproteins of the immunoglobulin superfamily and in particular ICAM‐1 is highly upregulated on inflamed endothelium (Wertheimer *et al*, 1992). ICAM‐1 has several splicing variants but generally consists of 5 extracellular immunoglobulin (Ig)‐like domains, the fourth one regulating homodimerization (Chen *et al*, 2007; Ramos *et al*, 2014). ICAM‐2 has just two Ig‐like domains, which are homologs to the first and second Ig‐like domains of ICAM‐1 (Xu *et al*, 1992). Both ICAM‐1 and ICAM‐2 bind neutrophil integrins lymphocyte function‐associated antigen 1 (LFA‐1; CD11a/CD18) and macrophage‐1 antigen (Mac‐1; CD11b/CD18), and are mainly involved in the firm adhesion and crawling steps of the endothelium (Heit *et al*, 2005; Halai *et al*, 2014). LFA‐1 has been reported to bind to the first extracellular domains of ICAM‐1 and ICAM‐2 (Staunton *et al*, 1990; Li *et al*, 1993). Mac‐1 binds the third extracellular domain of ICAM‐1, whereas information on the association between Mac1 and ICAM‐2 is scarce (Xie *et al*, 1995). Both ICAM‐1 and ‐2 are linked to the actin cytoskeleton via modulators such as α‐actinin‐4, filamin B, and cortactin (Kanters *et al*, 2008; Schaefer *et al*, 2014). After inflammation, ICAM‐1 is upregulated and displays a typical patchy pattern *in vitro* and *in vivo* (Walpola *et al*, 1995; Sumagin & Sarelius, 2006; Castro Dias *et al*, 2021). In contrast to ICAM‐1, ICAM‐2 is already constitutively expressed on the endothelium in normal conditions (de Fougerolles *et al*, 1991).

The biological relevance of TEM hotspots is not yet understood. It has been hypothesized that the interaction of leukocytes with only a small selection of the vessel could help maintain the barrier integrity of the endothelium (Hyun *et al*, 2019), but no evidence in favor of this theory has been put forward. It has been shown that the transmigration of neutrophils is not correlated with local leakage at those specific sites, as mechanisms exist to limit transmigration‐induced vascular leakage (Heemskerk *et al*, 2016; Braun *et al*, 2020). Other studies have shown that leukocyte adhesion to the endothelium itself can already trigger vascular leakage (Gautam *et al*, 1998), suggesting that adhesion‐related processes, and not diapedesis‐related ones, can be associated with vascular leakage.

Here, we reveal the biological relevance of neutrophil TEM hotspots in the endothelial monolayer by establishing a molecular link between endothelial hotspots, that are regulated by ICAM‐1 distribution, and vascular leakage. Mechanistically, we show that ICAM‐1 heterogeneity is crucial for the presence of TEM hotspots. Loss of ICAM‐1 heterogeneity either by CRISPR/Cas9 knockout or distributing and expressing ICAM‐1 at equal levels in all endothelial cells results in loss of TEM hotspots but not in altered neutrophil adhesion or diapedesis efficacy. Interestingly, we found under these conditions an increase in vascular leakage during TEM. Hotspot functionality and recognition depend on the first 3 extracellular Ig‐like domains of ICAM‐1 but not on its intracellular tail or the 4^th^ Ig‐like dimerization domain. This indicates that intracellular signaling or ICAM‐1 dimerization is not required for hotspot functionality and recognition. Restoration of the heterogeneous distribution of ICAM‐1 rescued the increase in local permeability during diapedesis.

Thus, our study reveals the functional importance of endothelial heterogeneity of adhesion molecules in regulating TEM hotspots under inflammatory conditions, and these hotspots function to limit vascular leakage during leukocyte extravasation.

## Results

### Transmigration hotspots exist *in vitro*

**Figure 1:**
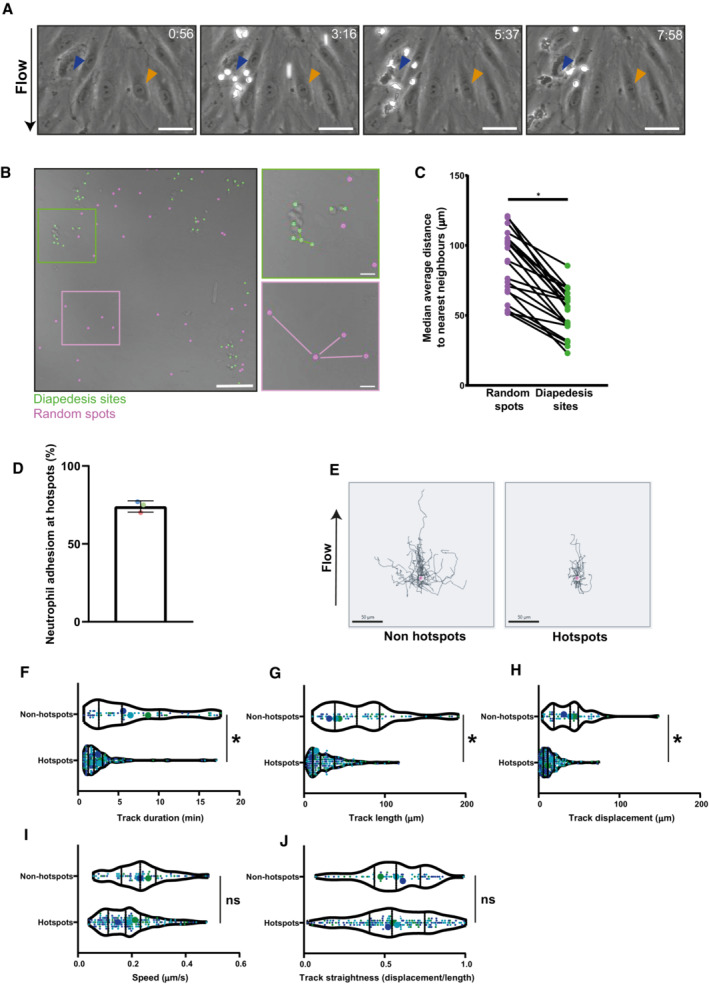
Neutrophils transmigrate more efficient at TEM hotspots AStills from a timelapse TEM assay under physiological flow with overnight TNFα‐treated HUVECs, showing a neutrophil TEM hotspot indicated with a blue arrow and a region largely ignored by neutrophils indicated with an orange arrow. The direction of flow is from top to bottom, time is indicated in minutes at the top right. Scale bar, 50 μm.BBrightfield still image from a neutrophil TEM experiment under physiological flow with TNFα‐treated HUVECs, with marked the diapedesis sites (green) and computationally generated random spots (magenta). Scale bar, 100 μm and 20 μm in zoomed images.CMedians of average distance to 3 nearest neighbors for each time lapse are plotted. Medians are paired with medians of average distance to 3 nearest neighbors of corresponding randomly generated spots. Paired *t*‐test (*n* = 21): *P < 0.0001. Each datapoint corresponds to 1 of 21 time lapses from 3 biological replicates.DBar graph of total percentage of neutrophils utilizing hotspots (≥2 diapedesis events). Means from 3 biological replicates are shown. Bar graph shows mean with SD.E40 overlayed tracks of crawling neutrophils that eventually transmigrate at a TEM hotspot (right, ≥2 diapedesis events) or not (left, 1 diapedesis event). Scale bar, 50 μm.F–JSmall dots represent individual datapoints, large dots are medians of each biological replicate. The lines in the violin plot display the quartiles and median of all datapoints. 174 hotspot tracks and 62 nonhotspot tracks from 3 biological replicates are represented in 3 different colors. Paired *t*‐test on medians (*n* = 3) of three biological replicates. (F) Violin plot of track duration of crawling neutrophils that eventually transmigrate at a TEM hotspot or not. *P = 0.0121. (G) Violin plot of total track length of crawling neutrophils that eventually transmigrate at a hotspot or not. *P = 0.039. (H) Violin plot of the total displacement (distance between the beginning and the end of the track) of crawling neutrophils that eventually transmigrate at a TEM hotspot or not. *P = 0.019. (I) Violin plot of average speed of crawling neutrophils that eventually transmigrate at a TEM hotspot or not. *P* = 0.051. (J) Violin plot of Track straightness (displacement from (F)/length from (E)) of crawling neutrophils that eventually transmigrate at a TEM hotspot or not. *P* = 0.95.

**Figure EV1:**
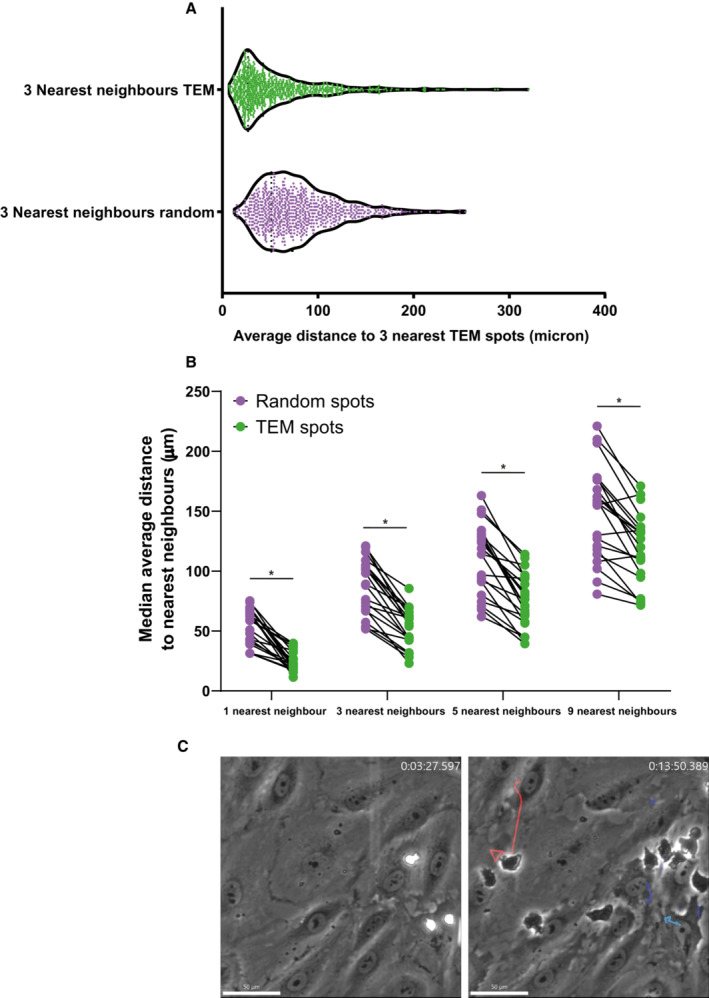
Comparison of different nearest neighbor numbers and example hotspot and non‐hotspot tracks Violin plot of average distance to 3 nearest neighbors for actual diapedesis sites and randomly generated spots. Each datapoint corresponds to 1 diapedesis site and 729 datapoints from 21 time lapses are plotted from 3 biological replicates.Comparison of analysis methods for nearest neighbor calculations. 1, 3, 5, and 9 nearest neighbor(s) for each TEM spot were calculated and compared with randomly generated spots. Data is from 21 videos from 3 biological replicates. Paired *t*‐test (*n* = 21) for every comparison: *P < 0.0001 for all.Stills from a DIC timelapse TEM assay, showing neutrophils and their complete crawling tracks at hotspots, indicated with blue tracks, and non‐hotspots, indicated with a red track. The direction of flow is from top to bottom, time is indicated in min:sec at the top right. Scale bar, 50 μm.

*In vivo* data show that leukocytes prefer local exit sites, named TEM hotspots, although the functionality of the hotspots is not clear (Hyun *et al*, 2019). To investigate the functionality of hotspots, we first need to confirm that these hotspots also exist *in vitro*. Neutrophil transmigration was studied under physiological flow conditions using tumor necrosis factor (TNF)α‐stimulated human umbilical vein endothelial cells (HUVEC). We observed that neutrophils prefer to leave the endothelium at specific sites, while almost completely ignoring other areas (Fig 1A). To quantify if more than one neutrophil preferred the same endothelial area to transmigrate, we used an unbiased “nearest neighbor” analysis, a method very suitable to detect clustering of spatial data. We calculate the mean distance of each transmigration event to three transmigration events that were nearest and compared this to the same number of randomly generated spots (Fig 1B). Indeed, a significant decrease in the average distance to the 3 nearest transmigration events compared with the randomized spots was found, indicative for the existence of preferred endothelial hotspots that regulate transmigration events (Figs 1C and EV1A). To check if the number of neighbors used in the analysis did not influence the outcome, we also analyzed transmigration events that were closest to one, five, and nine nearest transmigration events and found similar patterns (Fig EV1B). Thus, from these quantitative analyses, we conclude that TEM hotspots also exist *in vitro*.

To study whether neutrophils at TEM hotspots displayed different spatiotemporal crawling dynamics compared with neutrophils that ignored hotspots, we classified neutrophil crawling tracks in physiological flow timelapse recordings and classified a “hotspot track” when a neutrophil would transmigrate within 50 μm, the average diameter of one endothelial cell, of another neutrophil (Fig EV1C). We found that around 75% of all TEM events occurred at a hotspot location (Fig 1D). Moreover, neutrophils that used TEM hotspots crawled for shorter distances and shorter durations than neutrophils that did not undergo diapedesis at hotspots, although migration speed and crawling linearity were not altered (Fig 1E–J). Combined, these data indicate that TEM occurs more frequently and is more efficient at endothelial hotspots.

### ICAM‐1 marks neutrophil TEM hotspots

**Figure 2:**
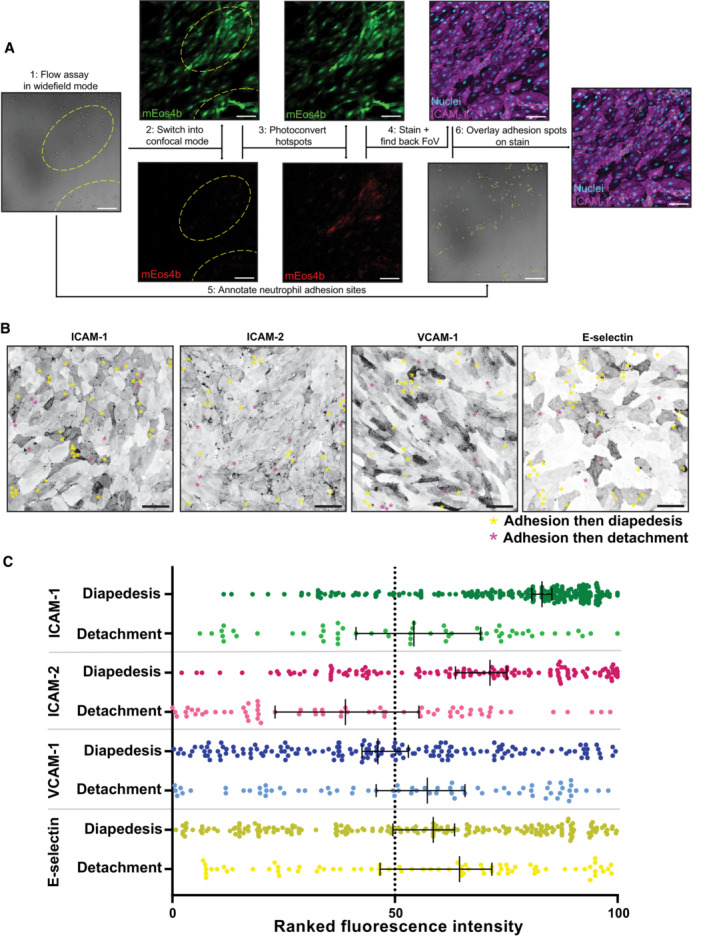
Neutrophil transmigration hotspots are located at ICAM‐1 high expressing cells Simplified workflow for photoconversion experiments. (1) Neutrophil flow assays were performed in widefield mode with overnight TNFα‐treated HUVECs expressing mEos4b. (2) The same field of views were imaged in confocal mode. (3) Areas where hotspots had appeared in the widefield video were converted from green to red using a 405 nm laser. (4) The slides were fixed and the nuclei and an adhesion molecule were stained. The original fields of view were found backing by looking for red signal. (5) In the original video, all successful and unsuccessful adhesion events were annotated. (6) The adhesion spots were overlayed with the stained image. Scale bar, 100 μm.Inverted greyscale LUT of immunofluorescence stains on overnight TNFα‐treated HUVECs for ICAM‐1, ICAM‐2, VCAM‐1, and E‐selectin of areas of which time lapses were made, relocated by photoconversion of mEos4b. With yellow asterisks, adhesion events that led to diapedesis are shown. With magenta asterisks, adhesion events that were followed by detachment are shown. Scale bar, 100 μm.Quantification of adhesion events on overnight TNFα‐treated HUVEC cells ranked by their preference for diapedesis for expression levels of ICAM‐1 (green dots, 254 diapedesis adhesion events and 57 detachment adhesion events), ICAM‐2 (magenta dots 129 diapedesis adhesion events and 79 detachment adhesion events) VCAM‐1 (blue dots 163 diapedesis adhesion events and 72 detachment adhesion events) and E‐selectin (dark yellow dots = 199 diapedesis adhesion events and 78 detachment adhesion events). Data shown are from 9 (ICAM‐1 and VCAM‐1) or 6 (ICAM‐2 and E‐selectin) images from 3 biological replicates. Median with 95% CI is shown.

To understand how neutrophils find these hotspots, we need to be able to identify endothelial TEM hotspots once neutrophils have used them. To do so, endothelial cells (ECs) were transduced with the photoconvertible probe mEos4b and neutrophil TEM under flow was monitored in real time. Sites of TEM were determined on the fly and marked as the field of view (FOV). FOV was exposed to 405 nm light, converting mEos4b to a red fluorescent protein. The red fluorescence allowed us to trace back and identify original TEM hotspots to screen for candidate adhesion molecules (Fig 2A). Using this technique, we found that the distribution of ICAM‐1 perfectly correlated with TEM hotspots, whereas VCAM‐1, ICAM‐2 and E‐selectin did not (Fig 2B).

To validate these observations, we ranked individual ECs based on their fluorescence intensity of ICAM‐1, ‐2, VCAM‐1, or E‐selectin, representing surface protein expression levels. Next, we correlated TEM sites to fluorescence intensity, plotted all TEM events, and discriminated between neutrophils that transmigrated (marked with yellow asterisks) and neutrophils that did adhere to the endothelium but detached again (marked with magenta asterisks; Fig 2B). These data showed that most adhesion events that led to successful TEM required ICAM‐1^high^ ECs (Fig 2C). Interestingly, we also found a preference for ICAM‐2^high^ ECs, albeit less prominent (Fig 2C), suggesting that neutrophils can differentiate between high and low ICAM‐2‐expressing ECs. Neutrophil adherence to VCAM‐1 was completely random (Fig 2C), underscoring the fact that neutrophils do not express VCAM‐1‐counter receptor VLA‐4. Finally, no adhesion preference for E‐selectin^high^ ECs was observed (Fig 2C), most likely because E‐selectin is involved in the rolling stage of TEM instead of the firm adhesion state. These unbiased data indicate that ICAM‐1 marks TEM hotspots.

### Heterogeneous distribution of endothelial adhesion molecules

**Figure 3:**
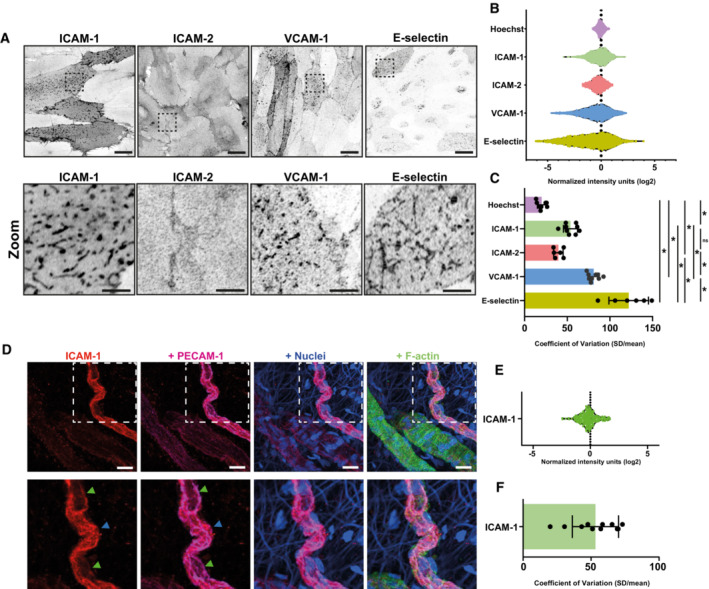
Adhesion molecules display varying degrees of heterogeneity across varying conditions Inverted greyscale LUT of IF staining for ICAM‐1, ICAM‐2, VCAM‐1, and E‐selectin on HUVECs after overnight TNFα stimulation. ROIs represent zoom regions shown below. Scale bar, 20 μm in upper panels, 5 μm in bottom panels.Violin plots showing Log2‐normalized heterogeneous expression levels of Hoechst (9 images, 1,583 cells), ICAM‐1 (9 images, 1,778 cells), ICAM‐2 (6 images, 1,097 cells), VCAM‐1 (9 images, 2,306 cells) and E‐selectin (6 images, 897 cells). Each dot represents an individual cell, and data are shown from 3 biological replicates. Data are normalized to mean intensity within an image to normalize for differences between each image. The dotted vertical line represents mean intensity.Bar graphs of the calculated coefficient of variation (CoV; standard deviation/mean) for each field of view imaged in Fig 2B. Data are shown from 3 biological replicates. Bar graphs show mean with SD. One‐way ANOVA (*n* = 9 for Hoechst, ICAM‐1, and VCAM‐1, *n* = 6 for ICAM‐2 and E‐selectin) with multiple comparison correction was performed. Hoechst vs ICAM‐2: *P = 0.0175. ICAM‐1 vs ICAM‐2: *P = 0.1214. All other combinations: *P < 0.0001.
*Ex vivo* whole‐mount stains of colonic mesenterial adipose tissue of a patient with active inflammatory bowel disease, with ICAM‐1 low (green arrow) and ICAM‐1 high (blue) cells indicated. ICAM‐1 is shown in red, PECAM‐1 in magenta, nuclei in blue, and F‐actin in green. Scale bar, 20 μm.Violin plots showing Log2‐normalized heterogeneous expression levels of ICAM‐1 (11 images, 233 cells). Each dot represents an individual cell, and data are shown from 3 biological replicates. Data are normalized to mean intensity within an image to normalize for differences between each image. The dotted vertical line represents mean intensity.Bar graph of the calculated coefficient of variation (CoV; standard deviation/mean) for each field of view imaged in Fig 2E. Data are shown from 3 biological replicates. Bar graph shows mean with SD.

Based on the strong correlation between ICAM‐1^high^ expression and TEM hotspots, we hypothesize that the heterogeneous expression of endothelial ICAM‐1 leads to increased neutrophil adhesion and subsequently diapedesis at those sites. To examine adhesion molecule distribution within an inflamed endothelial monolayer in more detail, we stained TNFα‐treated ECs for ICAM‐1, ICAM‐2, VCAM‐1, and E‐selectin and found that ICAM‐1 expression is distributed in a heterogenous manner: some ECs displayed high levels of ICAM‐1, whereas others did not (Fig 3A). Furthermore, ICAM‐1 localized to apical filopodia (Oh *et al*, 2007; Kroon *et al*, 2018), but this was only observed in ICAM‐1^high^ ECs. In contrast to ICAM‐1, ICAM‐2 showed a much more homogenous distribution within the endothelial monolayer and was slightly enriched at junction areas but not at filopodia (Fig 3A). VCAM‐1 did show heterogenous expression and was enriched in filopodia on ECs that showed VCAM‐1^high^ expression (Fig 3A). Finally, E‐selectin also was highly heterogenous and also localized towards apical filopodia in E‐selectin^high^ ECs (Fig 3A). To quantify heterogeneous distribution, we measured fluorescence intensity of individual ECs and normalized the fluorescent values within each field of view to correct for variation between images, with nuclei staining as a control for a homogenous distribution (Fig 3B). This quantification allowed us to measure the variation of protein distribution within one EC monolayer. ICAM‐1, VCAM‐1, and E‐selectin showed strong heterogenous distribution, whereas ICAM‐2 only showed minor heterogeneous distribution in an EC monolayer (Fig 3C). As ICAM‐1, VCAM‐1, and E‐selectin expression are both induced upon inflammation, we analyzed whether ICAM‐1 heterogeneity was correlated with the heterogeneous expression of the other adhesion molecules. Co‐staining of ICAM‐1 with either ICAM‐2, VCAM‐1, or E‐selectin showed no correlation between ICAM‐1 and ICAM‐2 (*r* = 0.044, *P* = 0.072; Appendix Fig S1A). A weak positive correlation was found for ICAM‐1 and VCAM‐1 (*r* = 0.532, *P* < 0.001; Appendix Fig S1B), even though we also observed significant populations of ICAM‐1^high^/VCAM‐1^low^ and ICAM‐1^low^/VCAM‐1^high^ ECs (Appendix Fig S1). ICAM‐1 and E‐selectin also showed a degree of correlation (*r* = 0.577, *P* < 0.001), where the highest ICAM‐1 ECs usually also were E‐selectin^high^ (Appendix Fig S1C).

**Figure EV2:**
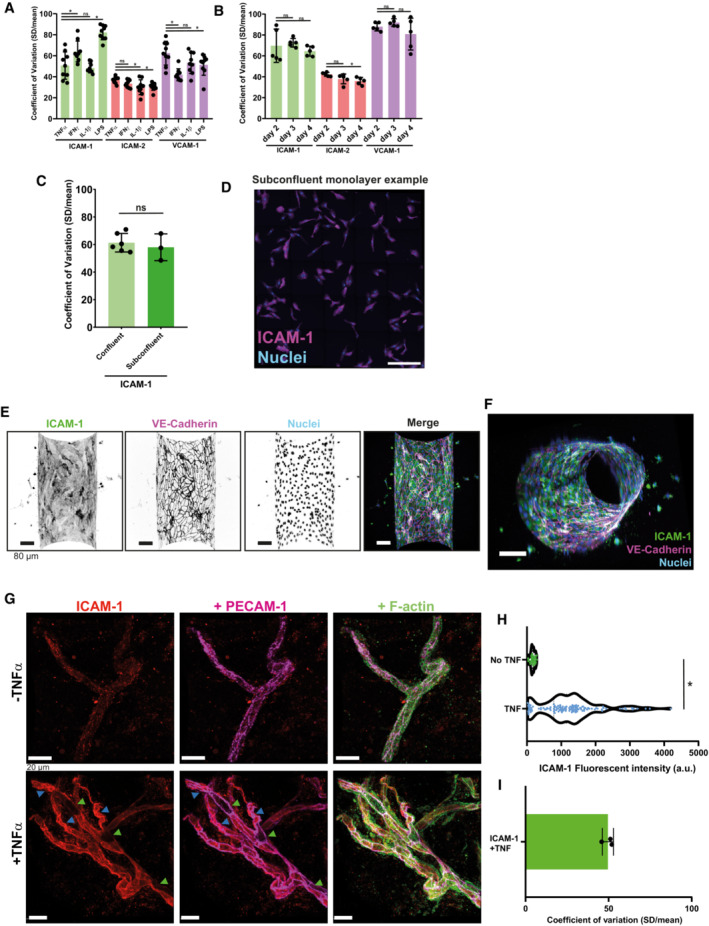
ICAM‐1 heterogeneity persists across several variables A, BBar graphs displaying coefficient of variations (CoV) of ICAM‐1, ICAM‐2, and VCAM‐1 for each field of view measured. Data are shown from 3 biological replicates. Bars show mean with SD. (A) Different inflammatory stimulants, all overnight incubated. One‐way ANOVA on means with multiple comparison correction against TNFα data, separate test for each protein (*n* = 9 images per condition). ICAM‐1 (TNFα vs IFN‐γ: *P = 0.0102. TNFα vs IL‐1β: *P* = 0.9171. TNFα vs LPS: *P < 0.0001). ICAM‐2 (TNFα vs IFN‐γ: *P* = 0.2900. TNFα vs IL‐1β: *P = 0.0107. TNFα vs LPS: *P = 0.0224). VCAM‐1 (TNFα vs IFN‐γ: *P = 0.0002. TNFα vs IL‐1β: *P* = 0.1192. TNFα vs LPS: *P = 0.0499). (B) Different maturation states of the endothelial monolayer were treated overnight with TNFα. One‐way ANOVA on means with multiple comparison correction against day 2 data, separate test for each protein (*n* = 5). ICAM‐1 (day 2 vs day 3: *P* = 0.8627. day 2 vs day 4: *P* = 0.6332.) ICAM‐2 (day 2 vs day 3: *P* = 0.1338. day 2 vs day 4: *P = 0.0262.) VCAM‐1 (day 2 vs day 3: *P* = 0.7364. day 2 vs day 4: *P* = 0.4043).CBar graphs displaying coefficient of variation (CoV) of ICAM‐1 expression in confluent HUVECs (6 images) versus subconfluent HUVECs (3 5 × 5 tile scans), treated overnight with TNFα, for each field of view measured. Confluent monolayer Mann–Whitney test (*n* = 6 for confluent and *n* = 3 for subconfluent): *P* = 0.5476. Data are shown from 3 biological replicates. Bars show mean with SD.DExample of a 5 × 5 tile scan with subconfluent HUVECs, treated overnight with TNFα. ICAM‐1 is shown in magenta and nuclei are shown in blue. Scale bar, 300 μm.EInverted greyscale LUT of IF stain for ICAM‐1, VE‐cadherin, and nuclei of a TNFα‐treated vessel‐on‐a‐chip composing of HUVECs. Scale bar, 80 μm. Only the bottom half of the Z‐stack is shown.FSide view of whole vessel‐on‐a‐chip, shown in Fig EV2D. Stained for ICAM‐1 (green), VE‐cadherin (magenta), and nuclei (blue). Scale bar, 20 μm.G
*Ex vivo* whole‐mount stains of healthy mesenterial adipose tissue of a carcinoma patient, incubated without or with TNFα for 4 h. ICAM‐1 low (green arrow) and ICAM‐1 high (blue) cells indicated. ICAM‐1 is shown in red, PECAM‐1 in magenta, and F‐actin in green. Scale bar, 20 μm.HViolin plot showing the fluorescent intensity of ICAM‐1 in *ex vivo* whole‐mount stains of healthy mesenterial adipose tissue of a carcinoma patient, incubated without or with TNFα for 4 h. Each datapoint corresponds to 1 EC and 32 (no TNFα) and 123 (with TNFα) datapoints are plotted from 3 biological replicates. Mann–Whitney test (*n* = 32 for no TNFα and *n* = 123 for with TNFα): *P < 0.0001.IBar graph of the calculated coefficient of variation (CoV; standard deviation/mean) for each field of view image in Fig EV2H. Data are shown from 3 biological replicates. Bar graph shows mean with SD.

To study whether our findings were specific to TNFα treatment, we treated ECs with other inflammatory mediators such as Lipopolysaccharide (LPS), Interferon (IFN)‐γ, and Interleukin (IL)‐1β. The results revealed that under any inflammatory stimulus tested, ICAM‐1 and VCAM‐1 showed strong heterogeneous distribution whereas ICAM‐2 only showed minor heterogeneous distribution (Fig EV2A). To explore whether the heterogeneous distribution of adhesion molecules in the inflamed EC monolayer changed over time, we allowed EC monolayers to mature for multiple days, ranging from 2 to 4 days, before treating with TNFα. No change in the degree of heterogeneity of any of the adhesion molecules measured was found (Fig EV2B). To study whether ICAM‐1 heterogeneous expression is inherent to single ECs, or whether cell–cell contacts may influence ICAM‐1 expression of neighboring cells, we compared confluent monolayers with single‐cell seeded inflamed HUVECs. Even in subconfluent ECs, similar heterogeneous ICAM‐1 expression was measured, indicating that ICAM‐1 expression is not influenced by cell–cell contacts (Fig EV2C and D).

Using a vessel‐on‐a‐chip model, developed by our lab (Van Steen *et al*, 2021), we confirmed ICAM‐1 heterogeneous distribution in a 3D TNFα‐inflamed vessel (Fig EV2E and F). Clinically obtained samples of the chronically inflamed human mesentery of inflammatory bowel disease patients showed ICAM‐1 cell‐to‐cell heterogeneity in small veins (Fig 3D). The ICAM‐1 heterogeneity observed in these microvessels was comparable to the heterogeneity observed in our *in vitro* assays (Fig 3E and F). Noninflamed control tissue of the same organ, derived from intestinal carcinoma patients showed no ICAM‐1 expression, but *ex vivo* treatment with TNFα for 4 h showed upregulation and heterogeneous distribution of ICAM‐1 (Fig EV2G–I). Together, these data show that heterogeneity of adhesion molecules is broadly conserved upon different conditions.

**Figure 4:**
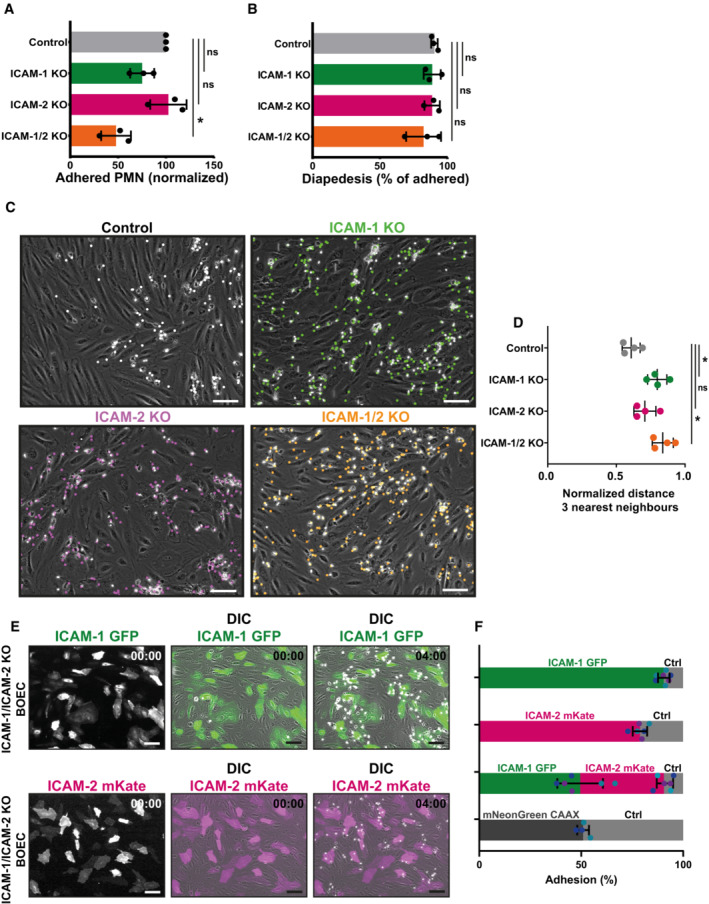
ICAM‐1 is the major marker for neutrophil hotspots Quantification of number of adhered neutrophils (PMN) in TEM under flow assay using control BOECs (no gRNA), ICAM‐1 KO BOECs, ICAM‐2 KO BOECs, and double ICAM‐1/2 KO BOECs. Data are normalized to control conditions (100%). Data consists of 3 biological replicates, 27,141 total neutrophils measured. Bar graph displays mean with SD. One‐way paired ANOVA (*n* = 3) with multiple comparison correction, comparing all conditions with control. Control vs ICAM‐1 KO: *P* = 0.6139. Control vs ICAM‐2 KO: *P* = 0.9725. Control vs ICAM‐1/2 KO: *P = 0.0296.Quantification of diapedesis efficacy (total transmigrated / total neutrophils detected *100%) of neutrophils through control, ICAM‐1 KO, ICAM‐2 KO, and ICAM‐1/2 KO BOECs. Data consists of 3 biological replicates, 27,141 total neutrophils measured. Bar graph displays mean with SD. One‐way paired ANOVA (*n* = 3) with multiple comparison correction, comparing all conditions with control. Control vs ICAM‐1 KO: *P* = 0.9679 Control vs ICAM‐2 KO: *P* = 0.9991. Control vs ICAM‐1/2 KO: *P* = 0.2281.Stills from neutrophil flow time lapses over control, ICAM‐1 KO, ICAM‐2 KO, and ICAM‐1/2 KO BOECs. All neutrophil TEM spots that occurred in the time lapse are shown in gray (control), green (ICAM‐1 KO), magenta (ICAM‐2 KO), and ICAM‐1/2 KO (orange). Scale bar, 100 μm.Medians of average distance of adhesion sites or TEM sites to 3 nearest neighbors, normalized against medians of the average distance to three nearest neighbors of the corresponding randomly generated spots. Data from 4 biological replicates are shown. One‐way paired ANOVA (*n* = 4) with multiple comparison correction, comparing all conditions with control. Control vs ICAM‐1: *P = 0.0150. Control vs ICAM‐2: *P* = 0.2077. Control vs ICAM‐1/2 KO: *P = 0.0049.Timelapse imaging of TEM under flow with ICAM‐1/ICAM‐2 KO cells. Part of EC monolayer is rescued with ICAM‐1‐GFP (green) or ICAM‐2‐mKate (magenta). Time indicated in the upper right corner in minutes. Left panels show ICAM‐1‐ or ICAM‐2‐only channel, middle, and right channels are merged with DIC. White dots are adhering neutrophils. Scale bar, 100 μm.Quantification of the preference for neutrophils to adhere to ICAM‐1‐GFP (green), ICAM‐2‐mKate (magenta), or CAAX‐mNeonGreen (dark gray) expressed in ICAM‐1/ICAM‐2 KO ECs or ICAM‐1/ICAM‐2 KO ECs (Ctrl; light gray). Bars represent percentage of neutrophil that adheres to indicated cell type. Numbers are corrected for the area occupied. Dots are percentages from individual timelapse images, colors represent data from 3 biological replicates. Bars represent mean with standard deviation.

### ICAM‐1 heterogeneity determines TEM hotspots

The functional existence of TEM hotspots is not clear. To understand this better, we focused on the major TEM hotspot marker ICAM‐1 and generated stable ICAM‐1 knockout (KO) ECs using Crispr/Cas9. In addition, we also generated ICAM‐2 and ICAM‐1/2 double knockout ECs. As HUVECs are limited by lifespan and passage time, we used blood outgrowth endothelial cells (BOECs) isolated from umbilical cord blood. These cells correspond to the characteristics ascribed to ECs and can be kept in culture for several passages (Martin‐Ramirez *et al*, 2012). Successful KO of ICAM‐1 and ‐2 in ECs under TNFα stimulation was confirmed by Western blotting (Appendix Fig S2A–C) and sequencing (Appendix Fig S2D). ICAM‐1 KO did not influence ICAM‐2 distribution compared with control ECs and ICAM‐2 KO ECs still showed ICAM‐1 heterogeneity (Appendix Fig S3A and B). Surprisingly, we found only a slight, nonsignificant decrease in neutrophil adhesion to ICAM‐1‐deficient ECs under flow conditions (Fig 4A). These data are in line with studies that used blocking antibodies against ICAM‐1 (Shang & Issekutz, 1998; Issekutz *et al*, 1999; Avellino *et al*, 2004). Depletion of ICAM‐2 also did not alter neutrophil adhesion (Fig 4A). Interestingly, double KO ECs did show a 50% reduction in adhesion (Fig 4A). We did not find any effects on neutrophil diapedesis efficacy, as consistently around 80% of adhered neutrophils underwent diapedesis, indicating that ICAM‐1 and/or ‐2 are not directly regulating neutrophil diapedesis (Fig 4B). Additionally, no effect on neutrophil crawling length, duration or speed was measured in any of the conditions (Appendix Fig S4A–C). Interestingly, when quantifying TEM hotspot events, we found a loss of TEM hotspots for neutrophils that adhered and crossed ICAM‐1^KO^ but not to ICAM‐2^KO^ EC monolayers (Fig 4C and D).

To confirm that the heterogeneous distribution of ICAM‐1 is crucial to induce TEM hotspots, we rescued the heterogeneous distribution of ICAM‐1 in EC monolayers by overexpressing ICAM‐1 in ICAM‐1/ICAM‐2 double KO ECs in a mosaic fashion (Fig 4E; Appendix Fig S5). Interestingly, 90% of all neutrophils adhered to ICAM‐1‐GFP‐expressing ECs but not to the KO ECs (Fig 4F). Neutrophils also showed a preference for ICAM‐2‐expressing ECs, albeit less prominent compared with ICAM‐1 (Fig 4F). Combining ICAM‐1 and ICAM‐2 heterogeneity showed that there was a small preference for ICAM‐1 over ICAM‐2 (Fig 4F). The membrane‐marker CAAX was used as a control and did not affect the preference for adhesion (Fig 4F). To investigate whether ICAM‐1 by itself is enough to induce neutrophil adhesion, we rescued ICAM‐1 GFP in ICAM‐1 KO BOECs and compared neutrophil adhesion to ECs treated with and without TNFα. Without TNFα, neutrophils were unable to adhere to the monolayer (Appendix Fig S6), indicating that other TNFα‐induced factors are essential on top of heterogenous ICAM‐1 for the formation of hotspots. These data indicate that ICAM‐1 triggers TEM hotspots in the inflamed endothelium.

### TEM hotspots function to limit vascular leakage during TEM

**Figure EV3:**
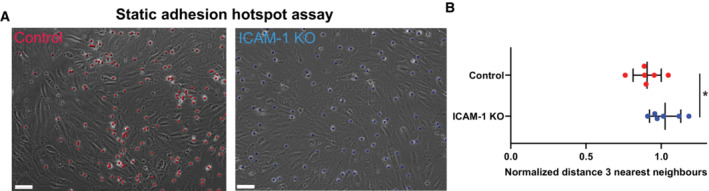
Neutrophil adhesion hotspots exist in nonflow conditions DIC images of Control and ICAM‐1 KO BOECs, treated overnight with TNFα and treated 5 min with neutrophils to allow firm adhesion. Red dots show location of adhered neutrophils on control BOECs, and blue dots show location of adhered neutrophils on ICAM‐1 KO BOECs. Scale bar, 70 μm.Medians of average distance of adhesion sites or TEM sites to 3 nearest neighbors, normalized against medians of the average distance to three nearest neighbors of the corresponding randomly generated spots. Data from 6 biological replicates are shown. Mann–Whitney: *P = 0.0411.

One of the consequences of TEM hotspots is that fewer areas in the EC monolayer are penetrated by transmigrating neutrophils and consequently, the EC monolayer integrity can be maintained. Therefore, we hypothesized that TEM hotspots function to limit vascular leakage during TEM events. To test this, we measured permeability and neutrophil TEM simultaneously across ICAM‐1/2 single and double KO ECs using Transwell systems. To ensure ICAM‐1‐mediated hotspots still form under no‐flow conditions, we performed a neutrophil adhesion assay on control and ICAM‐1 KO BOECs without flow. On control BOECs, neutrophils indeed still adhered in a hotspot‐like manner, whereas hotspot‐like adhesion was lowered on ICAM‐1 deficient endothelium (Fig EV3A and B).

**Figure 5:**
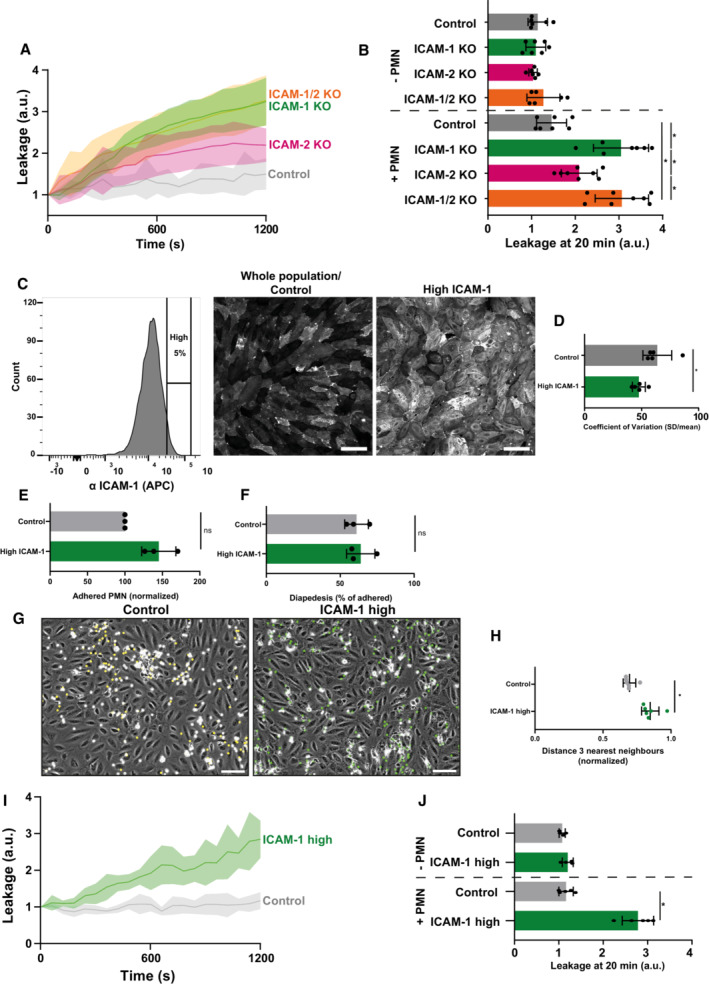
ICAM‐1 induced hotspots prevent vascular leakage Texas‐Red‐dextran extravasation kinetics through control (gray), ICAM‐1 KO (green), ICAM‐2 KO (magenta), and ICAM‐1/2 KO (orange) BOECs, treated overnight with TNFα, cultured on 3‐μm pore permeable filters. DiO‐stained neutrophils transmigrated towards C5a located in the lower compartment. Lines show means with 95% CIs of a total of 6 to 8 wells from 3 biological replicates.Quantification of Texas‐Red‐dextran extravasation kinetics through control, ICAM‐1 KO, ICAM‐2 KO, and ICAM‐1/2 KO BOECs after 20 min. Bar graphs represent mean and SD. One‐way ANOVA (*n* = 3) with multiple comparison corrections was performed within both the conditions without and with neutrophils. Without neutrophils (Control vs ICAM‐1 KO: *P* = 0.9893, Control vs ICAM‐2 KO: *P* = 0.8830, Control vs ICAM‐1/2 KO: *P* = 0.7988, ICAM‐1 KO vs ICAM‐2 KO: *P* = 0.9689, ICAM‐1 KO vs ICAM‐1/2 KO: *P* = 0.5993, ICAM‐2 KO vs ICAM‐1/2 KO: *P* = 0.3768). With neutrophils (Control vs ICAM‐1 KO: *P < 0.0001, Control vs ICAM‐2 KO: *P* = 0.1082, Control vs ICAM‐1/2 KO: *P < 0.0001, ICAM‐1 KO vs ICAM‐2 KO: *P = 0.0068, ICAM‐1 KO vs ICAM‐1/2 KO: *P* = 0.9999, ICAM‐2 KO vs ICAM‐1/2 KO: *P = 0.0042).FACS graph displaying the top 5% sorted cells with the highest ICAM‐1 expression levels based on fluorescence intensity. These cells are now called ICAM‐1 high. Example images of reseeded whole population/control HUVECs and ICAM‐1 high HUVECs are shown and stained for ICAM‐1. Scale bar, 100 μm.Bar graphs of the calculated coefficient of variation (CoV; standard deviation/mean) for control HUVECs (5 images) versus ICAM‐1 high sorted HUVECs (5 images), treated overnight with TNFα. Data are shown from 5 technical replicates. Bar graphs show mean with SD. Mann–Whitney (*n* = 5): *P = 0.0159.Quantification of number of adhered neutrophils (PMN) in TEM under flow assay using control HUVECs and ICAM‐1 high HUVECs. Data are normalized to control conditions (100%). Data consists of 3 biological replicates, 25,646 total neutrophils measured. Bar graph displays mean with SD. Paired *t*‐test (*n* = 3): *P* = 0.0754.Quantification of transmigration efficacy of neutrophils (PMN; total transmigrated / total neutrophils detected *100%) through control HUVECs and ICAM‐1 high HUVECs. Data are normalized to control conditions (100%). Data consist of 3 biological replicates, 25,646 total neutrophils measured. Bar graph displays mean with SD. Paired *t*‐test (*n* = 3): *P* = 0.7923.Stills from neutrophil flow time lapses over control, and ICAM‐1 high HUVECs, treated overnight with TNFα. All neutrophil TEM spots that occurred in the time lapse are shown in yellow (control) and green (ICAM‐1 high). Scale bar, 100 μm.Medians of average distance of adhesion sites or TEM sites to 3 nearest neighbors, normalized against medians of the average distance to the three nearest neighbors of the corresponding randomly generated spots. Data of 6 videos from 3 biological replicates are shown. Mann–Whitney test (*n* = 6): *P = 0.0022.Texas‐Red‐dextran extravasation kinetics through control (gray) and ICAM‐1 high sorted (green) HUVECs, treated overnight with TNFα, cultured on 3‐μm pore permeable filters DiO‐stained neutrophils transmigrated towards C5a located in the lower compartment. Lines show means with 95% CIs of a total of 4 (without PMNs) and 8 (with PMNs) wells from 4 biological replicates.Quantification of Texas‐Red‐dextran extravasation kinetics through control and ICAM‐1 high sorted HUVECs at 20 min. Bar graph displays mean with SD. Paired *t*‐test (*n* = 4 without neutrophils, *n* = 8 with neutrophils) was performed within conditions without and with neutrophils. Without neutrophils (Control vs ICAM‐1 high: *P* = 0.1271). With neutrophils (Control vs ICAM‐1 high: *P < 0.0001).

**Figure EV4:**
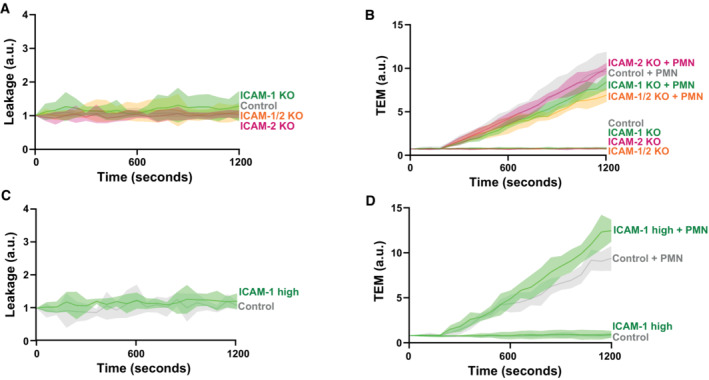
ICAM‐1/2 KO and homogenously sorted ICAM‐1 do not show basal leakage Basal leakage measured with Texas‐Red‐dextran extravasation kinetics through control (gray), ICAM‐1 KO (green), ICAM‐2 KO (magenta), and ICAM‐1/2 KO (orange) BOECs, treated overnight with TNFα. Lines show means with 95% CIs of a total of 6 to 8 wells from 3 biological replicates.Neutrophil extravasation kinetics through control (gray), ICAM‐1 KO (green), ICAM‐2 KO (magenta), and ICAM‐1/2 KO (orange) BOECs cultured on 3‐μm pore permeable filters. DiO‐stained neutrophils transmigrated towards C5a located in the lower compartment. Lines show means with 95% Cis of a total of 6 to 8 wells from 3 biological replicates.Basal leakage measured with Texas‐Red‐dextran extravasation kinetics through control (gray) and ICAM‐1 high sorted (green) HUVECs BOECs. Lines show means with 95% Cis of 3 wells from 3 biological replicates.Neutrophil extravasation kinetics through control (gray) and ICAM‐1 high sorted (green) HUVECs cultured on 3‐μm pore permeable filters. DiO‐stained neutrophils transmigrated towards C5a located in the lower compartment. Lines show means with 95% CIs of 3 wells from 3 biological replicates.

Using a Transwell leakage assay, we did not measure any basal leakage in any of the KO ECs when no neutrophils were present (Figs 5B and EV4A). When measuring permeability during neutrophil TEM under control conditions, we did not find any change in permeability. However, we did measure an increase in EC permeability when neutrophils crossed EC monolayers that were deficient for ICAM‐1 (Fig 5A and B). We also found an increase in permeability when neutrophils crossed the ICAM‐1/2 KO EC monolayers, whereas permeability was only slightly increased when neutrophils crossed ICAM‐2‐deficient ECs (Fig 5A and B). TEM of neutrophils through these monolayers was consistent with TEM under flow experiments (Figs 4A and EV4B). These data indicate that TEM hotspots functionally protect endothelial monolayer integrity from leakage during TEM.

To examine whether reducing the heterogeneous expression of *endogenous* ICAM‐1 also limited leakage during TEM, we sorted the top 5% ICAM‐1‐expressing ECs, referred to as ICAM‐1^high^ (Fig 5C). Indeed, the ICAM‐1^high^ EC population displayed lower heterogeneity compared with control ECs (Fig 5D). As the antibody used for cell sorting may interfere with neutrophil TEM, we tested its inhibitory properties. We observed no effect on adhesion, TEM, and neutrophil crawling dynamics when the antibody was incubated on the ECs for 24 h (Appendix Fig S7A–F), the same time the ECs are in contact with the antibody in the sorting experiments. Using TEM flow assays, we observed that ICAM‐1^high^ ECs showed increased, albeit nonsignificant, adhesion compared with control ECs (Fig 5E). Diapedesis efficacy was unaltered when comparing ICAM‐1^high^ with control ECs (Fig 5F). However, when quantifying adhesion and diapedesis hotspots, we found that neutrophils showed less clustered transmigration patterns on ICAM‐1^high^ ECs compared with control ECs (Fig 5G and H). In line with these results, permeability assays demonstrated that EC leakage upon neutrophil TEM was increased in homogenous ICAM‐1^high^‐sorted ECs, in which no hotspots were detected (Figs 5I and J, and EV4C and D). Thus, these data show that endogenous ICAM‐1 heterogeneity is responsible for establishing functional TEM hotspots in the endothelium that limit vascular leakage during TEM.

**Figure 6:**
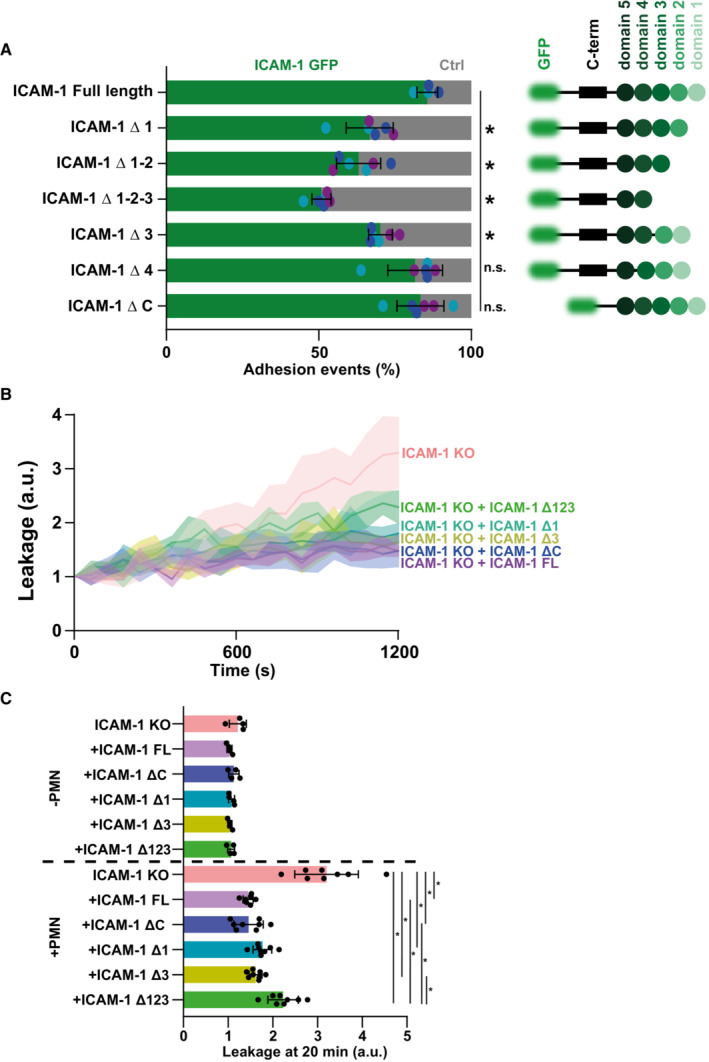
Integrin‐binding domains of ICAM‐1 important for adhesion hotspots, the intracellular domain is not Quantification of preference for neutrophils to adhere to ICAM‐1‐GFP truncations (green) expressed in ICAM‐1/2 KO BOECs or ICAM‐1/2 KO BOECs (Ctrl; light gray), treated overnight with TNFα. A schematic overview of all cloned ICAM‐1 truncations is displayed as well. Bars represent the percentage of neutrophils that adhere to indicated transfected ECs. Numbers are corrected for area occupied. Dots are percentages from individual timelapse images. Bars represent mean with standard deviation. Colors represent data from 6 videos from 3 biological replicates (2 videos from 2 biological replicates for the control). One‐way ANOVA (*n* = 4 for FL ICAM‐1, *n* = 6 for all others). With multiple comparison corrections, comparing all conditions to FL ICAM‐1. FL vs Δ 1: *P = 0.0004. FL vs Δ 12: *P < 0.0001. FL vs Δ 123: *P < 0.0001. FL vs Δ 3: *P < 0.0029. FL vs Δ 4: *P* = 0.5848. FL vs Δ C: *P* = 0.6014.Texas‐Red‐Dextran extravasation kinetics through ICAM‐1/2 KO (pink) BOECs, treated overnight with TNFα, with mosaicly expressed ICAM‐1‐GFP (purple), ICAM‐1‐GFP Δ123 (green), ICAM‐1‐GFP Δ1 (cyan), ICAM‐1‐GFP Δ3 (yellow) and ICAM‐1‐GFP ΔC (blue), cultured on 3‐μm pore filters. DiO‐stained neutrophils transmigrated towards C5a located in the lower compartment. Lines show means with 95% CIs of a total of 4 (without neutrophils) or 8 (with neutrophils) wells from 4 biological replicates.Quantification of Texas‐Red‐dextran extravasation kinetics through ICAM‐1/2 KO BOECs, treated overnight with TNFα, with mosaicly expressed ICAM‐1‐GFP, ICAM‐1‐GFP Δ123, ICAM‐1‐GFP Δ1, ICAM‐1‐GFP Δ3 and ICAM‐1‐GFP ΔC, after 20 min. Bar graphs represent mean and SD. One‐way ANOVA (*n* = 4 without PMNs, *n* = 8 with PMNs) with multiple comparison corrections was performed within both the conditions without and with neutrophils. Without neutrophils (ICAM‐1 KO vs FL: *P* = 0.1864, ICAM‐1 KO vs ΔC: *P* = 0.8335, ICAM‐1 KO vs Δ123: *P* = 0.3649, ICAM‐1 KO vs Δ1: *P* = 0.4300, ICAM‐1 KO vs Δ3: *P* = 0.2031, FL vs ΔC: *P* = 0.7616, FL vs Δ123: *P* = 0.9953, FL vs Δ1: *P* = 0.9975, FL vs Δ3: *P* > 0.9999, ΔC vs Δ123: *P* = 0.9570, ΔC vs Δ1: *P* = 0.9782, ΔC vs Δ3: *P* = 0.8222, Δ123 vs Δ1: *P* > 0.9999, Δ123 vs Δ3: *P* = 0.9987, Δ1 vs Δ3: *P* = 0.9952). With neutrophils (ICAM‐1 KO vs FL: *P < 0.0001, ICAM‐1 KO vs ΔC: *P < 0.0001, ICAM‐1 KO vs Δ123: *P < 0.0001, ICAM‐1 KO vs Δ1: *P < 0.0001, ICAM‐1 KO vs Δ3: *P < 0.0001, FL vs ΔC: *P* > 0.9999, FL vs Δ123: *P = 0.0014, FL vs Δ1: *P* = 0.5035, FL vs Δ3: *P* = 0.9375, ΔC vs Δ123: *P = 0.0017, ΔC vs Δ1: *P* = 0.5433, ΔC vs Δ3: *P* = 0.9532, Δ123 vs Δ1: *P* = 0.1420, Δ123 vs Δ3: *P* = 0.0199, Δ1 vs Δ3: *P* = 0.9600).

### The integrin‐binding domains of ICAM‐1 are required for functional TEM hotspots

Neutrophils use integrins LFA‐1 or Mac‐1 to bind to ICAM‐1 and ICAM‐2, both using different epitopes. To study in more detail which of these epitopes are crucial for establishing functional TEM hotspots, we generated deletion mutants of ICAM‐1, lacking one or more of the extracellular Ig‐like domains. All deletion mutants for ICAM‐1 were expressed in a heterogenous manner in ICAM‐1 KO ECs and stained, on nonpermeabilized samples, with an ICAM‐1 antibody directed against the first Ig‐like domain, showing normal distribution in apical filopodia (Appendix Fig S8). In accordance with earlier literature, ICAM‐1 lacking its intracellular domain (ΔC), did not localize to these apical filopodia anymore (Oh *et al*, 2007). To study which domains are crucial for TEM hotspot determination, we re‐expressed these truncations in ICAM‐1/2 KO ECs and found that the lack of Ig‐like domain 1 or 1–2 (Δ1/Δ1‐2). caused a mild decrease in TEM hotspots (Fig 6A). A similar decrease was observed when Ig‐like domain 3 (Δ3) was depleted (Fig 6A). Interestingly, no TEM hotspot preference was measured when the first 3 Ig‐like domains were deleted (Δ1‐2‐3; Fig 6A). Ig‐like domain 4, known for ICAM‐1 dimerization (Chen *et al*, 2007), had no effect on TEM hotspot preference (Δ4; Fig 6A). Interestingly, deletion of the intracellular tail of ICAM‐1, known to induce TEM‐mediated signals (Oh *et al*, 2007), did not influence hotspot recognition. These data showed that the first 3 Ig‐like domains, the LFA‐1 and Mac‐1 epitopes of ICAM‐1 are crucial for TEM hotspot determination, and that ICAM‐1 dimerization and intracellular signaling induced by the intracellular tail of ICAM‐1 are not involved.

**Figure EV5:**
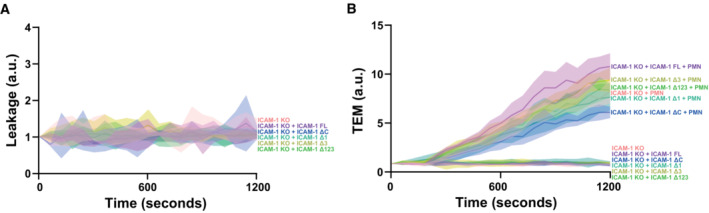
ICAM‐1 truncated construct overexpression in ICAM‐1 KO BOECs does not show basal leakage Basal leakage measured with Texas‐Red‐dextran extravasation kinetics through ICAM‐1 KO (pink) BOECs, treated overnight with TNFα, with mosaicly expressed ICAM‐1‐GFP (purple), ICAM‐1‐GFP Δ123 (green), ICAM‐1‐GFP Δ1 (cyan), ICAM‐1‐GFP Δ3 (yellow) and ICAM‐1‐GFP ΔC (blue). Lines show means with 95% CIs of 4 wells from 3 biological replicates.Neutrophil extravasation kinetics through ICAM‐1 KO (pink) BOECs with mosaicly expressed ICAM‐1‐GFP (purple), ICAM‐1‐GFP Δ123 (green), ICAM‐1‐GFP Δ1 (cyan), ICAM‐1‐GFP Δ3 (yellow) and ICAM‐1‐GFP ΔC (blue). cultured on 3‐μm pore permeable filters. DiO‐stained neutrophils transmigrated towards C5a located in the lower compartment. Lines show means with 95% CIs of a total of 4 (without neutrophils) or 8 (with neutrophils) wells from 3 biological replicates.

To study whether the observed increase in permeability in previous experiments is due to the loss of ICAM‐1‐mediated TEM hotspots, we measured permeability during TEM across ICAM‐1‐deficient EC monolayers that were rescued with truncated mutants. ICAM‐1‐KO ECs that expressed the ICAM‐1 mutant lacking the first 3 Ig‐like domains (Δ123) showed a significant increase in leakage compared with ICAM‐1‐FL rescue conditions (Fig 6B and C), whereas rescuing with truncated ICAM‐1 with just one of the two integrin‐binding domains was enough to prevent vascular leakage during TEM, indicating that the two integrin‐binding domains have an overlapping function. The total number of neutrophils that crossed the endothelial monolayers under these conditions was only marginally reduced (Fig EV5B). Note that the ICAM‐1 mutant that lacked the intracellular tail did not show any increase in permeability. However, the number of neutrophils that crossed this monolayer was reduced, in line with current literature that demonstrated a role of the intracellular domain of ICAM‐1 in leukocyte TEM (Appendix Fig S6B; Oh *et al*, 2007). None of the EC monolayers that expressed ICAM‐1 mutants showed any basal leakage in the absence of neutrophils (Fig EV5A). Taken together, these data suggest that the binding of leukocytic integrins to endothelial ICAM‐1 is required for TEM hotspot recognition. As a functional consequence, the existence of hotspots limits vascular permeability during TEM.

## Discussion

The existence of TEM hotspots has been recognized *in vivo* (Hyun *et al*, 2019), but there is no evidence for their biological relevance, nor is there a clear consensus on the mechanism for hotspot recognition by leukocytes (Grönloh *et al*, 2021). In this work, we use live‐imaging and newly developed computational methods for the analysis of hotspots, providing new insight that addresses these questions. Our work confirms that TEM hotspots can also be found *in vitro*. As for the physiological relevance, we show for the first time that these hotspots on the endothelial monolayer limit vascular leakage during TEM. Mechanistically, this study shows that the first 3 extracellular Ig‐domains of ICAM‐1 are crucial for hotspot recognition.

Neutrophil TEM hotspots were first described *in vivo*, where the involvement of LFA‐1 and Mac1 in different TEM phases was shown (Hyun *et al*, 2019). This study introduced the terms “Hotspot I” for transendothelial migration hotspots and “Hotspot II” for hotspots in the pericyte and basement membrane layer. In agreement with this study, we show that ICAM‐1, ligand for LFA‐1 and Mac‐1, is involved in “Hotspot I,” resulting in local TEM. Importantly, the hotspots described in our study are based only on the adhesion step of TEM, as we based our analysis on the spot the neutrophils initially adhere to the EC. Earlier research in our lab has demonstrated that after neutrophils adhere, they usually undergo diapedesis at the first junction they encounter (Arts *et al*, 2021). This means that, when ICAM‐1^high^ ECs act as a hotspot during the adhesion phase of TEM, most neutrophils will also undergo diapedesis at a junction of this same EC. Whether there is a next, “diapedesis hotspot” directly after the adhesion hotspot would be interesting to study. In fact, there is evidence that neutrophils prefer to transmigrate at tricellular junctions (Sumagin & Sarelius, 2010; Castro Dias *et al*, 2021), indicating that neutrophils definitely prefer certain junctions to undergo TEM and that this is also not a random process. This would be an attractive hypothesis regarding the prevention of local leakage during TEM, as this would mean that even fewer total junctions would be disrupted.

Our major finding lies in the fact that TEM hotspots function to limit vascular leakage during TEM. The endothelium uses the heterogeneous distribution of ICAM‐1 to induce TEM hotspots for leukocytes. The basis of TEM hotspots is the initial adhesion of the leukocytes on top of the endothelium, particularly driven by ICAM‐1. Depletion of ICAM‐1 does not hamper total TEM significantly but does increase vascular leakage during TEM. Previously, we showed that a F‐actin‐rich ring acts like an elastic strap around the perpetrating leukocyte to limit permeability during TEM (Heemskerk *et al*, 2016). This F‐actin ring is under tension as it needs the local activity of the small GTPase RhoA and downstream myosin activity. This previous work furthermore indicated a role for ICAM‐1 upstream from RhoA activation, albeit we were unable to directly link ICAM‐1 function to the formation of the F‐actin ring. We now find that ICAM‐1 is crucial in the formation of TEM hotspots and thereby reduces local permeability. However, the ICAM‐1 ΔC mutant shows that it is the disappearance of hotspots, and not the lack of downstream signaling towards the RhoA‐mediated pore closure, that causes vascular leakage. These data indicate that the formation of the F‐actin ring and the recognition of TEM hotspots through ICAM‐1 distribution are uncoupled. In addition, previous studies have demonstrated that truncating the intracellular domain of ICAM‐1, specifically the RKIKK domain, does only reduce diapedesis efficacy and does not impact total adhesion (Lyck *et al*, 2003; Oh *et al*, 2007).

It has been shown that leukocyte TEM does not decrease endothelial resistance and leakage in control conditions (Baluk *et al*, 1998; Zeng *et al*, 2002; Petri *et al*, 2011; Wessel *et al*, 2014; Heemskerk *et al*, 2016; Braun *et al*, 2020). Other groups have identified signaling pathways in the endothelium that lead to the tight closure of the endothelial gap that is induced by the penetrating leukocyte. Braun *et al* (2020) elegantly showed that platelet‐derived Ang1 activates the endothelial Tie2 receptor, resulting in local activation of the FGD5‐Cdc42 axis and closure of the gap (Braun *et al*, 2020). Martinelli *et al* (2013) have shown that local Rac1 activities are involved in the release of cellular tension signals that induce self‐restorative ventral lamellipodia to heal barrier micro‐wounds (Martinelli *et al*, 2013). These mechanisms may all be triggered when leukocytes penetrate the endothelial monolayer (van Buul, 2020; Grönloh *et al*, 2021). From a more efficient and energy‐saving cellular perspective, it makes sense to concentrate and minimize such signaling pathways to be able to keep the integrity of the vascular wall as good as possible.

We have compared neutrophil crawling dynamics around endothelial hotspot areas with nonhotspot areas. This revealed that neutrophils that used TEM hotspots showed much shorter crawling tracks. We hypothesize that these differences are due to higher ICAM‐1 expression that captures neutrophils on the spot more efficiently. However, as we do not see extended crawling tracks on ICAM‐1 KO ECs, it is likely that other factors are also involved, potentially regulated by leukocytes themselves. An interesting hypothesis to explore further involves the ability of neutrophils to leave behind “membrane trails” that support the migration of subsequent leukocytes (Lim *et al*, 2015).

Surprisingly, our results show that in ICAM‐1‐depleted EC monolayers, the effect on total adhesion and transmigration is minimal. Only when both ICAM‐1 and ‐2 are depleted, a clear decrease in neutrophil adhesion and therefore transmigration was observed, confirming earlier hypotheses that ICAM‐1 and ICAM‐2 have partly overlapping roles during TEM (Lyck & Enzmann, 2015). Previous work that studied the role of ICAM‐1 in neutrophil adhesion has primarily done this by blocking its ligands LFA‐1 and Mac‐1, and has observed significant decreases in leukocyte adhesion when either one or both these integrins were blocked (Smith *et al*, 1989; Ding *et al*, 1999). We hypothesize that our nonsignificant decrease in neutrophil adhesion on ICAM‐1 KO ECs can be explained by alternative LFA‐1/Mac‐1 binding partners present on ECs. ICAM‐2 is an obvious candidate, and we indeed found significantly reduced adhesion when both ICAMs were blocked. Besides ICAM‐1/2, junctional adhesion molecule A (JAM‐A) has been shown to be an LFA‐1 ligand (Ostermann *et al*, 2002). But importantly, our work adds new information to the separate roles of ICAM‐1 and ICAM‐2 in TEM. By overexpressing both adhesion molecules in a mosaic fashion, we show a preference for ICAM‐1 over ICAM‐2 for the adhesion of neutrophils. This preference may be a result of the reported higher binding affinity of ICAM‐1 with LFA‐1 (Li *et al*, 2018). Alternatively, ICAM‐1 is enriched in apical filopodia that extend into the lumen and thus readily accessible for the rolling leukocyte, which is not the case for ICAM‐2.

This study demonstrates that for hotspot formation, the first and third extracellular integrin‐binding Ig‐domains are required. ICAM‐1 has six isoforms, generated by alternative splicing (Ramos *et al*, 2014). All these isoforms contain the first Ig‐domain, while three of six isoforms lack the third Ig‐domain. In mice, LFA‐1 binds these isoforms with highly variable binding affinities (King *et al*, 1995), perhaps indicating that certain isoforms might be more important for hotspot recognition. In addition to isoforms, ICAM‐1 can undergo different post‐translational medications after TNFα treatment: a fully processed glycosylated form and a hypoglycosylated high‐mannose form, leading to reported differently functioning proteins (Scott *et al*, 2013). Exploring the abundance of each isoform in different types of ECs as a result of different inflammatory stimuli, and subsequently, their relevance for hotspots would be an interesting next step in this research.

In this study, we highlight the significance of the heterogeneous distribution of adhesion molecule protein expression within the endothelial monolayer for barrier integrity during TEM. In this study, HUVECs cultures were prepared from multiple umbilical cords, and donor variability could theoretically be an explanation for the observed heterogenous expression of adhesion molecules. However, the heterogeneous distribution of adhesion molecule such as ICAM‐1 upon inflammation is broadly recognized and found *in vivo* (Sumagin & Sarelius, 2006), and also observed in our patient samples. Remarkably, based on our results, other inflammation‐upregulated adhesion molecules VCAM‐1 and E‐selectin do not share the adhesion hotspot‐marking properties that ICAM‐1 does, suggesting a more intricate mechanism that may work differently for each protein. Earlier research has already provided clues at the epigenetic level: heterogeneity of well‐known endothelial protein von Willebrand factor (VWF) is dependent on noise‐induced changes in DNA methylation of the *VWF* promotor (Yuan *et al*, 2016), whereas VCAM‐1 mosaic expression is due to heterogenous states of *VCAM‐1* promotor methylation states (Turgeon *et al*, 2020).

Here, we have focused on the role of endothelial adhesion molecules in hotspot formation. In addition to adhesion molecules, chemokines are known to play an instrumental role during leukocyte TEM (Middleton *et al*, 2002; Shulman *et al*, 2011; Girbl *et al*, 2018). Therefore, it would be very interesting to measure possible heterogeneous expression patterns of chemokines including but not limited to CXCL1, CXCL2, CCL2, and IL‐8, especially in correlation with ICAM‐1 heterogeneity. In line with these studies, we found that chemokine presenting Atypical Chemokine Receptor 1 (ACKR1) is enriched on endothelial junctional membrane protrusions, which are actin‐based structures that neutrophils prefer as sites of diapedesis (Arts *et al*, 2021). This also indicates that indeed chemokines may play a role in the determination of hotspots.

In this study, we focused on neutrophils, as hotspots were first described for neutrophils *in vivo* (Hyun *et al*, 2019). However, extending these findings towards other leukocyte subsets would be highly relevant. For instance, because of their higher VLA‐1 expression levels, T‐cells are known to be more dependent on VCAM‐1 compared with neutrophils, which we found not to use VCAM‐1^high^ cells as hotspots. Perhaps this also means that T‐cells do not necessarily utilize the same hotspots as neutrophils, if they employ hotspots at all. Additionally, T‐cells generally use the transcellular route more so than neutrophils (van Steen *et al*, 2020), via a mechanism involving ICAM‐1 internalization and transcytosis towards the basal membrane via caveolae (Millán *et al*, 2006). Investigating whether ICAM‐1^high^ endothelial cells are better equipped for transcellular diapedesis compared with ICAM‐1^low^ cells would be an interesting next approach.

We did not perform mice experiments in our study to look at the role of ICAM‐1 heterogeneity in the context of hotspots or vascular permeability during TEM. To our knowledge, earlier studies using ICAM‐1 (−/−) mice have not reported, nor specifically focused on vessel leakage during neutrophil TEM (Sligh *et al*, 1993; Xu *et al*, 1994). Work with ICAM‐1 (−/−) mice in the context of T‐cell TEM over the blood–brain barrier did also not report increases in permeability during TEM (Lyck *et al*, 2003; Steiner *et al*, 2010), but looking into other vasculature in these mice would be interesting. To be able to compare our data to *in vivo* studies, neutrophil TEM experiments combined with vessel leakage experiments in tissues like the cremaster or peritoneum under inflammatory conditions would be required.

In conclusion, we have discovered how the endothelium takes advantage of adhesion molecule heterogeneous distribution within the endothelial monolayer by introducing TEM hotspots for leukocytes that function to limit vascular leakage during diapedesis and therefore maintain vascular integrity.

## Materials and Methods

### Plasmids

ICAM‐1‐GFP was described earlier (Kroon *et al*, 2018) and cloned into a lentiviral pLV backbone using SnaBI (ThermoFisher, FD0404) and XbaI (ThermoFisher, FD0684)/NheI (ThermoFisher, FD0973). To generate ICAM‐1 truncated sequences, Gibson cloning (NEB) was performed on the pLV‐ICAM‐1‐GFP plasmid. All constructs contain GFP as FP and the ICAM‐1 signal peptide, consisting of amino acids Met1 to Ala27. ICAM‐1 Δ 1 is truncated from Gln28 to Val109; ICAM‐1 Δ 12 has a deletion from Gln28 to Phe212; ICAM‐1 Δ 123 lacks Gln28 to Ile307; ICAM‐1 Δ3 is truncated from Val213 until Ile307; ICAM‐1 Δ 4 lacks Pro311 to Arg391; ICAM‐1 Δ C terminates at Asn504. pLV‐ICAM‐2‐mKate was constructed and packaged by VectorBuilder (Vector ID is VB200624‐1164vtm). pLV‐mNeonGreen‐Caax and pLV‐mScarletI‐CAAX have been described earlier by us (Arts *et al*, 2021). mEos4b‐N1 was a gift from Micheal Davidson (Addgene # 54814; http://n2t.net/addgene:54814; RRID:Addgene_54814; Paez‐Segala *et al*, 2015). mEos4b was PCRed out of the mEos4b‐N1 vector, after which it was ligated into a pLV backbone using SnaBI and XbaI/NheI. All primers used in cloning are shown in Appendix Table S1.

### Antibodies

Alexa Fluor 647‐conjugated ICAM‐1 mouse monoclonal antibody was purchased from AbD Serotec (MCA1615A647T; IF and FACS 1:400). Alexa Fluor 546‐conjugated ICAM‐1 mouse monoclonal antibody was bought from Santa Cruz (sc‐107 AF546; IF 1:400 Vessel‐on‐a‐chip 1:200 whole‐mount stain 1:100). FITC‐conjugated ICAM‐1 mouse monoclonal antibody was purchased from R&D (BBA20; FACS 1:100). ICAM‐1 rabbit polyclonal antibody was purchased from Santa Cruz (SC‐7891; WB 1:1,000). ICAM‐2 monoclonal mouse antibody was purchased from Invitrogen (14‐1029‐82; IF 1:200). PE‐conjugated ICAM‐2 monoclonal mouse antibody was bought from BD (558080; FACS 1:200). ICAM‐2 rabbit monoclonal antibody was bought from Invitrogen (MA5029335; WB 1:500). VCAM‐1 monoclonal mouse antibody was purchased from Merck (MAB2511). E‐selectin polyclonal goat antibody was bought from R&D (BBA18; IF 1:100). Alexa Fluor 647‐conjugated PECAM‐1 monoclonal mouse antibody was bought from BD (561654; whole‐mount stain 1:200). Alexa Fluor 647‐conjugated VE‐cadherin mouse antibody was purchased from BD (561567; Vessel‐on‐a‐chip 1:200). Alexa Fluor 488‐conjugated polyclonal chicken anti‐mouse antibody (A21200; IF 1:200) and Alexa Fluor 647‐conjugated polyclonal chicken anti‐mouse antibody (A21463; IF 1:200) were purchased from Invitrogen. Alexa Fluor 647‐conjugated polyclonal chicken anti‐goat antibody was purchased from Invitrogen (A‐21469; IF 1:200). Alexa Fluor 488 phalloidin was purchased from Invitrogen (whole‐mount stain 1:200). Hoechst 33342 (IF, vessel‐on‐a‐chip and whole‐mount stain 1:50,000) was purchased from Molecular Probes (H‐1399). Mouse monoclonal actin antibody for western blot (1:2,500) was purchased from Sigma (A3853). Donkey anti‐rabbit IRDye 800 (926‐32213; WB 1:5,000) and donkey anti‐mouse 680 (926‐68022; WB 1:5,000) were purchased from LI‐Cor. All antibodies were used according to the manufacturer's protocol.

### Cell culture and treatments

HUVEC were purchased from Lonza (C2519A) and cultured on fibronectin (FN)‐coated dishes in Endothelial Growth Medium 2 (EGM‐2) supplemented with SingleQuots (Promocell, C‐22011) and 100 U/ml penicillin and streptomycin (P/S) at 37°C in 5% CO_2_. HUVEC were cultured up to passage 7 and never allowed to grow above 70% confluency before the start of an experiment. Blood outgrowth endothelial cells (BOEC) were isolated from umbilical cord blood according to this protocol (Martin‐Ramirez *et al*, 2012). BOEC were grown on 0.1% gelatin‐coated dishes during outgrowth and during experiments in EGM‐2 supplemented with SingleQuots, 100 U ml^−1^ P/S and 18% fetal calf serum (Bodinco, Alkmaar, The Netherlands) at 37°C in 5% CO_2_. HUVECs and BOECs were inflamed with 10 ng/ml recombinant TNFα (Peprotech, 300‐01A), 10 ng/ml IL‐1β (Peprotech, 200‐01B), 0.5 ng/ml IFN‐γ (R&D, 285‐IF‐100), or 10 ng/ml LPS (Sigma, L2880) 20 h before an experiment.

HEK‐293T (ATCC) was cultured in Dulbecco's Modified Eagle Medium (DMEM; Gibco, 41965‐039) containing 10% fetal calf serum, 100 U/ml P/S. By transfection of the third‐generation lentiviral packaging plasmids with TransIT (Myrus, Madison, WI, USA) according to the manufacturer's protocol, lentiviral particles containing pLV plasmids were generated. On the second and third days after transfection, lentivirus‐containing supernatant was harvested, filtered (0.45 micron), and concentrated with Lenti‐X concentrator (Clontech, 631232). Virus was added to HUVEC or BOECs 1:250 to 1:500, depending on the efficacy of the virus. In case all cells were required to express the plasmid, a 2‐day 1.5 μg/ml puromycin (InvivoGen, ant‐pr‐1) selection was performed. Endothelial cells were used in assays at least 72 h after initial transduction. All cells used in the manuscript were tested for mycoplasma contamination every 3 months.

### Generating ICAM knockout BOEC

ICAM‐1 and ICAM‐2 knockout BOEC were generated using guide RNAs (gRNAs) GCTATTCAAACTGCCCTGAT (ICAM‐1) and GAGGTATTCGAGGTACACGTG (ICAM‐2) that were ligated into a lentiviral Crispr vector (LentiCRISPRv2) containing Cas9 that was digested with BsmBI (ThermoFisher, FD0454) gRNA were designed using Crispr (Concordet & Haeussler, 2018). For ICAM‐1, we targeted exon 2. For ICAM‐2, we targeted exon 4. As a negative control, the CRISPR vector without a gRNA was used. Virus was produced in HEK‐293T cells and transductions were performed in cord blood BOECs. Transduced cells were selected using 1.5 μg/ml puromycin for 2 days, after which cells were single cell sorted into 96‐well plates coated with 0.1% gelatin with a BD FACS Aria™ III Cell Sorted (BD). For ICAM‐2 knockout and for the ICAM‐1/2 double knockout, ICAM‐2 negative cells were sorted. For control gRNA, single cells positive for ICAM‐2 were sorted. Since ICAM‐1 only gets expressed in inflammatory conditions, and BOECs stop growing after receiving inflammatory treatments, it was not possible to sort ICAM‐1 knockout candidates with a fluorescent selection. Single cells were sorted and all monoclonal populations were tested for ICAM‐1 expression when they reached around 100,000 cells, after which only the ICAM‐1 lacking cell lines were kept in culture. Correct knockouts were tested by Western blot and genomic DNA extraction followed by sequencing using a DNeasy Blood & Tissue Kit (Qiagen, 69504; Appendix Fig S2).

### Neutrophil isolation

Polymorphonuclear neutrophils were isolated from whole blood and extracted from healthy voluntary donors that signed informed consent according to the rules maintained by the Sanquin Medical Ethical Committee, which are based on rules and legislation in place within The Netherlands. The rules and legislations were based on the Declaration of Helsinki (informed consent for participation of human subjects in medical and scientific research) and guidelines for Good Clinical Practice. Blood was always processed within 2 h after donation. Whole blood is diluted 1:1 with 5% TNC in phosphate buffering solution (PBS; Fresenius Kabi, Zeist, The Netherlands) and pipetted on 12.5 ml Percoll (1.076 g/ml). Next, a 20‐min centrifugation (Rotina 420R) at 800 *g* with a slow start and no brake was performed on the diluted blood. After discarding the monocyte‐ and lymphocyte‐containing ring fraction, 45 ml ice‐cold erythrocyte lysis buffer (155 mM NH_4_Cl, 10 mM KHCO_3_, 0.1 mM EDTA, pH7.4 in Milli‐Q [Gibco, A1283‐01]) was added to the pallet to lyse erythrocytes for 15 min. Erythrocyte lysis was performed twice, with a centrifuge step at 500 *g* for 5 min at 4°C in between. Neutrophils were then centrifuged again at 500 *g* for 5 min at 4°C, washed once with 30 ml ice‐cold PBS, centrifuged again at 500 *g* for 5 min at 4°C and resuspended in RT HEPES medium (20 mM HEPES, 132 mM NaCl, 6 mM KCl, 1 mM CaCl_2_, 1 mM MgSO_4_, 1.2 mM K_2_HPO_4_, 5 mM glucose (All Sigma‐Aldrich), and 0.4% (w/v) human serum albumin (Sanquin Reagents), pH7.4. Neutrophil counts were determined using a cell counter (Casey)). Neutrophils were kept at a concentration of 2 million/ml at RT. Neutrophils were kept no longer than 4 h after isolation.

### Neutrophil transmigration under physiological flow

A 30,000 HUVECs or 20,000 BOECs per lane were seeded in, respectively, FN‐ or collagen‐coated Ibidi μ‐slides VI^0.4^ (Ibidi, Munich, Germany) and grown for 48 h. TNFα treatment (10 ng/ml) was performed 20 h before the experiment when the endothelial cells were grown into a confluent monolayer. A total of 6 million neutrophils, at 2 million/ml, were membrane‐labeled for 20 min at 37°C with Vybrant™ DiO or DiD Cell‐labeling solution (1:6,000). Stained neutrophils were centrifuged for 3 min at 300 *g* at RT to wash away residual labeling solution. Neutrophils were resuspended in HEPES medium to a concentration of 1 million/ml. After letting the neutrophils recover at RT for 20 min, 1 million neutrophils at a time were incubated at 37°C for 20 min before using them. The Ibidi flow chamber containing the endothelial cells was connected to a perfusion system and underwent a shear flow of 0.5 ml/min (0.8 dyne/cm^2^) for 2 min before injecting 700,000 neutrophils into the tubing system. During neutrophil flow experiments, the order of samples was changed up each time to minimize the effect of neutrophil freshness on results.

Except for experiments with photoconvertible proteins, flow assays were imaged using an Axiovert 200 M widefield microscope, using a 10× NA 0.30 DIC Air objective (Zeiss). Fluorescent excitation light was provided by an HXP 120 C light source at 100% intensity and a TL Halogen Lamp at 6.06V for transmitted light. Signal was detected with an AxioCam ICc 3 (Zeiss) camera. For the DIC channel, an exposure of 32 ms was used. For DiO‐stained neutrophils, a 450–490 excitation filter, a 495 beam splitter, and a 500–550 emission filter were used with an exposure of 1,900 ms. For DiD‐stained neutrophils, a 625–655 excitation filter, a 660 beam splitter, and a 665–715 emission filter were used with an exposure of 1,400 ms. To analyze neutrophil crawling dynamics and diapedesis locations Images were taken every 5 s for 15 min in two positions in the middle of the ibidi flow chamber lane. Immediately after acquiring the time lapse, a tile scan of 4 × 6 frames was collected to quantify total adhesion and transmigration numbers. Images were taken using Zeiss using Zen Blue software. The tile scan was stitched using Zen Black software, using the DIC channel for stitching.

### Static neutrophil adhesion assay

A 35,000 BOECs were seeded onto a 25‐mm FN‐coated coverslip, after which the ECs were cultured for 2 days until a monolayer was grown. ECs were treated with TNFα 24 h before imaging. 100,000 neutrophils were added to the monolayer without flow conditions and were incubated together for 5 min. Then, images were made with an Axiovert 200 M widefield microscope, using the DIC channel, as described above.

### Quantification of neutrophil transmigration dynamics

All analyses were performed in Imaris version 9.7.2. To quantify total adhesion and diapedesis efficacy, a spot analysis was performed on the tile scans to count cells adhering on top of the monolayer and cells crawling on the subendothelial side of the monolayer. Spot analysis was performed on the DiD or DiO channel, with an estimated dot size of 8 microns. Spots were manually thresholded based on the spot quality filter in Imaris to discriminate neutrophils from background signal. To distinguish neutrophils above and underneath the endothelium, a filter based on intensity in the DIC channel was added to the pipeline. Since neutrophils are white and round when on top of the endothelium and black and spread out when underneath the endothelium (Fig 1A), this filter could be used to separately count adhering and transmigrated neutrophils. Total adhesion was calculated as # adhering neutrophils + # transmigrated neutrophils, and due to large donor‐dependent variation was normalized to a control experiment and shown as a percentage. Neutrophil diapedesis efficacy was quantified as (# adhering neutrophils/# total detected neutrophils) * 100%. The same spot analysis on neutrophils above the endothelial layer was performed on timelapse data to quantify neutrophil crawling dynamics. A tracking step was added to the pipeline to connect the spots of each frame and detect neutrophil crawling patterns. For tracking analysis, the auto‐regressive motion was used, with a maximum distance of 20 μm between spots and allowing a gap size of 1 frame. Finally, tracks with less than 4 spots were filtered out to remove rolling neutrophils from the dataset. For optimal results, no more than 200 neutrophil tracks were allowed per video, and tracks were all manually checked for correctness. From this analysis, crawling speed, length, displacement, duration, and linearity were calculated.

### Quantification of hotspot dynamics

Analysis of neutrophil crawling tracks in Imaris software was performed in widefield timelapse data to compare the behavior of neutrophils at hotspots with neutrophils not utilizing hotspots. Only tracks that ended with diapedesis were used in this analysis. Tracks were classified as “hotspot tracks” when they ended within 50 microns, the average diameter of a HUVEC cell, of another ending track. Neutrophils were classified as a “nonhotspot track” if this criterium was not met. To assess the randomness of neutrophil adhesion sites, track analysis was performed in Imaris on crawling neutrophils. All last spots of endothelial crawling tracks were classified as “adhesion sites.” All adhesion site locations were masked in a new frame and a time projection was performed to generate one frame with all adhesion sites. A spot analysis in Imaris was done on this frame to count the number of adhesion sites in the time lapse and the mean distance to its one, three, five, or nine nearest neighboring adhesion sites was calculated. In FIJI (v1.52p; Schindelin *et al*, 2012), the same number of random spots was generated in an image with the same dimension size as the time‐cropped timelapse frame. The same spot analysis and subsequent calculations were performed on this image. To create a parameter for randomness, the median distance to *n* nearest neighbors of adhesion sites (meaning the first spot of each neutrophil crawling track) was divided by the median distance to *n* nearest neighbors of random sites. The higher this value, the more the adhesion sites approach a purely random distributed pattern. Only tracks that ended in successful diapedesis were used in this analysis.

### Artificial hotspot neutrophil flow assay

To generate artificial adhesion molecule heterogeneity, ICAM‐1/2 double knockout BOECs were transduced with (truncated) variants of ICAM‐1 and ICAM‐2. No puromycin selection was performed to preserve the nontransduced cells. Neutrophil flow assays were performed as described above, using unstained neutrophils. The DIC channel was imaged the same as described above. GFP was imaged with the same settings as DiO. mKate was imaged using a 559–585 excitation filter, a 590 beam splitter, and a 600–690 emission filter, with an exposure time of 1,200 ms. To quantify whether neutrophils preferred to adhere to transduced cells, neutrophil landing spots were manually analyzed, tallying whether a neutrophil that later underwent successful diapedesis adhered to a transduced or nontransduced cell. To take into account the variation in transduction efficacy between fields of view, the counted adhesion events were normalized against the percentage of the area in the field of view that was covered by transduced cells.

### Fixed immunofluorescent stains

For regular 2D cultured samples, HUVECs or BOECs were cultured in, respectively, FN‐ and collagen‐coated Ibidi μ‐slides VI^0.4^ (Ibidi, Munich, Germany). For fixation, 100 μl 4% Paraformaldehyde (PFA) in PBS++ (PBS containing 0.5 MgCl_2_ and 1M CaCl_2_) was added to a drained flow chamber. Since all antibodies bound to extracellular epitopes, no permeabilization step was performed. Samples were blocked with 2% Bovine Serum Albumin (BSA) in PBS++. Primary antibodies were incubated for 1 h at RT in PBS++, after which, if not working with directly conjugated antibodies, secondary antibodies were also incubated for 1 h at RT. Between all fixation, blocking, and staining steps, the flow chamber was washed three times with PBS++. If two primary antibodies were both raised in the same species, a three‐step staining was performed: starting with an unconjugated primary antibody, followed by an accompanying secondary antibody, followed by second, directly conjugated antibody.

A Zeiss LSM 980 with Airyscan 2 module was used for detailed high‐resolution confocal imaging of fixed samples, using a Plan‐Apochromat 40× NA 1.3 oil DIC objective (Zeiss, 420762‐9800‐000) and a voxel size of 0.053 × 0.053 × 0.220 μm to capture Z‐stacks. For all images, Multiplex SR‐8Y settings were used and a GaAsP‐PMT detector was used as a detector. GFP was excited using a 488 nm laser with a laser power of 0.2%, mKate was imaged using a 561 nm laser with 2.4% laser power, and Alexa Fluor 647 was excited with a 639 nm laser using 0.6% laser power. Images were acquired and 3D Airyscan‐processed in Zen Blue version 3.3. Maximum projections were constructed in FIJI. Vessel‐on‐a‐chip samples were cultured and imaged as described here (Van Steen *et al*, 2021).

### Confocal imaging of adhesion molecule heterogeneity

To image the heterogeneity of adhesion molecules, fixed Ibidi flow chambers were stained for nuclei and ICAM‐1, ICAM‐2, or VCAM‐1. Z‐stack imaging was performed with the Zeiss LSM 980, using its confocal mode, using a Plan‐Apochromat 20x objective NA 0.8 (Zeiss, 420650‐9903‐000). Voxel size was 1.184 × 1.184 ×0.5 μm. Hoechst was imaged with a 405 nm laser at 5.50% laser power and Alexa Fluor was excited with a 639 nm laser with 8% laser power. To measure heterogeneity, FIJI was used to generate sum projections of the Z‐stacks. A rolling ball background subtraction of 25 pixels was performed on the nuclei channel, after which the nuclei were segmented by a threshold and a particle analysis on particles between 50–1,000 pixels was performed. Then, fluorescent intensity was measured in the ICAM‐1/ICAM‐2/VCAM‐1 channel. Data were normalized within every field of view to correct for inherent differences between fields of view. Coefficient of variation (SD/mean) of fluorescent intensity was used as a measurement of heterogeneity in the dataset.

For subconfluent EC experiments, 1.500 (1:20^th^ of the amount for a confluent monolayer), HUVEC were seeded into an Ibidi flow chamber. Cells were then treated with TNFα the next day and were fixed and stained 2 days after seeding. To image enough cells for heterogeneity quantification, tile scans of 5 × 5 field of views with the same settings as above were imaged. Data analysis was performed in the same way as on confluent monolayers, with only an added image stitching step in Zeiss Zen blue software being added before making sum projections in FIJI.

### Patient sample confocal imaging

Patient tissue samples were received from the department of Pathology of the Amsterdam UMC, location AMC. All tissue samples were obtained with informed consent and according to Dutch guidelines for secondary used biological materials. Patient tissue samples were received from the department of Pathology of the Amsterdam UMC, location AMC. All samples were obtained and handled according to current Dutch legislation regarding the responsible secondary use of human tissues. Chronically inflamed patient samples, originating from the colonic mesentery, were obtained from inflammatory bowel disease patients, were obtained from inflammatory bowel disease patients during partial colectomy. Healthy mesenteric tissue was obtained from residual tissue of patients with intestinal carcinoma undergoing resection surgery. All tissue was stored in PBS++ at 4 degrees and prepared for imaging within 24 h. Samples were prepared by cutting off small pieces of around 0.5 cm in diameter. If required, samples were incubated for 4 h in PBS++ containing 10 ng/ml TNFα for 4 h at 37°C. These pieces were fixed with 4% PFA for 15 min at 37°C, permeabilized for 10 min with 0.5% triton‐X at RT, and finally blocked with 2% BSA for 30 min at RT. Between all steps, the sample was washed with PBS++. Stains were performed with a 1‐h incubation step by putting the sample in an antibody solution in a 1.5 ml Eppendorf in a slow rotator at 37°C. Finally, tissue samples were mounted on glass bottom microwell dishes (MatTek, P35G‐1.5‐14‐C) using 10% Mowiol.

Samples were imaged with the Zeiss LSM 980 with Airyscan module, using the same set‐up as described above and having a voxel size of 0.038 × 0.038 × 0.170 μm. Hoechst was measured using a 405 nm laser with 2.4% laser power, ATTO‐488 was measured with a 488 nm laser with 0.5% laser power, Alexa Fluor 568 was excited with a 561 nm laser with 4.5% laser power, and Alexa Fluor 647 was excited using a 639 nm laser with 8.5% laser power. All images were 3D Airyscan‐processed.

To measure the heterogeneity of ICAM‐1 in the patient samples, sum projections were made of the imaged Z‐stacks. Then, manually regions of interest for each individual EC were drawn based on the PECAM‐1 stain. These drawn regions were then projected on the ICAM‐1 channel, and the mean intensity of each EC was measured. For heterogeneity measurements, data were normalized within every field of view to correct for inherent differences between fields of view. The coefficient of variation (SD/mean) of fluorescent intensity was used as a measurement of heterogeneity in the dataset.

### mEos4b photoconverting assay

For photoconversion assays, HUVECs were transduced and puromycin‐selected with mEos4b. Neutrophil flow assays with unstained neutrophils were performed as described above. Imaging was performed on the Zeiss LSM 980, using its confocal mode as described above. First, neutrophil TEM was live‐imaged for 15 min at three positions, imaging only the transmitted light channel. Afterward, the same three positions were imaged fluorescently in channels designed to capture both the green and red emitting variants of mEos4b. For the green channel, a 488 nm laser with 4% laser power was used. For the red channel, a 561 nm laser with 5% laser power was used. Frames were captured every 8 s, and after the fourth frame, the interactive bleaching module was utilized to photoconvert mEos4b in the whole field of view towards its red emitting variant. For photoconverting, the 405 nm laser was used at 50% for 5 s. After photoconversion, the Ibidi flow chambers were immediately fixed and stained as described above for nuclei and an adhesion molecule. To find back exactly the same fields of view that were live‐imaged, we scanned the Ibidi flow chamber for photoconverted mEos4b and took images to measure the heterogeneity of adhesion molecules. To correlate adhesion events to the level of adhesion molecule expression, all ECs were numbered, after which the number of adhesion events followed by either diapedesis or detachment on each EC was tallied. Finally, all ECs on which adhesion events have taken place were ranked from 0% (lowest expression) to 100% (highest expression).

### Texas‐Red‐Dextran permeability and transmigration assay

Endothelial cells (50,000 HUVEC or 35,000 BOEC) were seeded in FN‐coated 24‐well cell culture inserts (Corning FluoroBlok, Falcon, 3.0‐μm pore size 351151) in a 24‐well plate (Corning Companion Plate, Falcon, 353504) and cultured for 48 h. Endothelial cells were treated with 10 ng/ml TNFα 20 h before the experiment. 100,000 DiO labeled neutrophils (1:6,000) and 100 μg Texas‐Red‐Dextran (70 kDa; Sigma) in HEPES medium (20 mM HEPES, 132 mM NaCl, 6 mM KCl, 1 mM CaCl_2_, 1 mM MgSO_4_, 1.2 mM K_2_HPO_4_, 5 mM glucose (All Sigma‐Aldrich), and 0.4% (w/v) human serum albumin (Sanquin Reagents, Amsterdam, The Netherlands), pH7.4) were added to the upper compartment of the culture insert in a total volume of 120 μl. 0.1 nM C5a (Sigma C‐5788) in HEPES medium was added to the bottom compartment in a total volume of 600 μl. Leakage and neutrophil TEM were measured simultaneously for 20 min with an interval of 1 min using an Infinite F200 pro‐plate reader (TECAN) at 37°C. DiO‐labeled neutrophil TEM dynamics were measured using EX BP 490/9 and EM BP 535/20. Leakage dynamics of Texas‐Red‐Dextran were measured with EX BP 595/9 and EM BP 630/20. To measure basal leakage, just Texas‐Red‐dextran was added to the upper compartment.

### Western blotting

BOECs were grown in collagen‐coated 6‐well culture plates and washed twice with PBS++. Lysis was performed with NP40 lysis buffer (50 mM Tris–HCl, 100 mM NaCl, 10 mM MgCl_2_, 1% NP40, and 10% glycerol, pH7.4) with 1:500 protease inhibitor. Protein samples were centrifuged at 14,000 *g* at RT for 10 min and resuspended in an SDS sample buffer containing 4% β‐mercaptoethanol. Samples were boiled at 95°C for 3 min to denature proteins and separated on a 4–12% NuPage Bis–Tris gel (Invitrogen, NP0322BOX). Proteins were transferred using an iBlot Gel Transfer device (Invitrogen) for 7 min to a nitrocellulose membrane (Invitrogen, IB301002). Membranes were subsequently blocked with a 5% milk solution in tris‐buffered saline with Tween 20 (TBST) at RT for 30 min. Primary antibodies were incubated overnight at 4 degrees in TBST and secondary IRDye 800 and IRDye 680 antibodies were incubated at RT for 1 h. After each blocking and staining step, the membranes were washed with TBST 3× min. Western blots were developed using an Odyssey imaging system.

### Cell sorting ICAM‐1 high cells

HUVECs were grown into confluent monolayers in a total of 3 t150 flasks and incubated overnight with 10 ng/ml TNFα. After two washing steps with PBS++, HUVECs were stained for ICAM‐1. Antibody stains for ICAM‐1 were performed before cell detachment to mimic the ICAM‐1 heterogeneity observed in monolayers most optimally. ICAM‐1 antibody was incubated with the monolater for 20 min in EGM‐2 at 37°C. After two more washing steps with PBS++, 5 ml accutase (Sigma‐Aldrich, A6964) was added to each t150 for 7 min at RT to take cells into suspension. Next, 5 ml EGM‐2 was added to the suspension, and cells were spinned down at 200 *g* for 5 min. The cell pellets were resuspended in EGM‐2 in polypropylene tubes. A FACSAria™ II or III Cell Sorter (BD) was used to sort the ICAM‐1 5% high cells into suspension. As a control, the whole living cell population was sorted. After sorting, cells were seeded into Ibidi flow chambers or on Transwell inserts, and neutrophil flow experiments and dextran leakage assays were conducted as described above.

### Statistics

Data are presented as either means or medians + SD, indicated for each graph. For neutrophil quantifications, comparisons between two groups were performed by a paired *t*‐test, and comparisons between multiple groups were performed by one‐way paired ANOVAs, pairing data of a single donor. For other experiments with two conditions, a student *t*‐test or Mann–Whitney test was performed. For other experiments with multiple conditions, a one‐way ANOVA was performed, indicating which conditions were compared. For calculation correlations, Pearson *r* was calculated. A two‐tailed *P* value of < 0.05 was considered significant. For microscopy images, representative images are shown. No blinding procedures were taken during experiments. For Transwell experiments, continuous data were plotted with a 95% confidence interval, using the PlotTwist web app (Goedhart, 2020).

## Author contributions

**Max LB Grönloh:** Conceptualization; formal analysis; investigation; visualization; methodology; writing—original draft; writing—review and editing. **Janine Arts:** Conceptualization; formal analysis; investigation; visualization; methodology. **Sebastián Palacios Martínez:** Investigation. **Amerens A van der Veen:** Investigation. **Lanette Kempers:** Methodology. **Abraham CI van Steen:** Investigation; methodology. **Joris JTH Roelofs:** Resources. **Martijn A Nolte:** Conceptualization; writing—review and editing. **Joachim Goedhart:** Conceptualization; supervision. **Jaap D van Buul:** Conceptualization; resources; data curation; supervision; funding acquisition; writing—original draft; project administration; writing—review and editing.

## Disclosure and competing interests statement

The authors declare that they have no conflict of interest.

## Supporting information



Appendix

Expanded View Figures PDF

PDF+

## Data Availability

This study includes no data deposited in external repositories.
