# Supplementary material for: Endothelial transmigration hotspots limit vascular leakage through heterogeneous expression of ICAM‐1
Source: EMBO Rep. 2022 Nov 16;24(1):e55483. doi: 10.15252/embr.202255483 (PMC9827561; doi:10.15252/embr.202255483)
Supplement: Supplementary file 1 — Appendix [file EMBR-24-e55483-s002.pdf]

Appendix

Table of content

1. Appendix Figure S1 p.2
2. Appendix Figure S2 p.3
3. Appendix Figure S3 p.4
4. Appendix Figure S4 p.5
5. Appendix Figure S5 p.6
6. Appendix Figure S6 p.7
7. Appendix Figure S7 p.8
8. Appendix Figure S8 p.9
9. Appendix Table S1 p.10

Appendix Figure S1

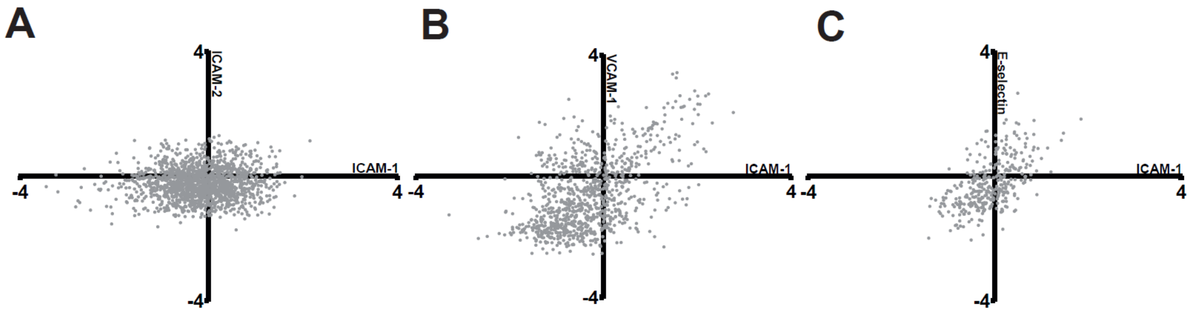

Appendix Figure S1. *Adhesion molecule protein expression correlation with ICAM-1.* **(A)** Correlation plot of Log2-transformed fluorescent intensity of ICAM-1 and ICAM-2, normalized within each field of view. r=0.04481, p=0.072 **(B)** Correlation plot of Log2-transformed fluorescent intensity of ICAM-1 and VCAM-2, normalized within each field of view. r=0.532, p<0.001**.** **(C)** Correlation plot of Log2-transformed fluorescent intensity of ICAM-1 and E-selectin, normalized within each field of view. r=0.577, p<0.001**.**

Appendix Figure S2

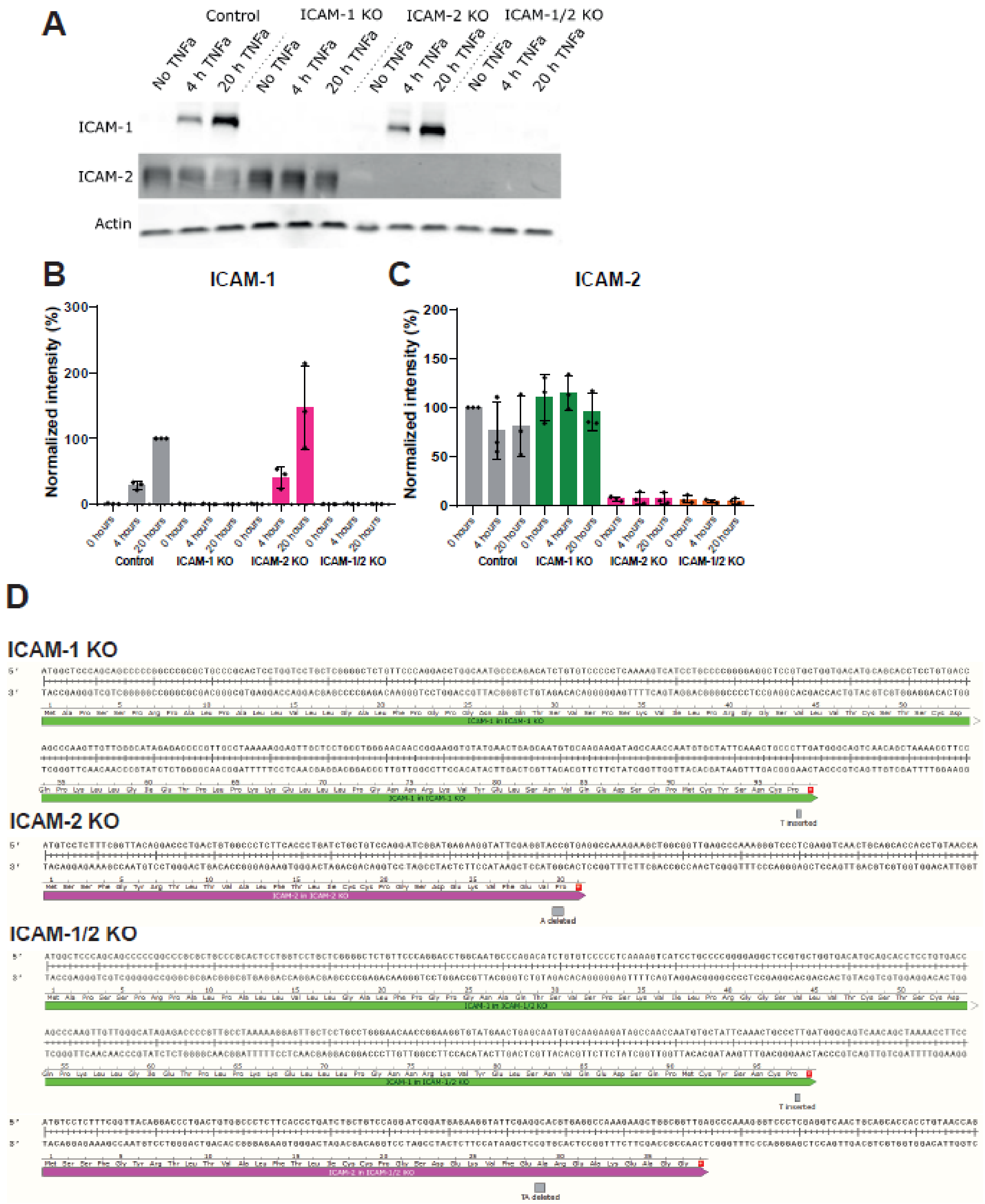

Appendix Figure S2. *Verification of ICAM-1/2 knockout BOECs.* **(A)** Western blot for ICAM-1 and ICAM-2 in Crispr knockouts after 0, 4 and 20 hours TNF-α. One representative gel out of three performed gels is shown. Actin is used as loading control. **(B,C)** Quantification of western blot for ICAM-1 **(B)** and ICAM-2 **(C)**, normalized to the actin loading control. Bar graphs display mean with SD. ICAM-1 data is normalized to 20 hours TNF-α in control BOECs, ICAM-2 data is normalized to 0 hours TNF-α in control BOECs. **(D)** Sequencing results of knockout cells. Mutations are depicted in grey block. Premature stop codon is depicted as star in red box. The protein sequences of the truncated ICAM-1 and ICAM-2 that are brought to expression in the KO BOECs are shown above the nucleotide sequence.

Appendix Figure S3

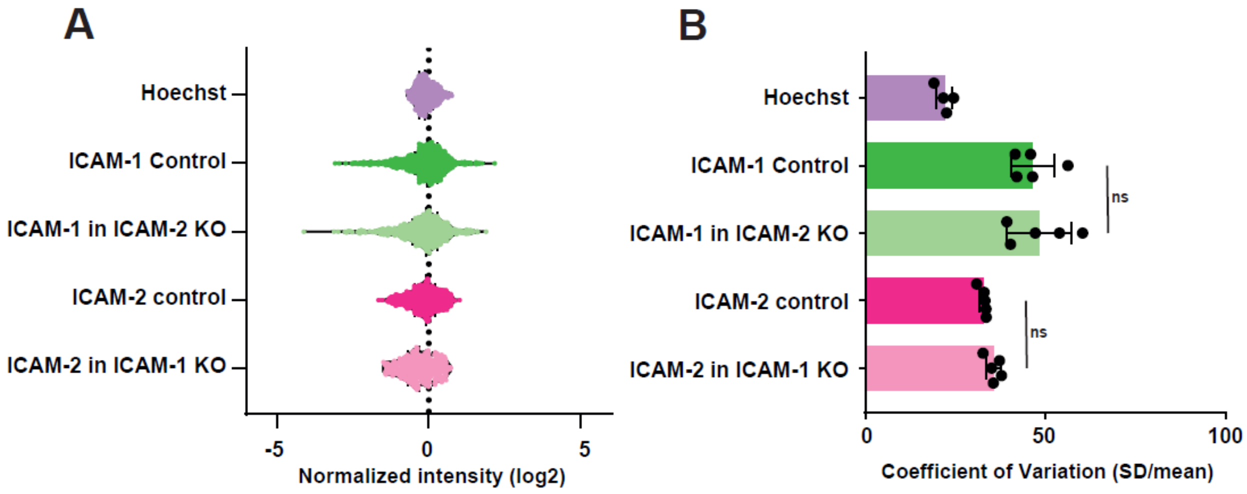

Appendix Figure S3. *ICAM-1 and ICAM-2 knockout do not influence each other's heterogeneity.* **(A)** Violin plots displaying Log2-normalized fluorescent intensity of Hoechst in control (n = 488 cells), ICAM-1 in control (n = 611 cells) and in ICAM-2 KO BOECs (n = 497 cells), and ICAM-2 in control (n = 808 cells) and in ICAM-1 KO BOECs (n = 336 cells), treated overnight with TNFα. Data is from 2 independent experiments **(B)** Bar graph displaying coefficients of variation (CoV) of all field of views measured in figure S4A. Mann-Whitney test (n=4 for Hoechst, n=5 for orhers) between ICAM-1 stain conditions ($p > 0.9999$) and ICAM-2 stain condition ($p = 0.0952$).

Appendix Figure S4

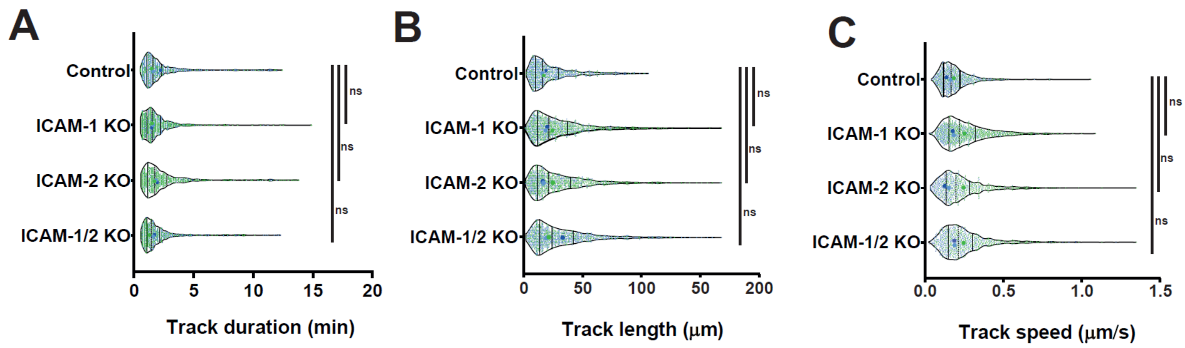

Appendix Figure S4. *ICAM-1/2 KO does not influence neutrophil crawling dynamics.* **(A,B,C)** Violin plots displaying neutrophil crawling duration, length and speed across control (834 neutrophils), ICAM-1 KO (1096 neutrophils), ICAM-2 KO (1036 neutrophils) and ICAM-1/2 KO (793 neutrophils) BOECs. Colours represent data from three independent experiments. Medians of three individual experiments are shown in bigger dots. Medians and quartiles of all data is displayed with vertical lines. One-way Paired ANOVA (n=3) with multiple comparison correction, comparing all conditions with control. **(A)** Control vs ICAM-1: p=0.3906. Control vs ICAM-2: p=0.9299. Control vs ICAM-1/2 KO: p=0.4066 **(B)** Control vs ICAM-1: p=0.6582. Control vs ICAM-2: p=0.9073. Control vs ICAM-1/2 KO: p=0.2270 **(C)** Control vs ICAM-1: p=0.0779. Control vs ICAM-2: p=0.7565. Control vs ICAM-1/2 KO: p=0.0603.

Appendix Figure S5

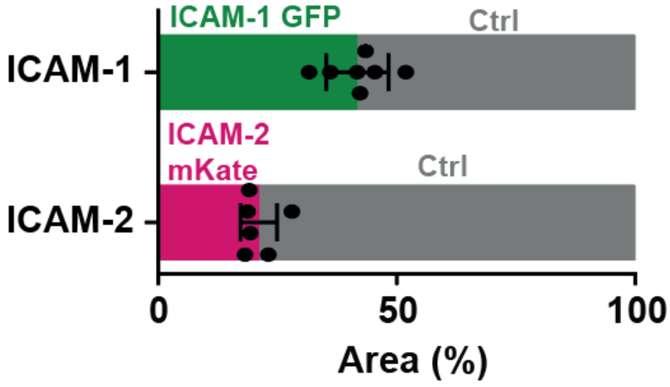

Appendix Figure S5. *Coverage of overexpressed adhesion molecules in the artificial heterogeneity flow assays.* Quantification of area of heterogeneous EC monolayer composing of ICAM-1/ICAM-2 KO ECs either non-expressing (ctrl, grey) or expressing ICAM-1-GFP (upper, green) or ICAM-2-mKate (lower, magenta). Mean with SD is shown.

Appendix Figure S6

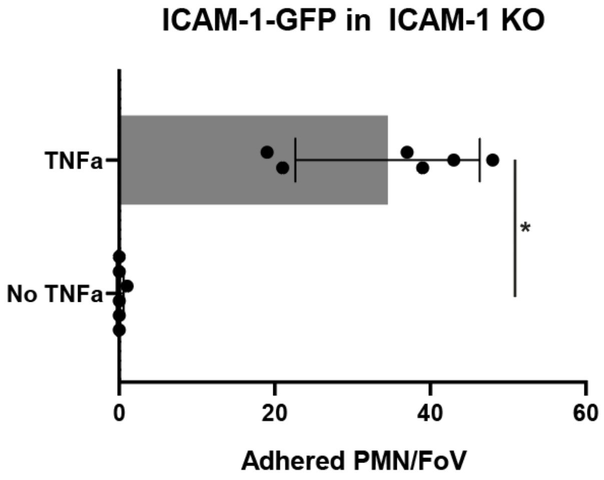

Appendix Figure S6. *ICAM-1 by itself if not enough to allow neutrophil adhesion.* Bar graphs displaying number of adhered neutrophils per Field of View (FoV) in flow experiments performed in ICAM-1 KO BOECs rescued with ICAM-1 GFP, with and without overnight TNFα treatment. Data corresponds to six videos from 3 biological replicates. Mean with SD is shown. Mann Whitney (n=6): p=0.0022.

Appendix Figure S7

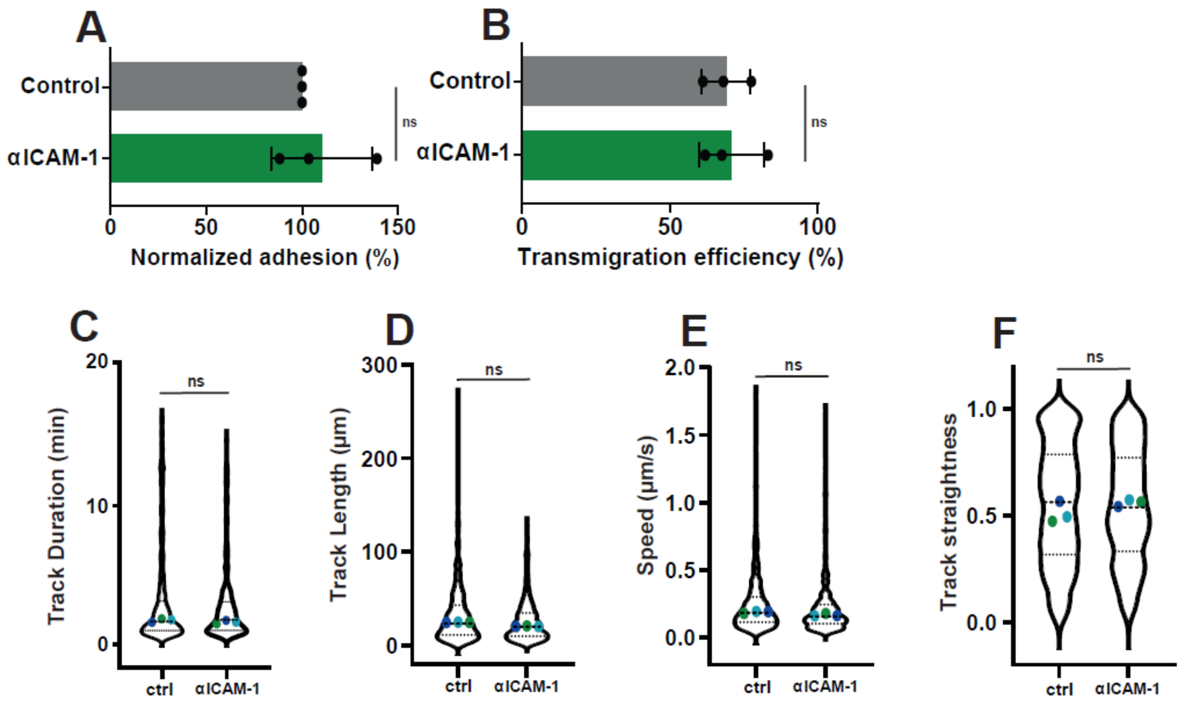

Appendix Figure S7. *The αICAM-1 antibody used for single cell sorting does not influence neutrophil TEM.* **(A)** Quantification of number of adhered neutrophils (PMN) in TEM under flow assay using control HUVECs and HUVECs incubated for 24 hours with an αICAM-1 antibody and treated overnight with TNFα. Data is normalized to control conditions (100%). Data consists of 3 independent experiments, 7038 total neutrophils measured. Bar graph displays mean with SD. Paired t-test (n=3): p=0.5646. **(B)** Quantification of transmigration efficacy of neutrophils (PMN) (total transmigrated/total neutrophils detected *100%) through control HUVECs and HUVECs incubated for 24 hours with an αICAM-1 antibody. Data is normalized to control conditions (100%). Data consists of 3 independent experiments, 7038 total neutrophils measured. Bar graph displays mean with SD. Paired t-test (n=3): p=0.6801. **(C,D,E,F)** Violin plots displaying track duration, length, speed and straightness of neutrophils crawling on control HUVECs (629 neutrophils measured) and HUVECs (486 neutrophils measured) incubated for 24 hours with an αICAM-1 antibody. Medians and quartiles are shown, and three dots are medians of each independent experiment. Paired t-test (n=3) on the medians. **(C)** p=0.4094 **(D)** p=0.2226 **(E)** p=0.2736 **(F)** p=0.9957.

Appendix Figure S8

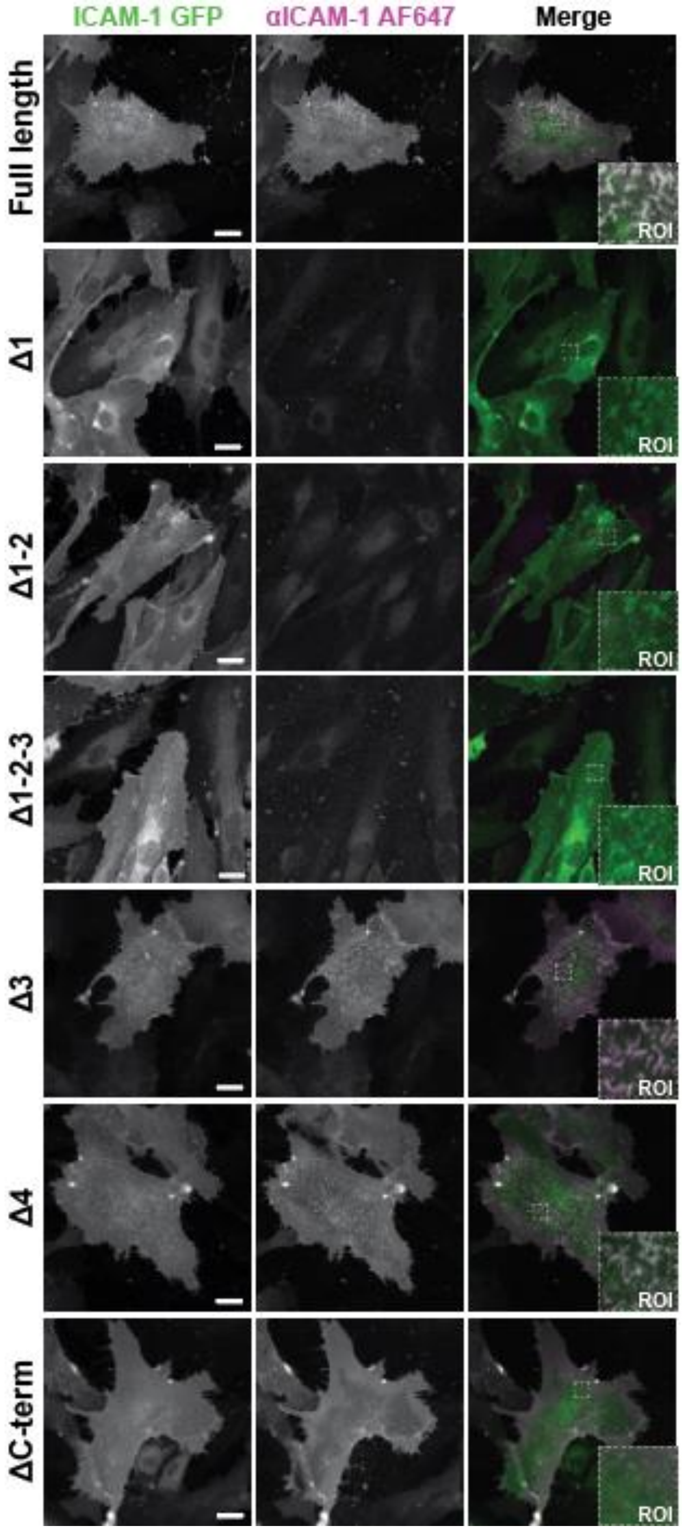

Appendix Figure S8. *ICAM-1 truncations localization in ECs*. Immunofluorescence staining for ICAM-1 Ig-like extracellular domain 1 on all ICAM-1-GFP truncated proteins. Left panel shows ICAM-1-GFP truncated proteins (green), middle pannel shows IF staining for ICAM-1 Ig-like domain 1 (magenta) and right pannel is composite image of both. Scale bar, 20 μm.

Appendix Table S1

Appendix table S1. *All primers used in this study.*

No	Sequence	Use
Gibson cloning		
1	GAACCGTCAGATCCGATGGCTCCCAGCAGCCC	ICAM-1 Δ1
2	TTCTGGAGTCCAGTAGGCATTGCCAGGTCCTGG	
3	TACTGGACTCCAGAACGGG	
4	TCACCATGGTGGCGACCGGTGGATCCAAGGGAG	
1	GAACCGTCAGATCCGATGGCTCCCAGCAGCCC	ICAM-1 Δ1-2
5	AGTCGCTGGCAGGACGGCATTGCCAGGTCCTGG	
6	GTCCTGCCAGCGACTCC	
4	TCACCATGGTGGCGACCGGTGGATCCAAGGGAG	
7	GAACCGTCAGATCCGATGGCTCCCAGCAGCCC	ICAM-1 Δ1-2-3
8	CGCCGGAAAGCTGTAGGCATTGCCAGGTCCTGG	
9	TACAGCTTTCCGGCGCCC	
4	TCACCATGGTGGCGACCGGTGGATCCAAGGGAG	
10	TTAGTGAACCGTCAGATCCGATGGCTCCCAGCAGCCC	ICAM-1 Δ3
11	TTGGGCGCCGGAAAGCTGTAAAAGGTCTGGAGCTGGTAGG	
9	TACAGCTTTCCGGCGCCC	
12	GATCTCACCGGTCCGGGAGGCGTGGCTTGTG	
10	TTAGTGAACCGTCAGATCCGATGGCTCCCAGCAGCCC	ICAM-1 Δ4
13	AGTCGGGGGCCATACAGGACAAAGCTGTAGATGGTCACTG	
14	GTCCTGTATGGCCCCCGA	
15	CTTGCTCACCATGGTGGCGAGGGGAGGCGTGGCTTGTG	
16	GAGATCGCTAGCATGGCTCCCAGCAGCCC	ICAM-1 ΔC
12	GATCTCACCGGTCCGGGAGGCGTGGCTTGTG	
Restriction-based cloning		
17	TACATCTACGTATTAGTCATCGTCA	pLV-mEos4b
18	CCTCTACAAATGTGGTATGGCTGATTATGATC	
Sequencing		
19	TACATCTACGTATTAGTCATCGTCA	pLV-mEos4b
20	GCTGGGCGCACATTCCCTTGATGAA	PCR exon 2 ICAM-1 and sequencing to confirm KO, fw primer.
21	GACCCTACGAGCAAGTGGCAAAGATT	
22	GAGCCAGACTGCCTCAATGGACAG	PCR exon 4 ICAM-2 and sequencing to confirm KO, fw primer.
23	GAGGGGCTCTGTGTGCATTCAGTAG	
