# Supplementary material for: Endothelial transmigration hotspots limit vascular leakage through heterogeneous expression of ICAM‐1
Source: EMBO Rep. 2022 Nov 16;24(1):e55483. doi: 10.15252/embr.202255483 (PMC9827561; doi:10.15252/embr.202255483)
Supplement: Supplementary file 2 — Expanded View Figures PDF [file EMBR-24-e55483-s001.pdf]

Expanded View Figures

Figure EV1. Comparison of different nearest neighbor numbers and example hotspot and non-hotspot tracks.

A Violin plot of average distance to 3 nearest neighbors for actual diapedesis sites and randomly generated spots. Each datapoint corresponds to 1 diapedesis site and 729 datapoints from 21 time lapses are plotted from 3 biological replicates.

B Comparison of analysis methods for nearest neighbor calculations. 1, 3, 5, and 9 nearest neighbor(s) for each TEM spot were calculated and compared with randomly generated spots. Data is from 21 videos from 3 biological replicates. Paired *t*-test ($n = 21$) for every comparison: $*P < 0.0001$ for all.

C Stills from a DIC timelapse TEM assay, showing neutrophils and their complete crawling tracks at hotspots, indicated with blue tracks, and non-hotspots, indicated with a red track. The direction of flow is from top to bottom, time is indicated in min:sec at the top right. Scale bar, 50 μm.

© 2022 The Authors
22 of 21 *EMBO reports* 24: e55483 | 2023 © 2022 The Authors

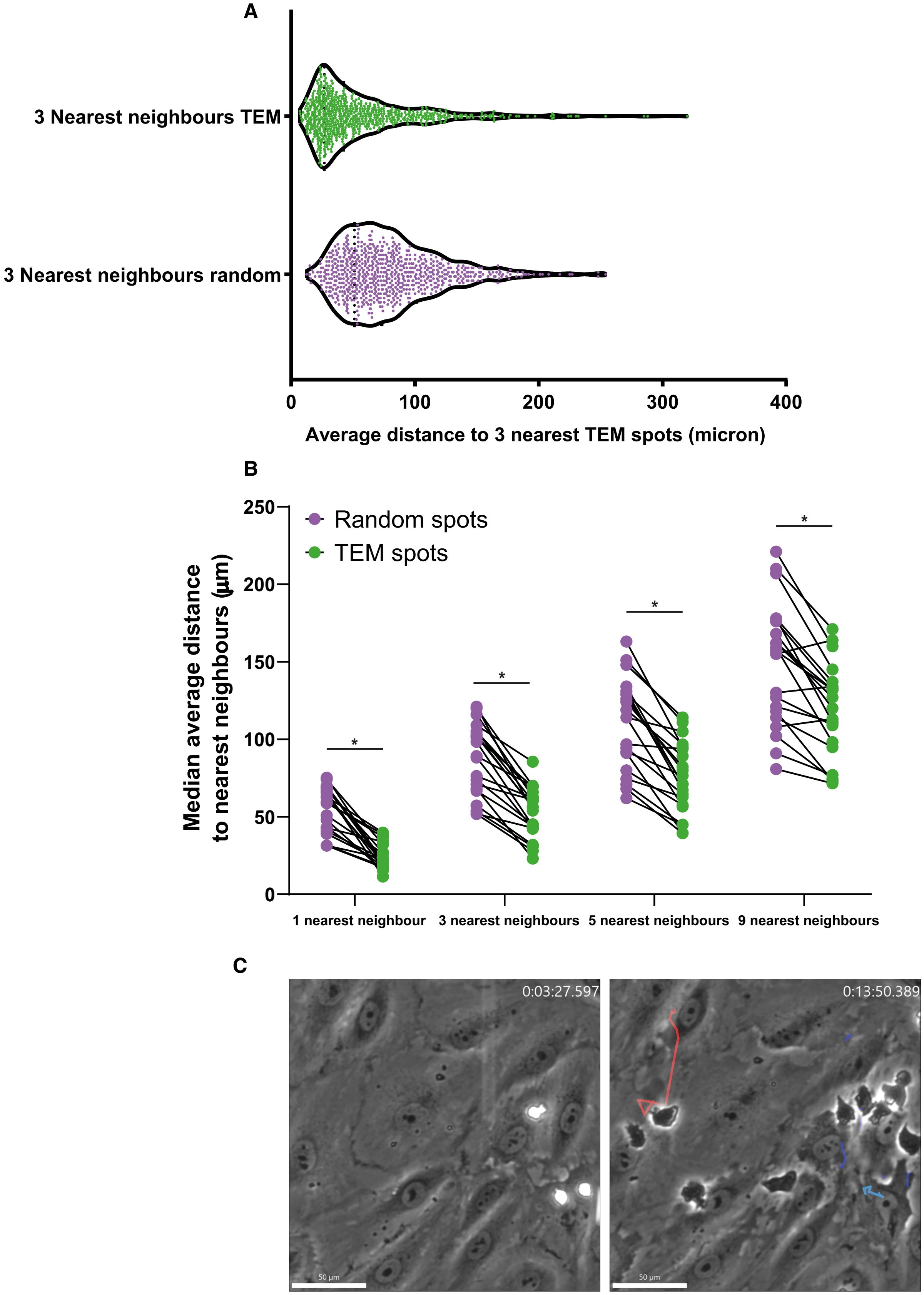
A
3 Nearest neighbours TEM
3 Nearest neighbours random
0
100
200
300
400
Average distance to 3 nearest TEM spots (micron)
B
Random spots
TEM spots
Median average distance to nearest neighbours (µm)
250
200
150
100
50
0
*
1 nearest neighbour
3 nearest neighbours
5 nearest neighbours
9 nearest neighbours
C
0:03:27.597
0:13:50.389
50 µm

Figure EV1.

Figure EV2. ICAM-1 heterogeneity persists across several variables.

A, B Bar graphs displaying coefficient of variations (CoV) of ICAM-1, ICAM-2, and VCAM-1 for each field of view measured. Data are shown from 3 biological replicates. Bars show mean with SD. (A) Different inflammatory stimulants, all overnight incubated. One-way ANOVA on means with multiple comparison correction against TNFα data, separate test for each protein ($n = 9$ images per condition). ICAM-1 (TNFα vs IFN-γ: $*P = 0.0102$. TNFα vs IL-1β: $P = 0.9171$. TNFα vs LPS: $*P < 0.0001$). ICAM-2 (TNFα vs IFN-γ: $P = 0.2900$. TNFα vs IL-1β: $*P = 0.0107$. TNFα vs LPS: $*P = 0.0224$). VCAM-1 (TNFα vs IFN-γ: $*P = 0.0002$. TNFα vs IL-1β: $P = 0.1192$. TNFα vs LPS: $*P = 0.0499$). (B) Different maturation states of the endothelial monolayer were treated overnight with TNFα. One-way ANOVA on means with multiple comparison correction against day 2 data, separate test for each protein ($n = 5$). ICAM-1 (day 2 vs day 3: $P = 0.8627$. day 2 vs day 4: $P = 0.6332$.) ICAM-2 (day 2 vs day 3: $P = 0.1338$. day 2 vs day 4: $*P = 0.0262$.) VCAM-1 (day 2 vs day 3: $P = 0.7364$. day 2 vs day 4: $P = 0.4043$).

C Bar graphs displaying coefficient of variation (CoV) of ICAM-1 expression in confluent HUVECs (6 images) versus subconfluent HUVECs (3 5 × 5 tile scans), treated overnight with TNFα, for each field of view measured. Confluent monolayer Mann–Whitney test ($n = 6$ for confluent and $n = 3$ for subconfluent): $P = 0.5476$. Data are shown from 3 biological replicates. Bars show mean with SD.

D Example of a 5 × 5 tile scan with subconfluent HUVECs, treated overnight with TNFα. ICAM-1 is shown in magenta and nuclei are shown in blue. Scale bar, 300 μm.

E Inverted greyscale LUT of IF stain for ICAM-1, VE-cadherin, and nuclei of a TNFα-treated vessel-on-a-chip composing of HUVECs. Scale bar, 80 μm. Only the bottom half of the Z-stack is shown.

F Side view of whole vessel-on-a-chip, shown in Fig EV2D. Stained for ICAM-1 (green), VE-cadherin (magenta), and nuclei (blue). Scale bar, 20 μm.

G *Ex vivo* whole-mount stains of healthy mesenterial adipose tissue of a carcinoma patient, incubated without or with TNFα for 4 h. ICAM-1 low (green arrow) and ICAM-1 high (blue) cells indicated. ICAM-1 is shown in red, PECAM-1 in magenta, and F-actin in green. Scale bar, 20 μm.

H Violin plot showing the fluorescent intensity of ICAM-1 in *ex vivo* whole-mount stains of healthy mesenterial adipose tissue of a carcinoma patient, incubated without or with TNFα for 4 h. Each datapoint corresponds to 1 EC and 32 (no TNFα) and 123 (with TNFα) datapoints are plotted from 3 biological replicates. Mann–Whitney test ($n = 32$ for no TNFα and $n = 123$ for with TNFα): $*P < 0.0001$.

I Bar graph of the calculated coefficient of variation (CoV; standard deviation/mean) for each field of view image in Fig EV2H. Data are shown from 3 biological replicates. Bar graph shows mean with SD.

 © 2022 The Authors

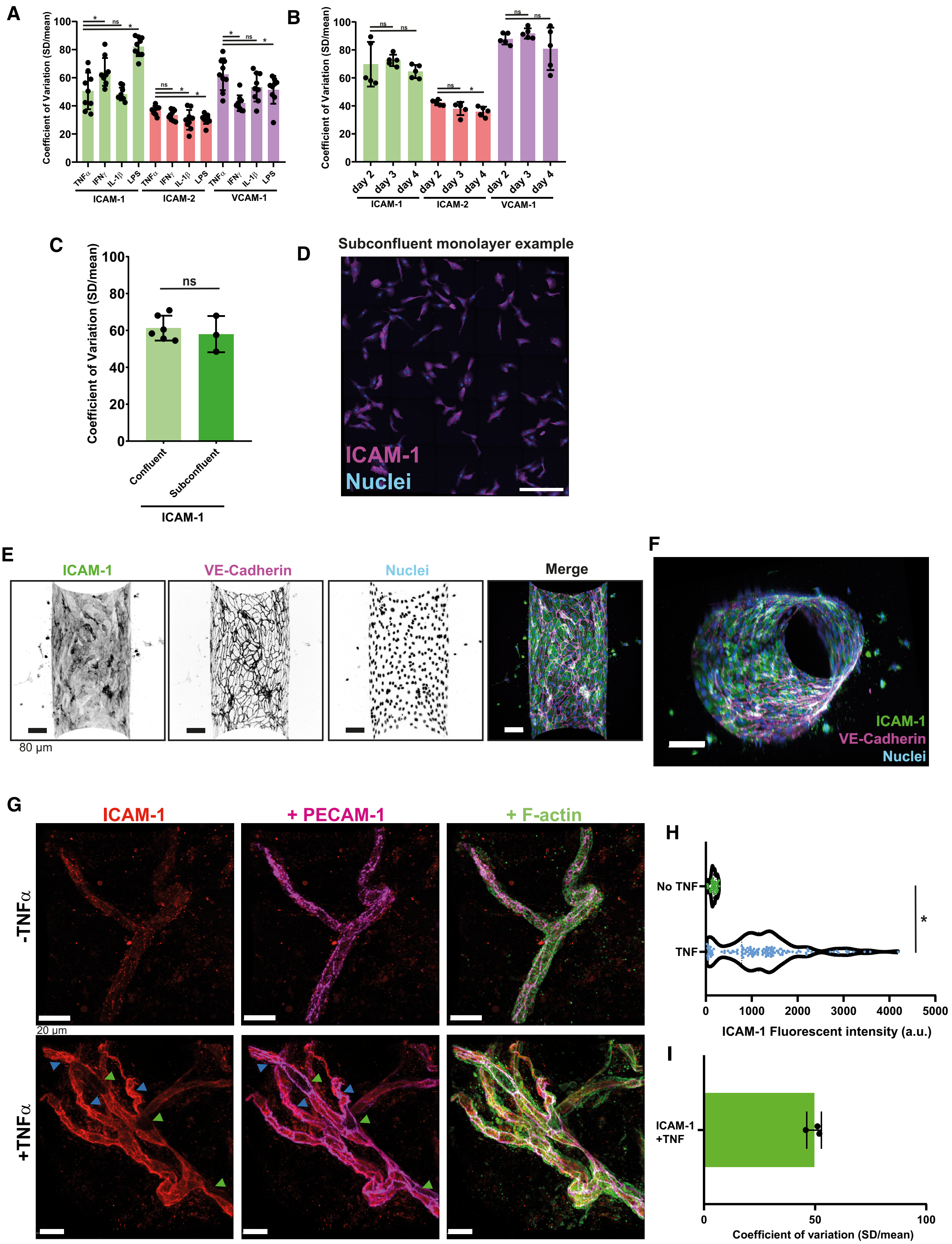
A
B
C
D
E
F
G
H
I
Coefficient of Variation (SD/mean)
TNFα
IFNγ
IL-1β
LPS
ICAM-1
ICAM-2
VCAM-1
day 2
day 3
day 4
ns
Confluent
Subconfluent
Subconfluent monolayer example
ICAM-1
Nuclei
VE-Cadherin
Merge
80 μm
ICAM-1
+ PECAM-1
+ F-actin
-TNFα
+TNFα
20 μm
No TNF
TNF
ICAM-1 Fluorescent intensity (a.u.)
ICAM-1 +TNF
Coefficient of variation (SD/mean)

Figure EV2.

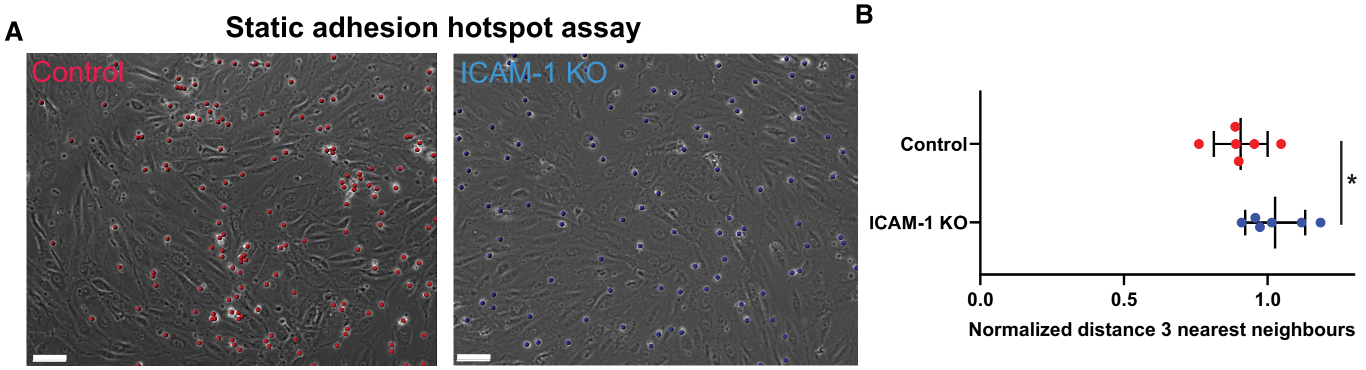

Figure EV3. Neutrophil adhesion hotspots exist in nonflow conditions.

A DIC images of Control and ICAM-1 KO BOECs, treated overnight with TNFα and treated 5 min with neutrophils to allow firm adhesion. Red dots show location of adhered neutrophils on control BOECs, and blue dots show location of adhered neutrophils on ICAM-1 KO BOECs. Scale bar, 70 μm.

B Medians of average distance of adhesion sites or TEM sites to 3 nearest neighbors, normalized against medians of the average distance to three nearest neighbors of the corresponding randomly generated spots. Data from 6 biological replicates are shown. Mann–Whitney: $*P = 0.0411$.

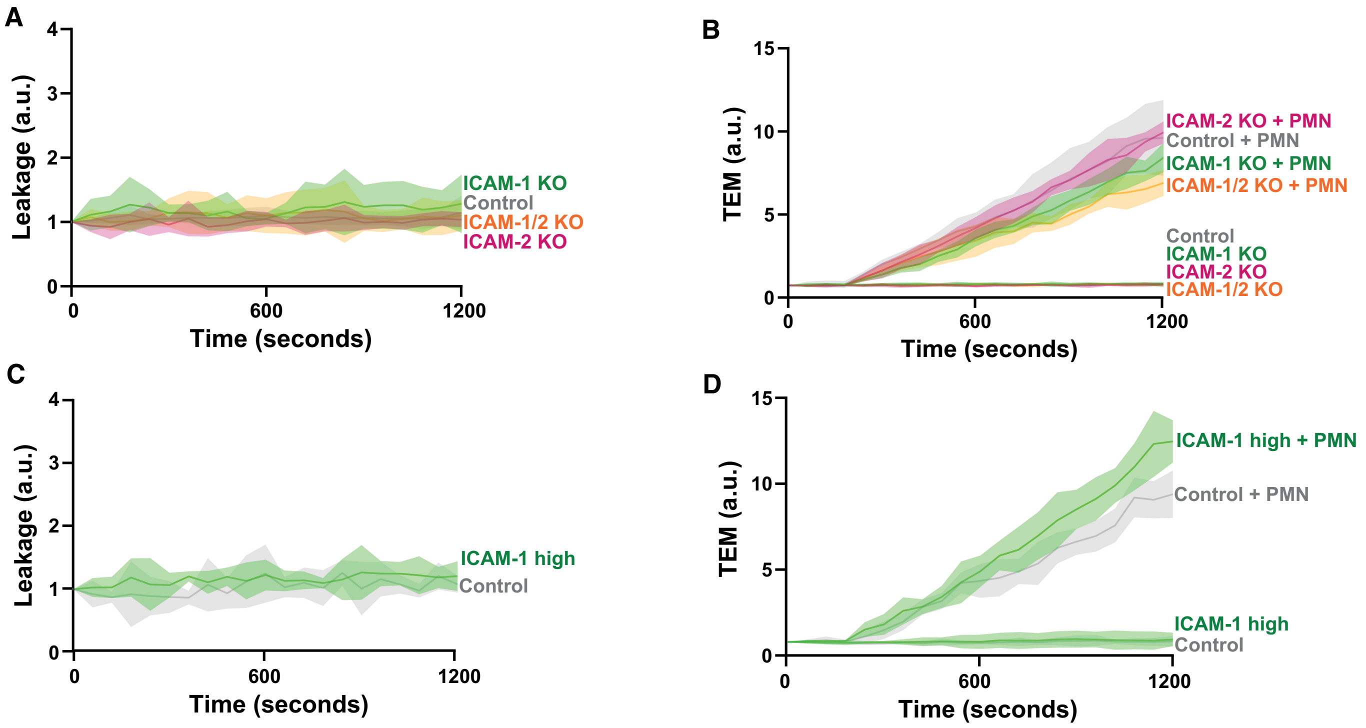

Figure EV4. ICAM-1/2 KO and homogenously sorted ICAM-1 do not show basal leakage.

A Basal leakage measured with Texas-Red-dextran extravasation kinetics through control (gray), ICAM-1 KO (green), ICAM-2 KO (magenta), and ICAM-1/2 KO (orange) BOECs, treated overnight with TNFα. Lines show means with 95% CIs of a total of 6 to 8 wells from 3 biological replicates.

B Neutrophil extravasation kinetics through control (gray), ICAM-1 KO (green), ICAM-2 KO (magenta), and ICAM-1/2 KO (orange) BOECs cultured on 3-μm pore permeable filters. DiO-stained neutrophils transmigrated towards C5a located in the lower compartment. Lines show means with 95% Cis of a total of 6 to 8 wells from 3 biological replicates.

C Basal leakage measured with Texas-Red-dextran extravasation kinetics through control (gray) and ICAM-1 high sorted (green) HUVECs BOECs. Lines show means with 95% Cis of 3 wells from 3 biological replicates.

D Neutrophil extravasation kinetics through control (gray) and ICAM-1 high sorted (green) HUVECs cultured on 3-μm pore permeable filters. DiO-stained neutrophils transmigrated towards C5a located in the lower compartment. Lines show means with 95% CIs of 3 wells from 3 biological replicates.

 © 2022 The Authors

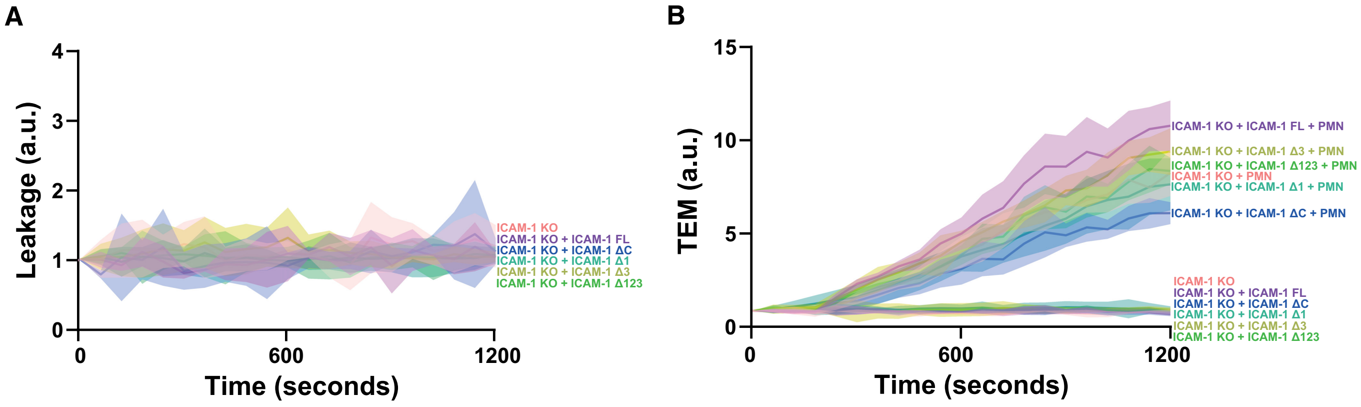

Figure EV5. ICAM-1 truncated construct overexpression in ICAM-1 KO BOECs does not show basal leakage.

A Basal leakage measured with Texas-Red-dextran extravasation kinetics through ICAM-1 KO (pink) BOECs, treated overnight with TNFα, with mosaicly expressed ICAM-1-GFP (purple), ICAM-1-GFP Δ123 (green), ICAM-1-GFP Δ1 (cyan), ICAM-1-GFP Δ3 (yellow) and ICAM-1-GFP ΔC (blue). Lines show means with 95% CIs of 4 wells from 3 biological replicates.

B Neutrophil extravasation kinetics through ICAM-1 KO (pink) BOECs with mosaicly expressed ICAM-1-GFP (purple), ICAM-1-GFP Δ123 (green), ICAM-1-GFP Δ1 (cyan), ICAM-1-GFP Δ3 (yellow) and ICAM-1-GFP ΔC (blue). cultured on 3-μm pore permeable filters. DiO-stained neutrophils transmigrated towards C5a located in the lower compartment. Lines show means with 95% CIs of a total of 4 (without neutrophils) or 8 (with neutrophils) wells from 3 biological replicates.

© 2022 The Authors
