# Supplementary material for: Endothelial transmigration hotspots limit vascular leakage through heterogeneous expression of ICAM‐1
Source: EMBO Rep. 2022 Nov 16;24(1):e55483. doi: 10.15252/embr.202255483 (PMC9827561; doi:10.15252/embr.202255483)
Supplement: Supplementary file 4 [file EMBR-24-e55483-s004.pdf]

EMBO reports

Endothelial transmigration hotspots limit vascular leakage through heterogeneous expression of ICAM1

Max Gronloh, Janine Arts, Sebastián Palacios Martínez, Amerens van der Veen, Lanette Kempers, Abraham van Steen, Joris Roelofs, Martijn Nolte, Joachim Goedhart, and Jaap van Buul
DOI: 10.15252/embr.202255483

Corresponding author: Jaap van Buul (j.vanbuul@sanquin.nl)

Review Timeline:

Submission Date:	25th May 22
Editorial Decision:	27th Jun 22
Revision Received:	14th Sep 22
Editorial Decision:	11th Oct 22
Revision Received:	25th Oct 22
Accepted:	28th Oct 22

Editor: Achim Breiling

Transaction Report:

(Note: With the exception of the correction of typographical or spelling errors that could be a source of ambiguity, letters and reports are not edited. Depending on transfer agreements, referee reports obtained elsewhere may or may not be included in this compilation. Referee reports are anonymous unless the Referee chooses to sign their reports.)

1st Editorial Decision 27th Jun 2022

Dear Prof. van Buul,

Thank you for the submission of your research manuscript to EMBO reports. I have now received the reports from the three referees that were asked to evaluate your study, which can be found at the end of this email.

As you will see, the referees think that these findings are of interest. However, all referees have several comments, concerns, and suggestions, indicating that a major revision of the manuscript is necessary to allow publication of the study in EMBO reports. As the reports are below, and all their points need to be addressed, I will not detail them here.

Given the constructive referee comments, we would like to invite you to revise your manuscript with the understanding that all referee concerns must be addressed in the revised manuscript and in a detailed point-by-point response. Acceptance of your manuscript will depend on a positive outcome of a second round of review. It is EMBO reports policy to allow a single round of revision only and acceptance of the manuscript will therefore depend on the completeness of your responses included in the next, final version of the manuscript.

Revised manuscripts should be submitted within three months of a request for revision. Please contact me to discuss the revision (also by video chat) if you have questions or comments regarding the revision, or should you need additional time.

When submitting your revised manuscript, please also carefully review the instructions that follow below.

PLEASE NOTE THAT upon resubmission revised manuscripts are subjected to an initial quality control prior to exposition to re-review. Upon failure in the initial quality control, the manuscripts are sent back to the authors, which may lead to delays. Frequent reasons for such a failure are the lack of the data availability section (please see below) and the presence of statistics based on n=2 (the authors are then asked to present scatter plots or provide more data points).

When submitting your revised manuscript, we will require:

1) a .docx formatted version of the final manuscript text (including legends for main figures, EV figures and tables), but without the figures included. Figure legends should be compiled at the end of the manuscript text.

2) individual production quality figure files as .eps, .tif, .jpg (one file per figure), of main figures and EV figures. Please upload these as separate, individual files upon re-submission.

The Expanded View format, which will be displayed in the main HTML of the paper in a collapsible format, has replaced the Supplementary information. You can submit up to 5 images as Expanded View. Please follow the nomenclature Figure EV1, Figure EV2 etc. The figure legend for these should be included in the main manuscript document file in a section called Expanded View Figure Legends after the main Figure Legends section. Additional Supplementary material should be supplied as a single pdf file labeled Appendix. The Appendix should have page numbers and needs to include a table of content on the first page (with page numbers) and legends for all content. Please follow the nomenclature Appendix Figure Sx, Appendix Table Sx etc. throughout the text, and also label the figures and tables according to this nomenclature.

For more details, please refer to our guide to authors:
http://www.embopress.org/page/journal/14693178/authorguide#manuscriptpreparation

Please consult our guide for figure preparation:
http://wol-prod-cdn.literatumonline.com/pb-assets/embo-site/EMBOPress_Figure_Guidelines_061115-1561436025777.pdf

See also the guidelines for figure legend preparation:
https://www.embopress.org/page/journal/14693178/authorguide#figureformat

3) a complete author checklist, which you can download from our author guidelines (https://www.embopress.org/page/journal/14693178/authorguide). Please insert page numbers in the checklist to indicate where the requested information can be found in the manuscript. The completed author checklist will also be part of the RPF.

Please also follow our guidelines for the use of living organisms, and the respective reporting guidelines:
http://www.embopress.org/page/journal/14693178/authorguide#livingorganisms

4) that primary datasets produced in this study (e.g. RNA-seq, ChIP-seq, structural and array data) are deposited in an appropriate public database. If no primary datasets have been deposited, please also state this in a dedicated section (e.g. 'No primary datasets have been generated and deposited'), see below.

See also: http://embor.embopress.org/authorguide#datadeposition

Please remember to provide a reviewer password if the datasets are not yet public.

The accession numbers and database should be listed in a formal "Data Availability" section (placed after Materials & Methods) that follows the model below. This is now mandatory (like the COI statement). Please note that the Data Availability Section is restricted to new primary data that are part of this study. This section is mandatory. As indicated above, if no primary datasets have been deposited, please state this in this section

Data availability

The datasets produced in this study are available in the following databases:

- RNA-Seq data: Gene Expression Omnibus GSE46843 (https://www.ncbi.nlm.nih.gov/geo/query/acc.cgi?acc=GSE46843)
- [data type]: [name of the resource] [accession number/identifier/doi] ([URL or identifiers.org/DATABASE:ACCESSION])

*** Note - All links should resolve to a page where the data can be accessed. ***

Moreover, I have these editorial requests:

6) We strongly encourage the publication of original source data with the aim of making primary data more accessible and transparent to the reader. The source data will be published in a separate source data file online along with the accepted manuscript and will be linked to the relevant figure. If you would like to use this opportunity, please submit the source data (for example scans of entire gels or blots, data points of graphs in an excel sheet, additional images, etc.) of your key experiments together with the revised manuscript. If you want to provide source data, please include size markers for scans of entire gels, label the scans with figure and panel number, and send one PDF file per figure.

7) Our journal encourages inclusion of *data citations in the reference list* to directly cite datasets that were re-used and obtained from public databases. Data citations in the article text are distinct from normal bibliographical citations and should directly link to the database records from which the data can be accessed. In the main text, data citations are formatted as follows: "Data ref: Smith et al, 2001" or "Data ref: NCBI Sequence Read Archive PRJNA342805, 2017". In the Reference list, data citations must be labeled with "[DATASET]". A data reference must provide the database name, accession number/identifiers and a resolvable link to the landing page from which the data can be accessed at the end of the reference. Further instructions are available at: http://www.embopress.org/page/journal/14693178/authorguide#referencesformat

8) Regarding data quantification and statistics, please make sure that the number "n" for how many independent experiments were performed, their nature (biological versus technical replicates), the bars and error bars (e.g. SEM, SD) and the test used to calculate p-values is indicated in the respective figure legends (also for potential EV figures and all those in the final Appendix). Please also check that all the p-values are explained in the legend, and that these fit to those shown in the figure. Please provide statistical testing where applicable. Please avoid the phrase 'independent experiment', but clearly state if these were biological or technical replicates. See also:
http://www.embopress.org/page/journal/14693178/authorguide#statisticalanalysis

9) Please also note our reference format:
http://www.embopress.org/page/journal/14693178/authorguide#referencesformat

10) Please add scale bars of similar style and thickness to all the microscopic images (main and EV figures), using clearly visible black or white bars (depending on the background). Please place these in the lower right corner of the images themselves. Please do not write on or near the bars in the image but define the size in the respective figure legend.

11) We updated our journal's competing interests policy in January 2022 and request authors to consider both actual and perceived competing interests. Please review the policy https://www.embopress.org/competing-interests and update your competing interests if necessary. Please name this section 'Disclosure and Competing Interests Statement' and put it after the Acknowledgements section.

12: Please remove the list of abbreviations from the manuscript. Please make sure abbreviations are defined the first time they are used in the manuscript.

I look forward to seeing a revised version of your manuscript when it is ready. Please let me know if you have questions or comments regarding the revision.

Yours sincerely,

Achim Breiling
Senior Editor
EMBO Reports

Referee #1:

The manuscript by Dr. Grönloh and colleagues explored the hypothesis that heterogeneous surface expression of ICAM-1 in endothelium plays an important role in promoting hotspots of neutrophil adhesion and modulating monolayer permeability (loss of barrier) during neutrophil diapedesis.

The major observations were: 1) Authors confirmed prior reports that neutrophil TEM hotspots occur in vitro in TNF, LPS, IFN-gamma or IL-1-stimulated HUVEC (new here is with BO ECs) using a new analytic/quantitative method;
2) Confirmed prior reports that ICAM-1 surface expression is heterogeneous in cytokine stimulated HUVEC monolayers; new here was same finding in Authors' 3D vessel-on-a chip model;
3) In contrast to ICAM-1 expression patterns, ICAM-2 expression is not as heterogeneously expressed, included images of discarded human surgical tissue stained for ICAM-1/PECAM-1 (but lacked quantification);
4) TEM hotspots correlate with high surface expressed ICAM-1 in ECs but less so with ICAM-2;
5) Authors propose (Fig 4) that presence of ICAM-1 in EC prevents loss of barrier neutrophil in vitro using a transwell and no-flow (static) permeability assay. These data are not convincing as not clear that neutrophils form TEM hotspots under static conditions.
6) ICAM-1 mutants demonstrate that Ig loops 1-3 were involved in formation of TEM hotspots.

Suggestions/comments.
1. Major Comment and Discussion lines 376-85: The Authors' premise is ICAM-1 is crucial for TEM hotspots. Yet Authors' data do not support a role for ICAM-1 in neutrophil adhesion! Given these data, one has to question the validity of the assay used here. There is strong evidence in literature that ICAM-1 and ICAM-2 function in neutrophil adhesion to cytokine or LPS activated EC in vitro or in vivo. Authors did cite three refs. for this, but ignore the more accepted references that supporting ICAM-1 functions in neutrophil adhesion and arrest on EC via binding LFA-1Mac-1 integrins on neutrophils. See these refs and refs within these publications (Smith CW. J Clin. Invest. 1989, 83:2008-2017; J. Immunol. 1999, 163:5029-5038; Blood 2006, 107:4721; Blood 2009, 113:6246; Blood 2005, 106:584;). This lessens my enthusiasm for this report.

2. Authors propose (Fig 4) that presence of ICAM-1 in EC prevents loss of barrier neutrophil in vitro using a transwell and no-flow (static) permeability assay. These data are not convincing for multiple reasons. A missing data set is demonstration that neutrophils form aggregates and TEM hotspots under static conditions in such assays. Specifically, the methods section (page 21) indicates a chemoattractant C5a and 24 hr TNF-alpha treated endothelium, which is distinctly different from flow study methods using throughout report. See comment 6 below. Finally, TNF-alpha treatment of EC in vitro for 24 hr is known to induce significant leakage in HUVEC yet Authors do not observed any leakage in Fig 5 (Ref for TNF induced perm change by ECIS using BAEC but similar findings in HUVEC Am J Physiol Lung Cell Mol Physiol. 2001 Jun;280(6):L1168-78).

Other Comments:
3. Sites of TEM could differentially impact permeability. Do the neutrophils use the paracellular (junctional) or non-junctional routes of TEM in these assays? This is physiologically relevant because different routes of TEM (junction vs non-junctional) may differentially impact barrier function. Older literature found that longer times of TNF treatment led to greater amounts of ICAM-1 and a significant increase in non-junctional TEM. Did Authors observe non-junctional TEM at hot spots?

4. Authors ignore other possible contributors to neutrophil TEM hotspots: E-selectin is inducible in parallel with ICAM-1, and E-selectin assists in activating neutrophil LFA-1 integrins to bind ICAM-1 and mediate arrest and adhesion/migration to TEM sites. Authors focus on ICAM-1 but clearly other molecules play important roles. Including the surface expression/distribution of E-selectin in these TEM assay models would be important. Likewise prior reports found that intracellular pools/locations of chemokines play crucial roles in driving leukocyte TEM in vivo and in vitro (doi: 10.1016/j.immuni.2018.09.018; doi: 10.1038/ni.2173).

5. Figure 2 and Sfig 2. Please address the following comment in Discussion: One possible explanation for heterogeneity of ICAM-1 or VCAM-1expression is that your HUVEC cultures were prepared from multiple umbilical cords, often up to 20+ or more at commercial suppliers of HUVEC. Do you see the same heterogeneity in a culture from a single cord donor?

6. Figure legend 1 and page 6 Results. Include length of time that EC were treated with TNF-alpha. Longer times of treatment (> 24h) vs shorter treatment times (4 - 6 h) leads to greater amounts of surface ICAM-1. It is more physiological that using transfection and CRISPR editing

7. Fig S2 Images of discarded human surgical tissue stained for ICAM-1/PECAM-1 should include quantification.

Minor.
Ref 8 is incomplete?
Line 273, mislabeled (Fig 7A-B)?
Line 326, misspelled recognition.

Referee #2:

The manuscript by Grönloh et al. analyzes the role of ICAM-1 in recognizing TEM hotspots for migrating neutrophils under inflammatory conditions and assess the significance of these hotspots in limiting endothelial permeability during neutrophil extravasation. Using a combination of approaches, including Crispr/Cas9, ICAM-1 mutants, photo-convertible probe 36 mEos4b, and confocal and time lapse microscopy, neutrophil transmigration and endothelial permeability assays, data are presented to reveal the existence of neutrophil TEM hotspots in the endothelial monolayer that are regulated by ICAM-1 heterogeneity to limit vascular leakage during neutrophil extravasation. Evidence is also presented to show that the first 3 extracellular Ig-like domains of ICAM-1 are required whereas the intracellular tail of ICAM-1 is dispensable for hotspot recognition.

Although these findings are new and potentially interesting, the consideration of following points would further enhance the manuscript:

1. Increased ICAM-1 expression either by treatment with TNF or transfection of ICAM-1 cDNA each induces TEM hotspots. However, these experiments don't distinguish the additional effects of TNF (in addition to increasing the levels of ICAM-1) on ICAM-1 (confirmational changes or post-translational modifications) that may also contribute to ICAM-1-dependent TEM hotspots. It would be interesting to re-express ICAM-1 in ICAM-1 KO endothelial cells and treat them with or without TNF and monitor ICAM-1 heterogeneity and TEM hotspots to see if these effects are more pronounced in TNF-treated cells.

2. Inclusion of an experiment in which extracellular domain of ICAM-2 or VCAM-1 is replaced with that of ICAM-1 (containing all five Ig-like domains or just first 3 domains) would not only strengthen the claim that ICAM-1 extracellular domain is not only sufficient but can render VCAM-1 or ICAM-2 capable of regulating TEM hotspots and limiting vascular permeability during neutrophil TEM.

3. It would be interesting to determine how endothelial permeability during TEM is affected when neutrophils treated with blocking anti-LFA-1 or anti-Mac-1 antibody alone and in combination are used.

4. Experiments in Fig. 6C lacks data with ICAM-1 mutant lacking Ig-like domain 1 or 3 as controls.

5. The labels in Fig. 2A are barely legible.

Referee #3:

The manuscript by Grönloh and colleagues makes the argument that heterogeneous ICAM-1 expression on HUVEC or blood outgrowth ECs creates hotspots for PMN transmigration, and that in this configuration, TEM of neutrophils is not associated with an increase in dextran permeability, either during or after TEM. This study nicely quantifies many aspects of ICAM-1 and -2 dependent binding, crawling, and subsequent TEM of purified human neutrophils on human EC monolayers in vitro. The imaging methods and quantitative algorithms used have provided very interesting and novel data. There are a few minor details that need clarification. From my perspective, putting this data into the ICAM-1 and PECAM-1 dependent TEM literature, which generally has focused on signaling of TEM, without assessing permeability, requires a bit more integration and discussion.

Specific Comments:
1. The study makes the interesting observation that ICAM-1/2 hotspots promote TEM which somehow avoids a simultaneous increase in permeability to dextran (ie., paracellular permeability). So where are these hotspots in the monolayer? Are they near "tricellular junctions", for example? These have been referred to as the preferred sites of TEM, but with little documentation as to why, and thus addressing this old concept with new data seems relevant here. It would be interesting if results of the current study could clarify the location of hotspots relative to cell-cell junctions, especially PECAM staining, which I presume is required for TEM in the studies presented.

2. Does EC ICAM-1 staining on individual cells change when the monolayer becomes confluent? Ie., Is formation of the "hotspots" driven by the patterning of the monolayer (e.g., associated with tricellular or other configuration?), or is it in inherent in the individual EC?

3. Alternatively, or perhaps in addition, are the hotspots concentrated on individual cells such that "activation" of ICAM-1 can then drive mobilization of caveolae clusters to facilitate transmigration via a transcellular route or promote PECAM-1 membrane

localization from the Lateral Border Recycling Compartment for binding and transmigration of neutrophils via the paracellular route. It was shown previously that ICAM-1 clustering induces NO and Src signaling that lead to ICAM-1, PECAM-1 and Cav1 phosphorylation. Some discussion on whether hotspots represent or give rise to clustered ICAM-1 that upon binding to PMN will promote their TEM via signaling that controls the transcellular or paracellular route. It was unexpected to this reviewer that the C-terminus of ICAM-1 would not be required for PMN TEM. Is this controversial ? Seems some discussion related to the role of the C-term ITAM motif on both ICAM-1 and 2 is warranted as this relates to the ability of TNF to not only increase ICAM-1 expression, but also the increase in phosphorylation-dependent binding avidity to enhance PMN binding and TEM.

4. As there was no effect of ICAM-1 KO on PMN TEM, is it your conclusion that TEM is dependent on PECAM-1 at cell-cell junctions? If so, what are CAMs are these PMN initially adhering to on the surface? ICAM-2 for sure. DKO of ICAM-1/2 did reduce TEM by 50%, but what other CAMs are participating in firm adhesion of PMN?

5. Images of ICAM-1 and PECAM-1 in human microvessels do not seem to add anything or make a strong case in support of the in vitro studies. Also, what is the purpose of showing the vessel on a chip staining? This also does not seem to add information.

6. The following sentence on pg 17 related to the method of neutrophil counting is confusing:
"Only spots with a quality higher than 80 were filtered to ensure only neutrophils were counted."

Where does this quality number come from?

7. Monolayer permeability assessed by accumulation of 70kDa TR-dextran below the filter insert will reflect changes in junctional integrity based on molecule size and disruption of adherens or tight junctions (paracellular pathway). Considering ICAM-1 activation via PMNs or antibody crosslinking can stimulate Src and trafficking of caveolae by promoting Cav1 phosphorylation, it would perhaps be more interesting and for sure more physiological to assess Au-albumin permeability by transmission EM as a function of PMN TEM, and to relate this to paracellular vs transcellular transport of albumin.

8. A conclusion of this study could be that heterogeneous ICAM-1 expression prevents activated PMN from increasing monolayer permeability to macromolecules. Is there evidence for this in the ICAM-1-/- mouse? i.e., Do activated PMN increase endothelial permeability to a greater extent in absence of ICAM-1 expression?

We thank reviewer 1 for his/her thoughtful comments. We have addressed all the comments to the best of our ability in a point-by-point reply, listed below.

Referee #1:
The manuscript by Dr. Grönloh and colleagues explored the hypothesis that heterogeneous surface expression of ICAM-1 in endothelium plays an important role in promoting hotspots of neutrophil adhesion and modulating monolayer permeability (loss of barrier) during neutrophil diapedesis.

The major observations were:

1) Authors confirmed prior reports that neutrophil TEM hotspots occur in vitro in TNF, LPS, IFN-gamma or IL-1-stimulated HUVEC (new here is with BO ECs) using a new analytic/quantitative method;

2) Confirmed prior reports that ICAM-1 surface expression is heterogeneous in cytokine stimulated HUVEC monolayers; new here was same finding in Authors' 3D vessel-on-a chip model;

3) In contrast to ICAM-1 expression patterns, ICAM-2 expression is not as heterogeneously expressed, included images of discarded human surgical tissue stained for ICAM-1/PECAM-1 (but lacked quantification);

4) TEM hotspots correlate with high surface expressed ICAM-1 in ECs but less so with ICAM-2;

5) Authors propose (Fig 4) that presence of ICAM-1 in EC prevents loss of barrier neutrophil in vitro using a transwell and no-flow (static) permeability assay. These data are not convincing as not clear that neutrophils form TEM hotspots under static conditions.

6) ICAM-1 mutants demonstrate that Ig loops 1-3 were involved in formation of TEM hotspots.

Suggestions/comments.
1. Major Comment and Discussion lines 376-85: The Authors' premise is ICAM-1 is crucial for TEM hotspots. Yet Authors' data do not support a role for ICAM-1 in neutrophil adhesion! Given these data, one has to question the validity of the assay used here. There is strong evidence in literature that ICAM-1 and ICAM-2 function in neutrophil adhesion to cytokine or LPS activated EC in vitro or in vivo. Authors did cite three refs. for this, but ignore the more accepted references that supporting ICAM-1 functions in neutrophil adhesion and arrest on EC via binding LFA-1Mac-1 integrins on neutrophils. See these refs and refs within these publications (Smith CW. J Clin. Invest. 1989, 83:2008-2017; J. Immunol. 1999, 163:5029-5038; Blood 2006, 107:4721; Blood 2009, 113:6246; Blood 2005, 106:584;). This lessens my enthusiasm for this report.

We agree with the reviewer that a prominent role for ICAM-1 in leukocyte adhesion and TEM has been widely described. And, to our surprise, when we depleted ICAM-1 from endothelial cells using CRISP9/Cas technology, the inhibitory effects on TEM were limited. However, it was not our intention to state that there is no supportive role for ICAM-1 in TEM. It might very well be that when ICAM-1 is not available, leukocytes and in particular neutrophils (because of their repertoire of integrins LFA-1 and Mac-1) find other ways to firmly adhere to the endothelium. One of those potential backup systems might be ICAM-2. Indeed, when we depleted ICAM-1 and -2, we found a significant reduction in the number of neutrophils that crossed the endothelium.

Interestingly, when we block beta2 integrins (i.e., both LFA-1 and Mac-1) on the neutrophils, we do block adhesion to the endothelium. Also, when using neutrophils from Leukocyte-Adhesion Deficiency (LAD)-patients, (these neutrophils show impaired beta2 integrin function), hardly any neutrophil would adhere to the inflamed endothelium. We have added these data for the reviewer (R1_Figure 1). In addition, it has been reported that LAD-1 deficiencies lead to higher circulating neutrophil numbers compared to ICAM-1 KO ECs (Xu *et al*., 1994, Journal of Exp. Med.) Thus, these experiments and previous studies indicate that the beta2 integrins are more crucial for adhesion than ICAM-1 alone. Nevertheless, ICAM-1 has a strong supportive role in leukocyte adhesion and TEM.

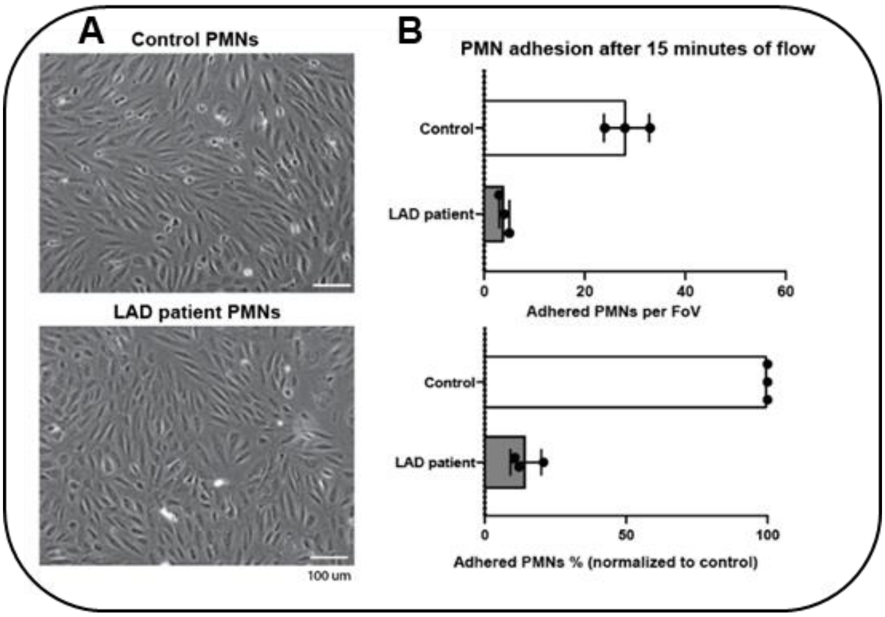

R1_Figure 1. Transmigration assay under flow conditions using wt and LAD1 neutrophils. A. Left panels to show endothelial monolayers after overnight TNFα treatment. Bar, 100 µm. B. Right graphs show quantification of the number of adherent neutrophils per field of view after 15 minutes of flow (upper graph), and a relative number of neutrophils that adhere to an endothelial monolayer in percentage (lower graph). The experiment is done three times independently of each other.

The papers referred to by the reviewer cover in particular the role of the integrins LFA-1 and Mac-1, the receptors for ICAM-1. Data from Smith CW et al., 1989, and the publication in 1999 in J Immunol show the dependence of the adhesion and TEM of leukocytes on LFA-1 and Mac-1 in this respect. Issekutz *et al* (1999, Journal of Leukocyte Biology) demonstrated that blocking LFA-1 and Mac-1 caused significant decreases in neutrophil adhesion, whereas ICAM-1 blocking by itself did not cause a significant decrease. In accordance with our data, they only observed significant adhesion defects when both ICAM-1 and ICAM-2 on the endothelium were blocked simultaneously.

The paper in Blood, 2006, from the Nourshargh lab, shows that ICAM-2 plays an important role in TEM in mouse models. The 2009 paper from the same lab shows that other adhesion molecules such as JAM-A and PECAM-1 play a supporting role, next to ICAM-2. In fact, they demonstrate that analysis of the site of arrest of neutrophils in inflamed tissues from ICAM-2(-/-), JAM-A(-/-), and PECAM-1(-/-) mice indicate that these molecules act in sequence to mediate transmigration.

The 2005 Blood paper from the Luscinskas lab shows that overexpression of ICAM-1 triggers transcellular migration. We have confirmed these data, also for T-cells, and recently published these data (Schoppmeyer et al, Cell Reports, 2022). Interestingly, they show that when ICAM-1 was overexpressed in TNFα-treated endothelium, no additional TEM was detected compared to 4 hours of control TNFα-treated endothelium. Only after 24 hours TNFα treatment, a clear increase in the number of neutrophils that transmigrated was detected. These data show that the role of ICAM-1 as the sole determinant of leukocyte adhesion is limited.

Nevertheless, we do agree with the reviewer that we should highlight these studies in the discussion section.

Regarding T-cells, we have also perfused CD8+ T-cells over ICAM-1 and/or ICAM-2 KO endothelial cells, as it was reported that T-cells do depend on ICAM-1 (Steiner et al., J Immunol 2010; Lyck et al., Blood, 2003). Here we did find a significant decrease in adhesion when knocking out only ICAM-1. We have added these data for the reviewer (R1_Figure 2). For our report, we focussed on neutrophils, but these data could indicate that the role of ICAM-1 in total adhesion numbers might be more prominent for the T-cell population. Interestingly, we also found that the diapedesis efficacy of CD8+ T-cells was reduced in ICAM-1 KO conditions, further supporting the hypothesis of different roles of ICAM-1 for different leukocyte subsets.

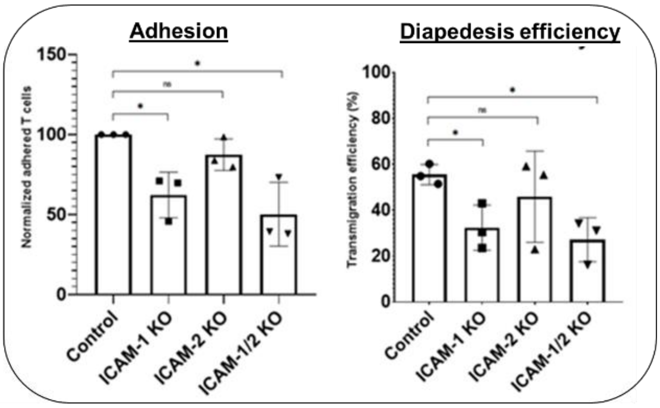

R1_Figure 2. Transmigration of CD8+ T cells across the endothelium. The left graph represents T-cell adhesion to endothelial cells that had an ICAM-1 KO, ICAM-2 KO, double KO or were left untreated (Control). The right graph shows diapedesis efficiency across ECs as described above**.**

2. Authors propose (Fig 4) that presence of ICAM-1 in EC prevents loss of barrier neutrophil in vitro using a transwell and no-flow (static) permeability assay. These data are not convincing for multiple reasons. A missing data set is demonstration that neutrophils form aggregates and TEM hotspots under static conditions in such assays. Specifically, the methods section (page 21) indicates a chemoattractant C5a and 24 hr TNF-alpha treated endothelium, which is distinctly different from flow study methods using throughout report. See comment 6 below. Finally, TNF-alpha treatment of EC in vitro for 24 hr is known to induce significant leakage in HUVEC yet Authors do not observed any leakage in Fig 5 (Ref for TNF induced perm change by ECIS using BAEC but similar findings in HUVEC Am J Physiol Lung Cell Mol Physiol. 2001 Jun;280(6):L1168-78).

The reviewer raises a good point regarding the lack of proof of the presence of hotspots in our static leakage Transwell assay. Unfortunately, it is not possible to perform microscopy experiments using the Transwell plates. To show that hotspots do exist in a Transwell setup and in the absence of flow, we seeded endothelial cells that were deficient for ICAM-1 or wt on a 2D coverslip that had the same diameter as the 24-wells Transwell insert. We then added the same number of neutrophils as we would add to the Transwell assay and took microscopy images after 5 minutes of incubation, enough for adhesion to occur. In static conditions without a chemoattractant below the endothelial monolayer, we were able to demonstrate the formation of neutrophil adhesion hotspots on control BOECs, which was absent on ICAM-1 KO BOECs under static conditions. We concluded that flow is not necessary to induce ICAM-1-mediated hotspots. We have added this new data set to the manuscript as Figure EV3.

We agree with the reviewer that TNFα reduces endothelial resistance in a timely manner. We underscore these findings. The reduction in resistance measured in the paper by Petrache and co-workers (Am J Physiol Lung Cell Mol Physiol, 2001) showed that 10 ng/mL TNFα, which was used in our experiments, leads to a reduction between 10-20% after 16 hours of treatment, after which a new plateau is reached. We wish to stress that we performed our experiments after 20 hours of TNFα treatment, i.e., once a plateau was reached, and any increases in leakage were measured from that time point on. It is important to state this, therefore, we have added this comment to our revised manuscript in the Method section.

Previous data from our lab (Heemskerk et al, Nat Comm, 2014, figure S1C*)*, and other labs (Braun et al., Blood, 2020; Wessel et al., Nat Immunol 2014; Petri et al., Blood, 2011; Zeng et al., Am J Physiol-Heart and Circ Physiol, 2002; Baluk et al., Am J Pathol 1998) show that neutrophil TEM by itself does not reduce resistance under control conditions. We have extended the number of references on this topic in the discussion section.

Other Comments:

3. Sites of TEM could differentially impact permeability. Do the neutrophils use the paracellular (junctional) or non-junctional routes of TEM in these assays? This is physiologically relevant because different routes of TEM (junction vs non-junctional) may differentially impact barrier function. Older literature found that longer times of TNF treatment led to greater amounts of ICAM-1 and a significant increase in non-junctional TEM. Did Authors observe non-junctional TEM at hot spots?

We agree with the reviewer that paracellular and transcellular TEM could potentially impact permeability in different ways. To answer the reviewers' questions, we share some yet unpublished data from our lab showing that neutrophils almost exclusively take the paracellular route in our *in vitro flow* assay. We tested 4, 20, and 24 hours of TNFα incubation, and found hardly any transcellular TEM events under these conditions. These data contrast with other leukocyte subsets, as monocytes, and CD4+ and CD8+ T-cells did have very substantial percentages of transcellular TEM events. These data are added for the reviewer (R1_Figure 3) [Figure for referees not shown.] .

4. Authors ignore other possible contributors to neutrophil TEM hotspots: E-selectin is inducible in parallel with ICAM-1, and E-selectin assists in activating neutrophil LFA-1 integrins to bind ICAM-1 and mediate arrest and adhesion/migration to TEM sites. Authors focus on ICAM-1 but clearly other molecules play important roles. Including the surface expression/distribution of E-selectin in these TEM assay models would be important. Likewise prior reports found that intracellular pools/locations of chemokines play crucial roles in driving leukocyte TEM in vivo and in vitro (doi: 10.1016/j.immuni.2018.09.018; doi: 10.1038/ni.2173).

We thank the reviewer for highlighting this important point. We agree that including data about E-selectin strengthens the paper. Therefore, we went back to the bench and performed a new set of experiments to study the role of E-selectin in hotspot formation. Using fluorescent microscopy, we show that also E-selectin shows a heterogeneous distribution over the endothelial monolayer. However, E-selectin does not co-localize with ICAM-1 and its heterogeneity does not correlate with that of ICAM-1. These new data were added to the revised manuscript to Figure 2 and S2. In addition, we show that E-selectin localization does not mark hotspots areas (new data to Figure 3). We think this could be explained by the fact that ICAM-1 and E-selectin play a role during different stages of TEM, and that these stages, the firm adhesion and rolling steps respectively, usually not take place at the same endothelial cell. We have adjusted the text in the revised manuscript as well. To further explore the relevance of E-selectin heterogeneity would be very interesting but is outside of the scope of this paper.

We are familiar with the work on chemokine deposits in TEM, and we agree it would be very interesting to study differences in chemokine presentation on a single-cell level. Our previous study shows that ACKR1 is present at so-called junction membrane protrusions, in line with the work by Girbl and colleagues (Immunity, 2018, cited by reviewer). This indicates that indeed chemokines may also play a role in the determination of hotspots (Figure 3E from Arts et al., eLife, 2021). We have extended our discussion with a paragraph on the potential role of chemokine sin hotspot formation. We also included the 2 papers that were cited by the reviewer.

5. Figure 2 and Sfig 2. Please address the following comment in Discussion: One possible explanation for heterogeneity of ICAM-1 or VCAM-1expression is that your HUVEC cultures were prepared from multiple umbilical cords, often up to 20+ or more at commercial suppliers of HUVEC. Do you see the same heterogeneity in a culture from a single cord donor?

Indeed, a possible explanation for the observed ICAM-1 heterogeneity could be donor variability within our HUVECs. We stained our BOECs, which have been grown from a single donated umbilical cord, for ICAM-1 and we do observe ICAM-1 heterogeneity here too (R1_Figure 4). Additionally, we see ICAM-1 heterogeneity in our *ex vivo* patient samples. We have addressed this issue with an extra comment in the discussion section.

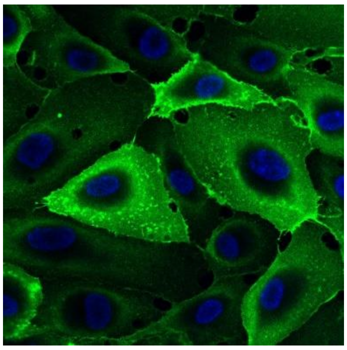

R1_Figure 4. Endothelial cells cultured from umbilical cord blood from a single donor show a heterogeneous expression of ICAM-1 in green. Nuclei are stained in blue.

6. Figure legend 1 and page 6 Results. Include length of time that EC were treated with TNF-alpha. Longer times of treatment (> 24h) vs shorter treatment times (4 - 6 h) leads to greater amounts of surface ICAM-1. It is more physiological that using transfection and CRISPR editing

We apologize for not including TNFα incubation times more clearly in our figure supplements. We have added this to the revised manuscript.

7. Fig S2 Images of discarded human surgical tissue stained for ICAM-1/PECAM-1 should include quantification.

We agree; a quantification would strengthen the point that we also observe heterogeneity in our patient samples. We performed manual quantification of all clearly distinguishable ECs in our images and added a graph displaying the heterogeneity of ICAM-1 expression in our *ex vivo* samples to Figure 3E and S2H.

Minor.
Ref 8 is incomplete?
Line 273, mislabeled (Fig 7A-B)?
Line 326, misspelled recognition.

We thank the reviewer for catching these mistakes and we have corrected them in the manuscript.

We thank reviewer 2 for his/her thoughtful comments. We have addressed all the comments to the best of our ability in a point-by-point reply, listed below.

Referee #2:

The manuscript by Grönloh et al. analyzes the role of ICAM-1 in recognizing TEM hotspots for migrating neutrophils under inflammatory conditions and assess the significance of these hotspots in limiting endothelial permeability during neutrophil extravasation. Using a combination of approaches, including Crispr/Cas9, ICAM-1 mutants, photo-convertible probe 36 mEos4b, and confocal and time lapse microscopy, neutrophil transmigration and endothelial permeability assays, data are presented to reveal the existence of neutrophil TEM hotspots in the endothelial monolayer that are regulated by ICAM-1 heterogeneity to limit vascular leakage during neutrophil extravasation. Evidence is also presented to show that the first 3 extracellular Ig-like domains of ICAM-1 are required whereas the intracellular tail of ICAM-1 is dispensable for hotspot recognition.

Although these findings are new and potentially interesting, the consideration of following points would further enhance the manuscript:

1. Increased ICAM-1 expression either by treatment with TNF or transfection of ICAM-1 cDNA each induces TEM hotspots. However, these experiments don't distinguish the additional effects of TNF (in addition to increasing the levels of ICAM-1) on ICAM-1 (confirmational changes or post-translational modifications) that may also contribute to ICAM-1-dependent TEM hotspots. It would be interesting to re-express ICAM-1 in ICAM-1 KO endothelial cells and treat them with or without TNF and monitor ICAM-1 heterogeneity and TEM hotspots to see if these effects are more pronounced in TNF-treated cells.

We thank the reviewer for this interesting point. We went back to the bench and performed the experiment suggested by the reviewer. We re-expressed ICAM-1-GFP in the ICAM-1 KO endothelial cells, treated those with and without TNFα and performed flow experiments with primary human neutrophils. The data show that neutrophils hardly adhered to ECs that were not treated with TNFα, despite the presence of exogenous ICAM-1. We have added these new data to the manuscript as Figure S4. These data underscore a previous report by Shulman and colleagues, who showed that overexpressing ICAM-1 in unstimulated HUVECs is not enough to allow T cell TEM (Shulman et al., Nat Immunol, 2012). Only when they co-expressed CCR2, T-cell TEM was rescued in uninflamed ECs.

The reviewer makes an interesting point regarding the potential effect of TNFα on post-translational modifications and conformational changes. Indeed, several ICAM-1 isoforms exist, but to our knowledge, it is still ill-defined as to how different stimuli affect the expression of each ICAM-1 isoform (Singh et al., Immunol Lett, 2021). Interestingly, after TNFα treatment, two ICAM-1 bands can be detected with Western blotting: a fully processed form and a hypo-glycosylated high-mannose form, which have been suggested to function in a different way than the full-length ICAM-1 does (Scott et al., Am J Physiol Cell Physiol. 2013). Checking whether these two variants could have different effects on hotspots would be interesting but are outside the scope of this paper. Nevertheless, we added this idea to the discussion section of the revised Ms.

2. Inclusion of an experiment in which extracellular domain of ICAM-2 or VCAM-1 is replaced with that of ICAM-1 (containing all five Ig-like domains or just first 3 domains) would not only strengthen the claim that ICAM-1 extracellular domain is not only sufficient but can render VCAM-1 or ICAM-2 capable of regulating TEM hotspots and limiting vascular permeability during neutrophil TEM.

We understand the point raised by the reviewer. We have extended our studies with other ICAM-1 extracellular domain mutants, i.e., we performed leakage experiments with ICAM-1 mutants that lacked only the first or third domain, to investigate the extracellular domains of ICAM-1 further. These leakage experiments proved very interesting, as we observed that even though the single domain truncations reduce preferential adhesion significantly individually (Figure 6A), the presence of either the first or the third domain is enough to prevent leakage (Figure 6C). However, we did not swap the entire extracellular domains of VCAM-1 or ICAM-2 with ICAM-1. One reason is that we already showed that ICAM-1 without any intracellular tail is enough for hotspot formation (Figure 6A). While we agree that creating such a chimeric mutant would be an elegant method to show that the extracellular domains of ICAM-1, when combined with VCAM-1 or ICAM-2 intracellular tails, could rescue hotspot formation, we also think our current data already proves this point.

3. It would be interesting to determine how endothelial permeability during TEM is affected when neutrophils treated with blocking anti-LFA-1 or anti-Mac-1 antibody alone and in combination are used.

We agree with the reviewer it would be interesting to study the role of the ICAM-1 ligands, LFA-1 and Mac-1, for the hotspot formation and subsequent leakage during TEM in our system. We indeed did very similar experiments to the ones suggested here in which we used neutrophils from LAD-1 (leukocyte adhesion deficiency) patients. These patients have a defect CD18 integrin and therefore a non-functioning LFA-1 and Mac-1. We have added a figure for the reviewer (R2_Figure 1, same as Figure R1_Figure 1), where we show that these cells hardly adhere to TNFα-treated endothelial cells under flow conditions.

We concluded that functional beta2 integrins are crucial in the adhesion of the neutrophils to the endothelium. In contrast to the endothelial ligands of these integrins: there are several alternatives for ICAM-1 and ICAM-2, e.g., PECAM-1 or JAM-A to bind to. As we did not observe any adhesion, no hotspots were detected and we expect to have little if any, diapedesis events to do a proper Transwell leakage assay.

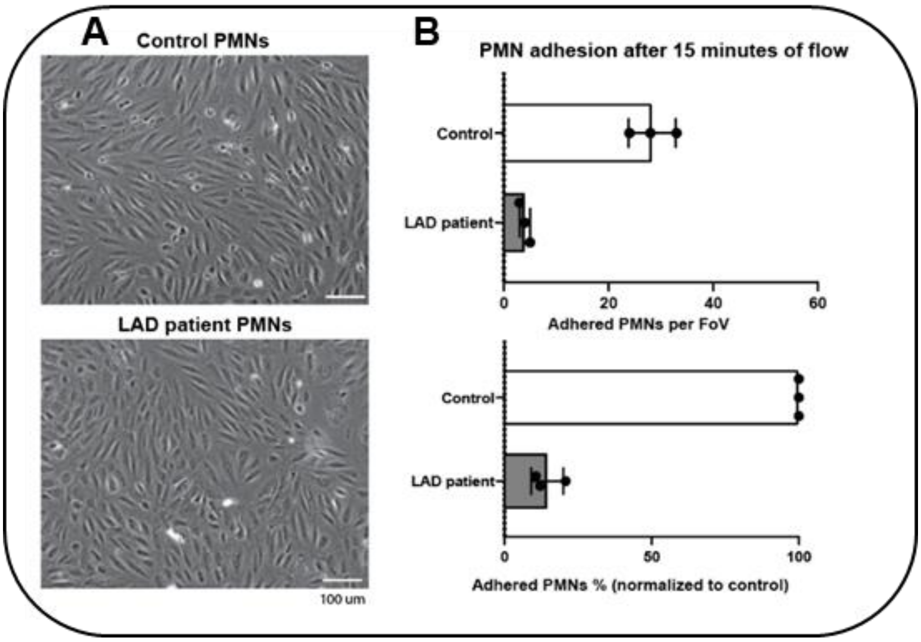

R2_Figure 1. Transmigration assay under flow conditions using wt and LAD1 neutrophils. A. Left panels to show endothelial monolayers after overnight TNFa treatment. Bar, 100 µm. B. Right graphs show quantification of the number of adherent neutrophils per field of view after 15 minutes of flow (upper graph), and a relative number of neutrophils that adhere to an endothelial monolayer in percentage (lower graph). The experiment is done three times independently of each other.

4. Experiments in Fig. 6C lacks data with ICAM-1 mutant lacking Ig-like domain 1 or 3 as controls.

We went back to the bench, generated the required mutants, and performed the leakage experiments with the Transwell system. These new data sets are added to the revised manuscript to Figures 6B and 6C.

5. The labels in Fig. 2A are barely legible.

We agree with the reviewer and have increased the text size to make the text more legible.

We thank reviewer 3 for his/her thoughtful comments. We have addressed all the comments to the best of our ability in a point-by-point reply, listed below.

Referee #3:

The manuscript by Grönloh and colleagues makes the argument that heterogeneous ICAM-1 expression on HUVEC or blood outgrowth ECs creates hotspots for PMN transmigration, and that in this configuration, TEM of neutrophils is not associated with an increase in dextran permeability, either during or after TEM. This study nicely quantifies many aspects of ICAM-1 and -2 dependent binding, crawling, and subsequent TEM of purified human neutrophils on human EC monolayers in vitro. The imaging methods and quantitative algorithms used have provided very interesting and novel data. There are a few minor details that need clarification. From my perspective, putting this data into the ICAM-1 and PECAM-1 dependent TEM literature, which generally has focused on signaling of TEM, without assessing permeability, requires a bit more integration and discussion.

Specific Comments:

1. The study makes the interesting observation that ICAM-1/2 hotspots promote TEM which somehow avoids a simultaneous increase in permeability to dextran (ie., paracellular permeability). So where are these hotspots in the monolayer? Are they near "tricellular junctions", for example? These have been referred to as the preferred sites of TEM, but with little documentation as to why, and thus addressing this old concept with new data seems relevant here. It would be interesting if results of the current study could clarify the location of hotspots relative to cell-cell junctions, especially PECAM staining, which I presume is required for TEM in the studies presented.

We agree with the reviewer that it would be interesting to not only focus on adhesion molecules but also take junction regions into account. It is known that upon adhesion, neutrophils typically spread out and start crawling towards a junction to transmigrate. On average, they take the first junction they encounter, which is always very near the initial adhesion spot. We previously reported on this phenomenon and showed that 85-90% of neutrophils undergo diapedesis at the first junction they encounter (Arts et al., eLife, 2021). Moreover, we have quantified the type of junction that is preferred by the neutrophil, discriminating between bi-, tri, and multi-cellular junctions. We have added this data set for the reviewer below (R3_Figure 1)[Figure for referees not shown.] . We have added a paragraph in the discussion section on this phenomenon, in particular on the possible existence of a second 'hotspot' after the initial hotspot we describe, in which neutrophils perhaps prefer certain types of junctional architecture for the diapedesis step of the TEM cascade.

To study if neutrophils prefer certain junctions, in particular with regards to PECAM-1 and VE-cadherin, we have performed photoconversion experiments for VE-cadherin and PECAM-1 and found that neutrophils preferred areas with a broader PECAM-1 stain. These data are part of another study, but we have included these data for the reviewer in R3_Figure 2 [Figure for referees not shown.] . From these data,

we conclude that neutrophils prefer junctions where more PECAM-1 is present or at least PECAM-1 is more broadly present across the junction, to cross the endothelial monolayer. Such differences were not observed for VE-cadherin.

2. Does EC ICAM-1 staining on individual cells change when the monolayer becomes confluent? Ie., Is formation of the "hotspots" driven by the patterning of the monolayer (e.g., associated with tricellular or other configuration?), or is it in inherent in the individual EC?

This is an interesting point raised by the reviewer. We went back to the bench and perform the suggested experiment and compared ICAM-1 heterogeneity of a confluent monolayer with ECs that were inflamed, fixed, and stained before a confluent monolayer was formed. Comparing confluent and sub-confluent ECs, we found no differences in heterogeneity, suggesting that there is at least some sort of inherent ICAM-1 expression level per individual endothelial cell. These new data were added to Figure S2.

3. Alternatively, or perhaps in addition, are the hotspots concentrated on individual cells such that "activation" of ICAM-1 can then drive mobilization of caveolae clusters to facilitate transmigration via a transcellular route or promote PECAM-1 membrane localization from the Lateral Border Recycling Compartment for binding and transmigration of neutrophils via the paracellular route. It was shown previously that ICAM-1 clustering induces NO and Src signaling that lead to ICAM-1, PECAM-1 and Cav1 phosphorylation. Some discussion on whether hotspots represent or give rise to clustered ICAM-1 that upon binding to PMN will promote their TEM via signaling that controls the transcellular or paracellular route. It was unexpected to this reviewer that the C-terminus of ICAM-1 would not be required for PMN TEM. Is this controversial ? Seems some discussion related to the role of the C-term ITAM motif on both ICAM-1 and 2 is warranted as this relates to the ability of TNF to not only increase ICAM-1 expression, but also the increase in phosphorylation-dependent binding avidity to enhance PMN binding and TEM.

We thank the reviewer for bringing this up. Indeed, we were surprised as well that the C-terminus of ICAM-1 did not play a role in the formation of hotspots. Clearly, the hotspot formation was at least in part mediated by the adhesion capacity of ICAM-1, not so much the signaling properties of ICAM-1. It was shown by previous studies, including ours, that ICAM-1 lacking the intracellular tail could still mediate adhesion, although indeed fewer neutrophils ended up below the endothelial monolayer in our Transwell experiments (Figure EV5). As earlier studies showed that removing the whole cytoplasmic domain, or specifically the RKIKK motif from the intracellular tail of ICAM-1 still maintains adhesion of leukocytes (Lyck *et al.,* 2003, Blood; Oh *et al*., 2007, MBOC*)*, but significantly decreases transmigration, we conclude that adhesion and hotspot recognition still work properly without the intracellular domain, but that probably the diapedesis step may be less efficient.

We show here that indeed, adhesion is intact and this in fact mediates hotspot formation. On average, we found that neutrophils crossed the endothelium at the first junction they encounter

(see data above, R3_Figure 1A). This indicates that the intrinsic capacity of an endothelial cell to display a hotspot lies within the same endothelial cell. We have elaborated on these interesting hypotheses in the discussion section and included important papers that refer to the intracellular tail of ICAM-1 (Lyck *et al.,* 2003, Blood; Oh *et al*., 2007, MBOC).

Indeed, as the reviewer suggests, the "activation" of ICAM-1 by increasing the concentration of the protein at sites of TEM may trigger the final diapedesis steps, and our data show that there is indeed a preference for enriched PECAM-1 expression, or at least "wide" PECAM-1 staining at junction regions. This may indicate a role for the LBRC, although we have not thoroughly studied this. Also, a role for caveolae cannot be excluded, as Millan and colleagues showed that enriched ICAM-1 regions supported transcellular migration through caveolae-rich areas, although we need to take into account that this study covered T-cell migration and not neutrophils (Millan et al., Nat Cell Biol, 2005). Also, in our flow model, it seems that neutrophil transcellular diapedesis on HUVECs is extremely rare, in contrast to monocytes and T cells (Figure R3_Figure 3)[Figure for referees not shown.] . Nevertheless, we believe these are interesting ideas that deserve to be discussed and therefore are included in the discussion section of the revised manuscript.

4. As there was no ICAM-1 KO effect on PMN TEM, is it your conclusion that TEM depends on PECAM-1 at cell-cell junctions? If so, what are CAMs are these PMN initially adhering to on the surface? ICAM-2 for sure. DKO of ICAM-1/2 did reduce TEM by 50%, but what other CAMs are participating in firm adhesion of PMN?

We show that there was some degree of inhibition of neutrophil TEM across ICAM-1 KO ECs, but this was not a clear decrease. Indeed, as the reviewer already points out, only when both ICAM-1 and -2 were depleted, significant inhibition of neutrophil TEM was measured. Other studies also show that depletion of ICAM-1 leads to only marginal inhibition of neutrophil TEM (Slingh et al., PNAS, 1993).

For T-cells, it was demonstrated that ICAM-1 deficiency leads to a drop in TEM (Lyck et al., Blood, 2003). We could confirm that (see R3_Figure 4, same figure as R1_Figure 1). So, it became clear that neutrophils can cross the EC independently from ICAM-1. We suggest that JAM-A or, as the reviewer already suggests, PECAM-1 may be among the candidates here. We have extrapolated this hypothesis in the discussion section of the revised Ms.

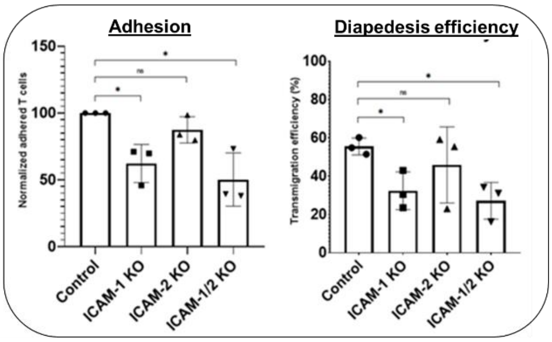

R3_Figure 4. Transmigration of CD8+ T cells across the endothelium. The left graph represents T-cell adhesion to endothelial cells that had an ICAM-1 KO, ICAM-2 KO, double KO or were left untreated (Control). The right graph shows diapedesis efficiency across ECs as described above**.**

5. Images of ICAM-1 and PECAM-1 in human microvessels do not seem to add anything or make a strong case in support of the in vitro studies. Also, what is the purpose of showing the vessel on a chip staining? This also does not seem to add information.

To add more significance to the *in vivo* data, we have quantified ICAM-1 heterogeneity and added it to the manuscript in Figure 2. For the vessel-on-a-chip images, it was our intention to show that ICAM-1 based EC heterogeneity was not only present in *in vitro* cultured but also in chip-related cultures, i.e., in a more physiologically relevant environment, comparing the 3D vs 2D difference with the more relevant substrate (a collagen matrix vs FN-coated plastics). Due to the inclusion of even more relevant new data, we have decided to move the vessel-on-a-chip to the supplemental figures.

6. The following sentence on pg 17 related to the method of neutrophil counting is confusing: "Only spots with a quality higher than 80 were filtered to ensure only neutrophils were counted." Where does this quality number come from?

We apologize for the unclear explanation regarding our data analysis pipeline. We have rephrased this part of the methods section. The quality threshold in the spot analysis pipeline was manually set to discriminate neutrophils from the background.

7. Monolayer permeability assessed by accumulation of 70kDa TR-dextran below the filter insert will reflect changes in junctional integrity based on molecule size and disruption of adherens or tight junctions (paracellular pathway). Considering ICAM-1 activation via PMNs or antibody crosslinking can stimulate Src and trafficking of caveolae by promoting Cav1 phosphorylation, it would perhaps be more interesting and for sure more physiological to assess Au-albumin permeability by transmission EM as a function of PMN TEM, and to relate this to paracellular vs transcellular transport of albumin.

For our permeability assays, we have used 70kDa Dextran polymers. We choose this polymer size as this is the size of albumin, indeed, as the reviewer referred to, one of the most abundant proteins in the plasma. In addition, to that, with neutrophil TEM, most of these cells cross the endothelium in a paracellular manner, i.e., through the cell-cell junctions. Even when we overexpress ICAM-1, we do this in an ICAM-1 KO background, thereby not necessarily increasing ICAM-1 expression levels and inducing transcellular migration. (Increased ICAM-1 level may lead to more transcellular migration; Yang et al., Blood, 2005). Therefore, we do not expect the transcellular pathway to be activated in our system.

8. A conclusion of this study could be that heterogeneous ICAM-1 expression prevents activated PMN from increasing monolayer permeability to macromolecules. Is there evidence for this in the ICAM-1-/- mouse? i.e., Do activated PMN increase endothelial permeability to a greater extent in absence of ICAM-1 expression?

The reviewer brings up an interesting point. Earlier work looking at the ICAM-1 KO mouse model showed only a limited reduction in neutrophil TEM, but no data on permeability can be found (Slingh et al., PNAS, 1993). Work from the Engelhardt group using ICAM-1 KO mice did not report increased permeability during TEM (Steiner et al., J Immunol, 2010; Lyck et al., Blood, 2003). In the Steiner et al paper they show that ICAM-1 KO does not alter vascular permeability of the

blood-brain barrier endothelium. In addition, it needs to be noted that they used specific brain models to study the blood-brain barrier and specifically focused on T-cell TEM. To be able to compare the data from our study, one should look at neutrophil TEM tissues like the cremaster or peritoneum. We have included this information in the discussion section of the revised manuscript.

Dear Prof. van Buul,

Thank you for the submission of your revised manuscript to our editorial offices. I have received the reports from the three referees that I asked to re-evaluate your study, you will find below. As you will see, the referees now fully support the publication of your study in EMBO reports.

Before we can proceed with formal acceptance, I have these editorial requested ask you to address in a final revised version of the manuscript:

- Please restrict the keywords on the title page to six.

- We now use CRediT to specify the contributions of each author in the journal submission system. CRediT replaces the author contribution section. Please use the free text box to provide more detailed descriptions. Thus, please remove the author contributions section from the manuscript text file. See also guide to authors:
https://www.embopress.org/page/journal/14693178/authorguide#authorshipguidelines

- Please make sure that all the funding information is also entered into the online submission system and that it is complete and similar to the one in the acknowledgement section of the manuscript text file. Presently, the Landsteiner Foundation for Blood Transfusion Research (LSBR) 1649 and 1820 have been entered into the submission system but are not mentioned in the manuscript text file.

- Please make sure that the number "n" for how many independent experiments were performed, their nature (biological versus technical replicates), the bars and error bars (e.g. SEM, SD) and the test used to calculate p-values is indicated in the respective figure legends (main, EV and Appendix figures), and that statistical testing has been done where applicable. Please avoid phrases like 'independent experiment', but clearly state if these were biological or technical replicates. Please add complete statistical testing to all diagrams (main, EV and Appendix figures). Please also indicate (e.g. with n.s.) if testing was performed, but the differences are not significant. In case n=2, please show the data as separate datapoints without error bars and statistics.

- We updated our journal's competing interests policy in January 2022 and request authors to consider both actual and perceived competing interests. Please review the policy https://www.embopress.org/competing-interests and update your competing interests if necessary. Please name this section 'Disclosure and Competing Interests Statement' and put it after the Acknowledgements section.

- Please order the manuscript sections like this, using these names:
Title page - Abstract - Key Words - Introduction - Results - Discussion - Materials and Methods - Data availability section - Acknowledgements - Author contributions - Disclosure and Competing Interests Statement - References - Figure legends - Expanded View Figure legends
- Please also note our reference format (10 authors et al.):
http://www.embopress.org/page/journal/14693178/authorguide#referencesformat

- Please name the EV figures "Figure EVx" in the Expanded View Figure legends.

- Please callout the Appendix figures using the nomenclature "Appendix Figure Sx".

- Appendix Figs. S5, S6 and S8 only have one panel. Please remove the "A" from the figure, the legend and from the respective callouts in the manuscript text.

- Please make sure that all figure panels are called out sequentially and separately. Presently, there seems to be no callout for Fig. 5F. Please provide separate callouts for the panels of Appendix Fig. S1.

- Finally, please find attached a word file of the manuscript text (provided by our publisher) with changes we ask you to include in your final manuscript text, and some queries, we ask you to address. Please provide your final manuscript file (using the attached file as basis) with track changes, in order that we can see any modifications done.

In addition, I would need from you:
- a short, two-sentence summary of the manuscript (not more than 35 words).
- three to four short bullet points highlighting the key findings of your study (two lines each).
- a schematic summary figure (in jpeg or tiff format with the exact width of 550 pixels and a height of not more than 400 pixels) that can be used as a visual synopsis on our website.

I look forward to seeing the final revised version of your manuscript when it is ready. Please let me know if you have questions

regarding the revision.

Best,

Achim Breiling
Senior Editor
EMBO Reports

Referee #1:

The revised paper looks good and provides new, unexpected data on the role of neutrophil adhesion "hot spots" in endothelial barrier function. Authors rebuttal letter has addressed my concerns, however, the Authors did not identify their changes/revisions in the text of submitted version i downloaded, so i am not sure if i was able to confirm that all of their indicated changes were made. I will leave that to Editors to decide if this needs to be addressed, or if other reviewers have similar comment.

Referee #2:

The authors have adequately addressed my concerns.

Referee #3:

The authors have addressed my comments thoroughly and I have no further concerns.

The authors addressed the minor editorial issues.

Prof. Jaap van Buul
Sanquin Research and Landsteiner Laboratory
Vascular Cell Biology
Plesmanlaan 125
Plesmanlaan 125
Amsterdam, Noord Holland 1066CX
Netherlands

Dear Prof. van Buul,

I am very pleased to accept your manuscript for publication in the next available issue of EMBO reports. Thank you for your contribution to our journal.

At the end of this email I include important information about how to proceed. Please ensure that you take the time to read the information and complete and return the necessary forms to allow us to publish your manuscript as quickly as possible.

As part of the EMBO publication's Transparent Editorial Process, EMBO reports publishes online a Review Process File to accompany accepted manuscripts. As you are aware, this File will be published in conjunction with your paper and will include the referee reports, your point-by-point response and all pertinent correspondence relating to the manuscript.

If you do NOT want this File to be published, please inform the editorial office within 2 days, if you have not done so already, otherwise the File will be published by default [contact: emboreports@embo.org]. If you do opt out, the Review Process File link will point to the following statement: "No Review Process File is available with this article, as the authors have chosen not to make the review process public in this case." Please note that the author checklist will still be published even if you opt out of the transparent process.

Thank you again for your contribution to EMBO reports and congratulations on a successful publication. Please consider us again in the future for your most exciting work.

Yours sincerely,

Achim Breiling
Editor
EMBO Reports

****

THINGS TO DO NOW:

Once your article has been received by Wiley for production, the corresponding author will receive an email from Wiley's Author Services system which will ask them to log in and will present them with the appropriate license for completion.

You will receive proofs by e-mail approximately 2-3 weeks after all relevant files have been sent to our Production Office; you should return your corrections within 2 days of receiving the proofs.

Please inform us if there is likely to be any difficulty in reaching you at the above address at that time. Failure to meet our deadlines may result in a delay of publication, or publication without your corrections.

All further communications concerning your paper should quote reference number EMBOR-2022-55483V3 and be addressed to emboreports@wiley.com.

Should you be planning a Press Release on your article, please get in contact with emboreports@wiley.com as early as possible, in order to coordinate publication and release dates.

EMBO Press Author Checklist

Corresponding Author Name:	
Journal Submitted to:	
Manuscript Number:	

USEFUL LINKS FOR COMPLETING THIS FORM
[The EMBO Journal - Author Guidelines]
[EMBO Reports - Author Guidelines]
[Molecular Systems Biology - Author Guidelines]
[EMBO Molecular Medicine - Author Guidelines]

Reporting Checklist for Life Science Articles (updated January 2022)
This checklist is adapted from Materials Design Analysis Reporting (MDAR) Checklist for Authors. MDAR establishes a minimum set of requirements in transparent reporting in the life sciences (see Statement of Task: 10.31222/osf.io/9sm4x). Please follow the journal's guidelines in preparing your manuscript.
Please note that a copy of this checklist will be published alongside your article.

Abridged guidelines for figures
1. Data
The data shown in figures should satisfy the following conditions:
- the data were obtained and processed according to the field's best practice and are presented to reflect the results of the experiments in an accurate and unbiased manner.
- ideally, figure panels should include only measurements that are directly comparable to each other and obtained with the same assay.
- plots include clearly labeled error bars for independent experiments and sample sizes. Unless justified, error bars should not be shown for technical replicates.
- if n<5, the individual data points from each experiment should be plotted. Any statistical test employed should be justified.
- Source Data should be included to report the data underlying figures according to the guidelines set out in the authorship guidelines on Data Presentation.

2. Captions
Each figure caption should contain the following information, for each panel where they are relevant:
- a specification of the experimental system investigated (eg cell line, species name).
- the assay(s) and method(s) used to carry out the reported observations and measurements.
- an explicit mention of the biological and chemical entity(ies) that are being measured.
- an explicit mention of the biological and chemical entity(ies) that are altered/varied/perturbed in a controlled manner.
- the exact sample size (n) for each experimental group/condition, given as a number, not a range;
- a description of the sample collection allowing the reader to understand whether the samples represent technical or biological replicates (including how many animals, litters, cultures, etc.).
- a statement of how many times the experiment shown was independently replicated in the laboratory.
- definitions of statistical methods and measures:
  - common tests, such as t-test (please specify whether paired vs. unpaired), simple χ2 tests, Wilcoxon and Mann-Whitney tests, can be unambiguously identified by name only, but more complex techniques should be described in the methods section;
  - are tests one-sided or two-sided?
  - are there adjustments for multiple comparisons?
  - exact statistical test results, e.g., P values = x but not P values < x;
  - definition of 'center values' as median or average;
  - definition of error bars as s.d. or s.e.m.

Please complete ALL of the questions below.
Select "Not Applicable" only when the requested information is not relevant for your study.

Materials

Newly Created Materials	Information included in the manuscript?	In which section is the information available? (Reagents and Tools Table, Materials and Methods, Figures, Data Availability Section)
New materials and reagents need to be available; do any restrictions apply?	Not Applicable	

Antibodies	Information included in the manuscript?	In which section is the information available? (Reagents and Tools Table, Materials and Methods, Figures, Data Availability Section)
For **antibodies** provide the following information:<br>- Commercial antibodies: RRID (if possible) or supplier name, catalogue number and or/clone number<br>- Non-commercial: RRID or citation	Yes	Materials and Methods, p 15-16

DNA and RNA sequences	Information included in the manuscript?	In which section is the information available? (Reagents and Tools Table, Materials and Methods, Figures, Data Availability Section)
**Short novel DNA or RNA including primers, probes**: provide the sequences.	Yes	Appendix table 1

Cell materials	Information included in the manuscript?	In which section is the information available? (Reagents and Tools Table, Materials and Methods, Figures, Data Availability Section)
**Cell lines:** Provide species information, strain. Provide accession number in repository **OR** supplier name, catalog number, clone number, and/**OR** RRID.	Yes	Materials and methods, p16
**Primary cultures:** Provide species, strain, sex of origin, genetic modification status.	Not Applicable	
Report if the cell lines were recently **authenticated** (e.g., by STR profiling) and tested for mycoplasma contamination.	Yes	Materials and methods, p16

Experimental animals	Information included in the manuscript?	In which section is the information available? (Reagents and Tools Table, Materials and Methods, Figures, Data Availability Section)
**Laboratory animals or Model organisms:** Provide species, strain, sex, age, genetic modification status. Provide accession number in repository **OR** supplier name, catalog number, clone number, **OR** RRID.	Not Applicable	
**Animal observed in or captured from the field:** Provide species, sex, and age where possible.	Not Applicable	
Please detail **housing and husbandry conditions**.	Not Applicable	

Plants and microbes	Information included in the manuscript?	In which section is the information available? (Reagents and Tools Table, Materials and Methods, Figures, Data Availability Section)
**Plants:** provide species and strain, ecotype and cultivar where relevant, unique accession number if available, and source (including location for collected wild specimens).	Not Applicable	
**Microbes:** provide species and strain, unique accession number if available, and source.	Not Applicable	

Human research participants	Information included in the manuscript?	In which section is the information available? (Reagents and Tools Table, Materials and Methods, Figures, Data Availability Section)
If collected and within the bounds of privacy constraints report on age, sex and gender or ethnicity for all study participants.	Yes	Materials and Methods

Core facilities	Information included in the manuscript?	In which section is the information available? (Reagents and Tools Table, Materials and Methods, Figures, Data Availability Section)
If your work benefited from core facilities, was their service mentioned in the acknowledgments section?	Not Applicable	

Design

Study protocol	Information included in the manuscript?	In which section is the information available? (Reagents and Tools Table, Materials and Methods, Figures, Data Availability Section)
If study protocol has been **pre-registered, provide DOI in the manuscript**. For clinical trials, provide the trial registration number **OR** cite DOI.	Not Applicable	
Report the **clinical trial registration number** (at ClinicalTrials.gov or equivalent), where applicable.	Not Applicable	

Laboratory protocol	Information included in the manuscript?	In which section is the information available? (Reagents and Tools Table, Materials and Methods, Figures, Data Availability Section)
Provide DOI OR other citation details if **external detailed step-by-step protocols** are available.	Not Applicable	

Experimental study design and statistics	Information included in the manuscript?	In which section is the information available? (Reagents and Tools Table, Materials and Methods, Figures, Data Availability Section)
Include a statement about **sample size** estimate even if no statistical methods were used.	Yes	Figure legends, p33-44
Were any steps taken to minimize the effects of subjective bias when allocating animals/samples to treatment (e.g. **randomization procedure**)? If yes, have they been described?	Yes	Materials and methods, p18
Include a statement about **blinding** even if no blinding was done.	Yes	Materials and methods, p24-25
Describe **inclusion/exclusion criteria** if samples or animals were excluded from the analysis. Were the criteria pre-established?<br><br>If sample or data points were omitted from analysis, report if this was due to attrition or intentional exclusion and provide justification.	Not Applicable	
For every figure, are **statistical tests** justified as appropriate? Do the data meet the assumptions of the tests (e.g., normal distribution)? Describe any methods used to assess it. Is there an estimate of variation within each group of data? Is the variance similar between the groups that are being statistically compared?	Yes	Figure legends and materials and methods, p33-44

Sample definition and in-laboratory replication	Information included in the manuscript?	In which section is the information available? (Reagents and Tools Table, Materials and Methods, Figures, Data Availability Section)
In the figure legends: state number of times the experiment was **replicated** in laboratory.	Yes	Figure legends, p33-44
In the figure legends: define whether data describe **technical or biological replicates**.	Yes	Figure legends, p33-44

Ethics

Ethics	Information included in the manuscript?	In which section is the information available? (Reagents and Tools Table, Materials and Methods, Figures, Data Availability Section)
Studies involving **human participants**: State details of **authority granting ethics approval** (IRB or equivalent committee(s), provide reference number for approval.	Yes	Materials and methods, p17&p21-22
Studies involving **human participants**: Include a statement confirming that **informed consent** was obtained from all subjects and that the experiments conformed to the principles set out in the WMA Declaration of Helsinki and the Department of Health and Human Services Belmont Report.	Yes	Materials and methods,p17&p21-22
Studies involving **human participants:** For publication of **patient photos**, include a statement confirming that consent to publish was obtained.	Not Applicable	
Studies involving experimental **animals**: State details of **authority granting ethics approva**l (IRB or equivalent committee(s), provide reference number for approval. Include a statement of compliance with ethical regulations.	Not Applicable	
Studies involving **specimen and field samples:** State if relevant **permits** obtained, provide details of authority approving study; if none were required, explain why.	Not Applicable	

Dual Use Research of Concern (DURC)	Information included in the manuscript?	In which section is the information available? (Reagents and Tools Table, Materials and Methods, Figures, Data Availability Section)
Could your study fall under dual use research restrictions? Please check biosecurity documents and list of **select agents and toxins** (CDC): https://www.selectagents.gov/sat/list.htm	Not Applicable	
If you used a select agent, is the security level of the lab appropriate and reported in the manuscript?	Not Applicable	
If a study is subject to dual use research of concern regulations, is the name of the **authority granting approval and reference number** for the regulatory approval provided in the manuscript?	Not Applicable	

Reporting

The MDAR framework recommends adoption of discipline-specific guidelines, established and endorsed through community initiatives. Journals have their own policy about requiring specific guidelines and recommendations to complement MDAR.

Adherence to community standards	Information included in the manuscript?	In which section is the information available? (Reagents and Tools Table, Materials and Methods, Figures, Data Availability Section)
State if relevant guidelines or checklists (e.g., **ICMJE, MIBBI, ARRIVE, PRISMA**) have been followed or provided.	Not Applicable	
For **tumor marker prognostic studies**, we recommend that you follow the **REMARK** reporting guidelines (see link list at top right). See author guidelines, under 'Reporting Guidelines'. Please confirm you have followed these guidelines.	Not Applicable	
For **phase II and III randomized controlled trials**, please refer to the **CONSORT** flow diagram (see link list at top right) and submit the CONSORT checklist (see link list at top right) with your submission. See author guidelines, under 'Reporting Guidelines'. Please confirm you have submitted this list.	Not Applicable	

Data Availability

Data availability	Information included in the manuscript?	In which section is the information available? (Reagents and Tools Table, Materials and Methods, Figures, Data Availability Section)
Have **primary datasets** been deposited according to the journal's guidelines (see 'Data Deposition' section) and the respective accession numbers provided in the Data Availability Section?	Not Applicable	
Were **human clinical and genomic datasets** deposited in a public access-controlled repository in accordance to ethical obligations to the patients and to the applicable consent agreement?	Not Applicable	
Are **computational models** that are central and integral to a study available without restrictions in a machine-readable form? Were the relevant accession numbers or links provided?	Not Applicable	
If publicly available data were reused, provide the respective **data citations in the reference list**.	Not Applicable	
